# Supplementary material for: Landfill leachate: An invisible threat to soil quality of temperate Himalayas
Source: PLoS One. 2024 Nov 19;19(11):e0314006. doi: 10.1371/journal.pone.0314006 (PMC11575778; doi:10.1371/journal.pone.0314006)
Supplement: Attachment — Submitted filename: PONE-D-24-19939_R2_reviewer_revised.pdf [file pone.0314006.s006.pdf]

PLOS ONE

Landfill leachate: an invisible threat to soil quality of temperate Himalayas

--Manuscript Draft--

**Manuscript Number:**	PONE-D-24-19939R2
**Article Type:**	Research Article
**Full Title:**	Landfill leachate: an invisible threat to soil quality of temperate Himalayas
**Short Title:**	Landfill leachate: an invisible threat to soil quality of temperate Himalayas
**Corresponding Author:**	Asif Malik<br>SKUAST Kashmir: Sher-e-Kashmir University of Agricultural Sciences and Technology of Kashmir<br>SRINAGAR, JAMMU & KASHMIR INDIA
**Keywords:**	Landfill, Leachate, Soil quality, Heavy metals
**Abstract:**	Landfills are the most affordable and popular method for managing waste in many parts of the world, However, in most developing nations, including India, the infiltration of hazardous materials from improperly managed dumping sites continues to be a significant environmental problem. Around the world, leachate is a significant point source of contamination in numerous environmental media, including soil, groundwater, and surface water. Soil is an important asset as they are the key factors for food production and have tremendous significance in achieving sustainable development goals (SDGs). The contaminants from soil enter into food chain and ultimately reach humans. So in order to prevent the adverse effects of toxic elements on humans, there is need to maintain the soil quality and to prevent deterioration. Keeping in view the consequences of unscientific management of waste, the goal of the experiment was to determine how landfill leachate from Achan landfill affected the soil quality in the temperate Himalayas. All four seasons of the year, viz Spring, Summer, Autumn, and Winter, at four sites viz, Center of dumping site, inside, Outside and Control were monitored. Among sites center was found to have maximum value of EC (3.04 dS/m), Moisture content (42.51%), N (285.43 mg/kg), P (70.07 mg/kg), K (265.71 mg/kg), Ca (957.67 mg/kg), Mg(402.42 mg/kg), Zn (2.02 mg/kg), Fe (10.56 mg/kg), Cu (2.07 mg/kg), Mn (10.73 mg/kg), Pb (85.02 mg/kg), Cd (4.50 mg/kg), Ni (29.04 mg/kg), Cr (23.37 mg/kg), As (14.10 mg/kg). While as the lowest value of all parameters was reported at control site. Among seasons summer was reported to have maximum value of EC (1.818 dS/m), N (264.11 mg/kg), P (60.98 mg/kg), K (239.70 mg/kg), Ca (913.52 mg/kg), Mg (385.09 mg/kg), Pb (72.1 mg/kg), Cd (4.46 mg/kg), Ni (29.70 mg/kg), Cr (22.41 mg/kg), As (12.63 mg/kg), While as lowest value of these parameters was reported during winter season. Among sites Minimum pH (6.2) was reported at center of dumping site and maximum (6.7) was reported at control site, while as among seasons maximum pH was reported during winter season (6.6) and minimum (6.3) was reported during summer season. Zn, Fe, Cu and Mn were reported maximum during winter season and minimum during summer season. From the study it is recommended that the waste generated is mostly organic (65-75%), thus can be segregated and treated at source. The waste can be treated at source using microbial consortium technology in order to transform the waste in to wealth in a sustainable way and to prevent the soil quality deterioration
**Order of Authors:**	Shayesta Islam
	Haleema Bano
	Asif Aziz Malik
	Fahad Alotaibi
**Response to Reviewers:**	Review Comments to the Author Please use the space provided to explain your answers to the questions above. You may also include additional comments for the author, including concerns about dual publication, research ethics, or publication ethics. (Please upload your review as an attachment if it exceeds 20,000 characters)<br>Reviewer #2: (No Response)<br>Reviewer #3:<br>Dear Authors,

Powered by Editorial Manager® and ProduXion Manager® from Aries Systems Corporation

The subject of the study is interesting and topical, with scientific and practical importance. The authors took into account the recommendations of the reviewers and made the necessary corrections. The article has been considerably improved.
The following aspects are brought to the attention of the authors.
1.Article structure
Page 13, row 101
“Study area:”
Page 14
“Methodology:”
It is recommended to check and revise the structure of chapters (chapter titles), according to the one recommended in the Submission Guidelines, PLOS ONE.
e.g.
“Manuscript Organization”
“Materials and Methods”
Response: All the chapter titles and subtitles have been revised according to the guidelines of PLOS ONE
2.Writing ionic forms
Page 27, row 348
“Mg2+” instead of “Mg2+”
"2+" as index
Page 30, row 402
“Fe2O3” instead of “Fe2O3”
Page 31, row 404
“Fe2+” instead of “Fe++”
"2+" as index
row 409
“Fe3+” instead of “Fe3+”
"3+" as index
It is recommended to use the Word Equation Editor to write the ionic forms correctly
Response: All the corrections suggested have been made and word equation editor has been used to write ionic forms.
3. Presentation of the Figures
Figure 14 is presented on page 41
Until figure 14, no other figure is presented in the content of the article.
References are made to the other figures, but they are presented at the end, starting with page 53.
Please check and revise, if necessary, according to the Submission Guidelines, PLOS ONE
Response: According to submission guidelines of PLOS ONE, figures are not to be included in the main manuscript file, figures are to be prepared as individual file. The Fig 14 has now also been removed from main manuscript. The captions of all figures have been inserted in the text of manuscript immediately after the paragraph in which it is cited.
4. References Please check the References chapter, and revise, if necessary, according to the Submission Guidelines, PLOS ONE
Response: References have been revised according to submission guidelines
Reviewer #4: PLOS ONE- Landfill leachate: an invisible threat to soil quality of temperate Himalayas
Manuscript Number: PONE-D-24-19939R1
Overall comments
The manuscript is informative. I am very positive about the manuscript, but it still requires additional work and minor editing before being considered for publication. Please ensure that your manuscript meets PLOS ONE's style requirements. The paper needs a total revision; especially the results section and the discussion section should be separate.
Response: Separated result and discussion section
Please review your reference list to ensure that it is complete and correct. Please follow the references style of plos journal formet. Provide DOI no. of the references and try to add recent references. For example ‘Amoakwah E, Arthur E, Frimpong KA, Islam KR. Biochar Amendment Influences Tropical Soil Carbon and Nitrogen Lability. Journal of Soil Science and Plant Nutrition. 2021; 21: 3567– 3579. http://dx.doi.org/10.1007/s42729-021-00628-4’’
Response: Revised reference section according to journal guidelines
Specific comments

Powered by Editorial Manager® and ProduXion Manager® from Aries Systems Corporation

1. Please check no. of words in Abstract, not exceed 300 words according to journal style. Please ensure that your manuscript meets PLOS ONE's style requirements
Response: Abstract has been reduced to 300 words only
2. Please insert the title of the figure or table just below the text. Please ensure that your manuscript meets PLOS ONE's style requirements
Response: Title of figures and tables have been inserted in the text as per journal guidelines.
3. It would be better to separate the results section and the discussion section. Please check the PLOS ONE's style.
Response: Separated results and discussion section.
4. Rewrite the discussion section.
Response: Modified the discussion section
5. Please follow the reference style of PLOS ONE's style. Provide DOI no. of the references and try to add recent references. Please review your reference list to ensure that it is complete and correct. For example ‘’Amoakwah E, Arthur E, Frimpong KA, Islam KR. Biochar Amendment Influences Tropical Soil Carbon and Nitrogen Lability. Journal of Soil Science and Plant Nutrition. 2021; 21: 3567– 3579. http://dx.doi.org/10.1007/s42729-021-00628-4’’
Response: Revised reference section as per journal guidelines
6. Please check the front style of headline, sub-headline and text front. Please check the PLOS ONE's style.
Response: Followed PLOS one font style for each section.
7. If possible try to reduce no of figures. No. of fig 14 is too high.
Response: Reduced the number of figures to 9
8. Please check line no. 58. Remove double full stop.
Response: Done
9. Please check line no. 84. Close bracket
Response: Done
10. Please check line no. 90. Check the space
Response: Done
11. Please check line no. 108. Remove bracket
Response: Done
12. Please check line no. 121. Edit space
Response: Done
13. Please check line no. 121, 150, 121. Edit space
Response: Done
14. Please check line no. 312, 314, 352, and 354 and so on. Why are the numbers bold?
Response: Done
15. Please check Table 3, column 8. Some words are overlapped
Response: The line numbers have been overlapped with the table values, corrected
16. Please check line no. 474, 476,506. Please check the line spacing
Response: Done
17. Please provide declaration, consent for publication and conflict of interest from the manuscript and add in a separate file to PLOS ONE's online system. Only Acknowledgement and Author contributions would be attached with the article.
Response: Done as per suggestion
18. Please ensure that your manuscript meets PLOS ONE's style requirements.
Response: Modified as per journal guidelines
Reviewer #5: A brief study on "Landfill leachate: an invisible threat to soil quality of temperate Himalayas" has been conducted by the authors. The major concern is the content of toxic elements like Pb, Cd, Ni, As and Cr in the leachate. This will help the environmentalist and planner of the region for sustainable development goals for the locality. Please mention the safe limit of all the parameters studied in this work for soil just like Pb, Cd, Ni, As and Cr.
Response: Safe limits for all paramaters have been incorporated
Reviewer #6:
1. Abstract: Grammatical correction is required in lines- 15, 19, 29, 30, 33-36, 41. Punctuation correction is required in lines-30, 34.
Response: Corrected
2. Introduction: Too much narration on soil health in the introduction section (lines 54-64) while less description of the keywords - landfill and leachate. An individual paragraph on Achan landfill and leachate could be helpful for better understanding and reading. Coordination of the paragraphs in the introduction section is suggested.

Powered by Editorial Manager® and ProduXion Manager® from Aries Systems Corporation

	Grammatical and punctuation correction is required in all through the introduction section (lines- 45-47, 52,54,58, 60-68, 81, 82).<br>Response: Line numbers from 54-64 have been removed from the manuscript. Separate paragraph on Achan landfill and leachate have been added. Grammatical and punctuation errors have been corrected.<br>3. Study area: No indication of the area in the sampling site. Precise information about the sampling site is needed. Grammatical and punctuation correction is required in the study area section- 108,112,113<br>Response: Area under landfill have been added and corrections are made.<br>4. Methodology: No indication of year of sampling. Why sampling was done in those locations? Are those sites the leachate outflow sites? Authors analyzed only soils. But analysis of both soil and leachate would give a clear understanding about the impact of landfill leachate on soil quality. Grammatical and punctuation correction is required in the methodology section (lines- 120-122, 140)<br>Response: Mentioned year of sampling and reasons for soil sampling, Grammatical and punctuation correction done<br>5. Result and discussion: I suggest to establish a relationship among pH, EC and element concentration.<br>Response: Incorporated correlation table in results section (Table 7)<br>While revising your submission, please upload your figure files to the Preflight Analysis and Conversion Engine (PACE) digital diagnostic tool, https://pacev2.apexcovantage.com/. PACE helps ensure that figures meet PLOS requirements. To use PACE, you must first register as a user. Registration is free. Then, login and navigate to the UPLOAD tab, where you will find detailed instructions on how to use the tool. If you encounter any issues or have any questions when using PACE, please email PLOS at figures@plos.org. Please note that Supporting Information files do not need this step.<br>Response: Done
**Additional Information:**	
**Question**	**Response**
**Financial Disclosure**<br><br>Enter a financial disclosure statement that describes the sources of funding for the work included in this submission. Review the submission guidelines for detailed requirements. View published research articles from *PLOS ONE* for specific examples.<br><br>This statement is required for submission and **will appear in the published article** if the submission is accepted. Please make sure it is accurate.	The author(s) received no specific funding for this work.

**Funded studies**<br>Enter a statement with the following details:<br>• Initials of the authors who received each award<br>• Grant numbers awarded to each author<br>• The full name of each funder<br>• URL of each funder website<br>• Did the sponsors or funders play any role in the study design, data collection and analysis, decision to publish, or preparation of the manuscript?<br><br>Did you receive funding for this work?	
**Competing Interests**<br><br>Use the instructions below to enter a competing interest statement for this submission. On behalf of all authors, disclose any competing interests that could be perceived to bias this work—acknowledging all financial support and any other relevant financial or non-financial competing interests.<br><br>This statement is required for submission and **will appear in the published article** if the submission is accepted. Please make sure it is accurate and that any funding sources listed in your Funding Information later in the submission form are also declared in your Financial Disclosure statement.<br><br>View published research articles from *PLOS ONE* for specific examples.	The authors have declared that no competing interests exist.

**NO authors have competing interests**<br><br>Enter: *The authors have declared that no competing interests exist.*<br><br>**Authors with competing interests**<br><br>Enter competing interest details beginning with this statement:<br><br>*I have read the journal's policy and the authors of this manuscript have the following competing interests: [insert competing interests here]*<br><br>* typeset	
**Ethics Statement**<br><br>Enter an ethics statement for this submission. This statement is required if the study involved:<br><br>• Human participants<br>• Human specimens or tissue<br>• Vertebrate animals or cephalopods<br>• Vertebrate embryos or tissues<br>• Field research<br><br>Write "N/A" if the submission does not require an ethics statement.<br><br>General guidance is provided below. Consult the submission guidelines for detailed instructions. **Make sure that all information entered here is included in the Methods section of the manuscript.**	N/A

Powered by Editorial Manager® and ProduXion Manager® from Aries Systems Corporation

**Format for specific study types**<br><br>**Human Subject Research (involving human participants and/or tissue)**<br>• Give the name of the institutional review board or ethics committee that approved the study<br>• Include the approval number and/or a statement indicating approval of this research<br>• Indicate the form of consent obtained (written/oral) or the reason that consent was not obtained (e.g. the data were analyzed anonymously)<br><br>**Animal Research (involving vertebrate animals, embryos or tissues)**<br>• Provide the name of the Institutional Animal Care and Use Committee (IACUC) or other relevant ethics board that reviewed the study protocol, and indicate whether they approved this research or granted a formal waiver of ethical approval<br>• Include an approval number if one was obtained<br>• If the study involved *non-human primates*, add *additional details* about animal welfare and steps taken to ameliorate suffering<br>• If anesthesia, euthanasia, or any kind of animal sacrifice is part of the study, include briefly which substances and/or methods were applied<br><br>**Field Research**<br><br>Include the following details if this study involves the collection of plant, animal, or other materials from a natural setting:<br>• Field permit number<br>• Name of the institution or relevant body that granted permission	
**Data Availability**<br><br>Authors are required to make all data underlying the findings described fully available, without restriction, and from the time of publication. PLOS allows rare exceptions to address legal and ethical concerns. See the PLOS Data Policy and FAQ for detailed information.	Yes - all data are fully available without restriction

Powered by Editorial Manager® and ProduXion Manager® from Aries Systems Corporation

A Data Availability Statement describing where the data can be found is required at submission. Your answers to this question constitute the Data Availability Statement and **will be published in the article**, if accepted.<br><br>**Important:** Stating 'data available on request from the author' is not sufficient. If your data are only available upon request, select 'No' for the first question and explain your exceptional situation in the text box.<br><br>Do the authors confirm that all data underlying the findings described in their manuscript are fully available without restriction?	
**Describe where the data may be found in full sentences. If you are copying our sample text, replace any instances of XXX with the appropriate details.**<br><br>• If the data are **held or will be held in a public repository**, include URLs, accession numbers or DOIs. If this information will only be available after acceptance, indicate this by ticking the box below. For example: *All XXX files are available from the XXX database (accession number(s) XXX, XXX.*).<br>• If the data are all contained **within the manuscript and/or Supporting Information files**, enter the following: *All relevant data are within the manuscript and its Supporting Information files.*<br>• If neither of these applies but you are able to provide **details of access elsewhere**, with or without limitations, please do so. For example:<br><br>*Data cannot be shared publicly because of [XXX]. Data are available from the XXX Institutional Data Access / Ethics Committee (contact via XXX) for researchers who meet the criteria for access to confidential data.*<br><br>*The data underlying the results presented in the study are available from (include the name of the third party*	All relevant data are within the manuscript and its Supporting Information files.

and contact information or URL).<br>• This text is appropriate if the data are owned by a third party and authors do not have permission to share the data.<br><br>* typeset	
Additional data availability information:	Tick here if your circumstances are not covered by the questions above and you need the journal's help to make your data available.

Manuscript Click here to access/download;Manuscript;F-Manuscript Sec-Rev.docx

Landfill leachate: an invisible threat to soil quality of temperate Himalayas

Shayesta Islam[1¶], Haleema Bano[1¶], Malik Ahsaf Aziz[2*], Fahad Alotaibi[3&]

1: Division of Environmental Sciences, SKUAST K, Srinagar, Jammu and Kashmir, India

2: Division of Basic Science and Humanities, SKUAST K, Srinagar, Jammu and Kashmir, India

3: Department of Soil Science, College of Food and Agriculture Sciences, King Saud University, P.O.Box 2460, Riyadh, Saudi Arabia

*Corresponding author

Email id: drasifskuast@gmail.com (MAA)

[¶] These authors contributed equally to this work.

[&] This author also contributed equally to this work.

Abstract:

Landfills are the most affordable and popular method for managing waste in many parts of the world, However, in most developing nations, including India, the infiltration of hazardous materials from improperly managed dumping site continues to be a significant environmental problem. Around the world, leachate is a significant point source of contamination in numerous environmental media, including soil, groundwater, and surface water. Soil is an important asset as it is the key factor for food production and has tremendous significance in achieving sustainable development goals (SDGs). The contaminants from soil enter into food chain and ultimately reach humans. So in order to prevent the adverse effects of toxic elements on humans, there is need to maintain the soil quality and to prevent deterioration. Keeping in view the consequences of unscientific management of waste, the goal of the experiment was to determine how landfill leachate from Achan landfill affected the soil quality in the temperate Himalayas. All four seasons of the year, viz Spring, Summer, Autumn, and Winter, at four sites viz, Center of dumping site, inside, Outside and Control were monitored. Among sites center was found to

have maximum value of EC (3.04 dS/m), Moisture content (42.51%), N (285.43 mg/kg), P (70.07 mg/kg), K (265.71 mg/kg), Ca (957.67 mg/kg), Mg(402.42 mg/kg), Zn (2.02 mg/kg), Fe (10.56 mg/kg), Cu (2.07 mg/kg), Mn (10.73 mg/kg), Pb (85.02 mg/kg), Cd (4.50 mg/kg), Ni (29.04 mg/kg), Cr (23.37 mg/kg), As (14.10 mg/kg). While as the lowest value of all parameters was reported at control site. From the study it is recommended that the waste generated is mostly organic (65-75%), thus can be segregated and treated at source. The waste can be treated at source using microbial consortium technology in order to transform the waste in to wealth in a sustainable way and to prevent the deterioration of soil quality.

Key words: Landfill, Leachate, Soil quality, Heavy metals

Introduction:

Soil ecosystems are the foundation for food production and are crucial for achieving sustainable development goals (SDGs), so are regarded as significant environmental assets [1]. It supports or carries out a variety of tasks, such as biomass production, storing, filtering, and changing a wide variety of materials, including water and nutrients [2]. The majority of the carbon (C) and nutrient elements that support life are generated by soils, which also act as a repository for them [3]. Soils are also the pedestal that plants rely on to remain upright. They also serve as the habitat for a vast biodiversity and biomass of soil organisms [4]. Soils hold the water that plants and soil creatures need to exist and thrive, and they restrict the pace of water movement, limiting erosion and soil loss [5] It has been reported that improving soil health through various amendments like biochar application improves soil physicochemical properties and enzymatic activities [6]. But this valuable asset is currently under serious threat due to generation of excessive quantity of waste due to rapid population growth and urbanization. According to the reports of world bank, the world wide generation of municipal solid waste in

2018 was 1.3 billion tons per year and is expected to reach approximately 2.2 billion tons by 2025 [7]. Kashmir Valley is also grappling with significant challenges stemming from increasing waste generation. Srinagar city alone produces 0.526 kg of waste per capita per day, with Anantnag following at 0.479 kg, Ganderbal at 0.400 kg, and Budgam at 0.397 kg respectively. The total annual waste generated across these districts amounts to 57,199.99 Metric Tonnes (MT), with Srinagar producing the highest at 236,732.75 MT and Budgam the lowest at 42,840.00 MT [8]. This enormous amount of waste can pose great challenge especially to low- and middle-income countries and developing nations.

The immense volume of waste generated from various sources ultimately finds its way to landfills, with Srinagar city in the Kashmir Valley being no exception. Here, all the waste produced across the city is disposed of at the Achan landfill site, the sole dumping ground for Srinagar. This site, located in the northern part of the city, between the coordinates 34° 09′ N latitude and 74° 79′ E longitude, lies approximately 5-6 kilometers from the city's center. Before 1985, Srinagar produced minimal waste, free from the burden of plastic. Back then, the Srinagar Municipal Corporation (SMC) disposed of the city's waste either in the Noorbagh grounds or along the banks of the river Jhelum. However, with the advent of stricter environmental policies, the Government of Jammu and Kashmir, following state cabinet approval, transferred around 30.35 hectares (75 acres) of state land to the SMC for waste disposal at Achan [9]. By 1986, this site became the primary location for the open dumping of municipal solid waste. For many years, vast amounts of refuse were deposited here without consideration for the environmental consequences. Achan remained the sole dumping ground for Srinagar until 2008 [10], with waste from 518 collection points across the city being transported to this location [9]. Once a thriving wetland, home to vibrant birds and lush vegetation, Achan has since become a source of

pollution, contaminating water bodies, air, and soil. The nearby population has also been affected, suffering from various diseases. The waste deposited at the site undergoes biodegradation through a combination of physical, chemical, and biological processes, leading to the production of leachate—a dark brown liquid with a foul odor. This leachate contains dissolved organic and inorganic compounds, nutrients, suspended particles, heavy metals, and hazardous chemicals. When left untreated and uncontrolled, it poses a serious threat to natural and agricultural ecosystems, significantly degrading soil quality due to its high concentrations of nutrients, heavy metals, and soluble salts [11, 12].

Contaminated soil affects ecosystems and human, plant and animal health in a number of ways [13]. The acidic leachate formed due to production of organic acids liberated by the degradation of waste, leads to the acidic surroundings that destroys micro-organisms, which are helpful in improving the composition of the soil. Furthermore contaminated soil has the potential to alter agricultural metabolism and diminish crop yields by forcing trees and crops to absorb soil pollutants [14]. In addition to this, contaminated soils with high nitrogen and phosphorus levels will leach into waterways, leading in death of aquatic organisms by diminishing the amount of dissolved oxygen [15]. The metals in leachate are common environmental pollutants that are non-biodegradable, can deplete available soil resources, and have a negative impact on plant growth and yield [16, 17]..

Keeping in view the far reaching consequences of landfill leachate and contaminated soil and need for global attention on improving or restoring soil health, it is imperative to evaluate the affect of leachate on soil physico chemical properties for proper management strategy to prevent soil degradation and alleviate the threats posed by the expansion of waste. Understanding and estimating the quality of leachate-impacted soils can establish an opportunity to judge the

sustainability of land management and land-use systems. . In this context, the present study aimed to evaluate the influence of landfill leachate from Srinagar's Achan dump on soil physico-chemical parameters across different seasons and sites.

Study area

The study was conducted at the Achan landfill, located in Srinagar city, Jammu and Kashmir UT, at an elevation of 1600 meters above mean sea level. The landfill's coordinates range between 74°41' 6" and 74°57' 27" East Longitude and 33° 59' 14" and 34°12' 37" North Latitude, as shown in Fig 1.

Fig 1: Map of study area (Map prepared by USGS)

Spanning an area of 30.35 hectares (75 acres), the landfill is situated amidst residential settlements and water bodies, directly impacting the lives of residents in nine villages: Saidapora, Shonglipora, Waganpora, Sangam, Braywar, Danmar, Guzerbal, Noorshah Colony, and Bagh-i-Lal Pandith. Every day, the Srinagar Municipal Corporation (SMC) disposes of 450 metric tonnes in this site. Waste generation is projected to surge to 1,723 metric tonnes per day by 2035. Currently, 62% of the total waste produced daily in Srinagar consists of organic material, with 7% being plastic waste [18]. Approximately 65-70% of Srinagar's municipal solid waste is collected through door-to-door methods and street bin systems, and then transported to the Achan landfill. However, the remaining 30-35% is illegally discarded into depressions, river embankments, open spaces, or is burned locally by individuals and Safai Karamcharis. This not only causes a public nuisance but also creates breeding grounds for various diseases [19]. Due to inadequate waste management and improper handling, large heaps of waste have accumulated at the site, emitting a foul odor that disturbs nearby residents. During the rainy season, leachate

from the waste seeps into the soil, altering its physico-chemical properties and rendering the surrounding land unsuitable for agriculture.

Materials and methods

Soil samples were collected from the Achan dumpsite in Srinagar throughout all four seasons of 2022—spring, summer, autumn, and winter—following proper authorization from the Srinagar Municipal Corporation, headquartered at Kara Nagar, Srinagar, Jammu and Kashmir. Four distinct sampling sites were chosen: the center of the dumpsite, inside the dumpsite (four cardinal points within), outside the dumpsite (four cardinal points outside), and a control site located at the Shalimar campus of SKUAST Kashmir. These sites were selected to assess the impact of leachate produced from the waste on soil properties at varying distances from the dumping points. Three replicates were collected from each site.

Samples were taken solely from the soil, as leachate was directly seeping into the ground. The soil was collected in separate moisture cans to preserve its moisture content. Once collected, the samples were air-dried in the shade, then ground and sieved through a 2mm mesh before analyzing various physico-chemical parameters. Soil moisture content was determined using the gravimetric method by Prihar and Sandhu [20], while soil pH and electrical conductivity were measured using the potentiometric method with a pH meter and an electrical conductivity meter [21]. Nitrogen content was determined via the potassium permanganate method [22], and the available phosphorus was measured using the spectrophotometric method by Olsen et al. [23]. The Jackson method [21] was used to quantify available potassium, while calcium and magnesium levels were assessed using the Versenate method, as outlined by Chesin and Yein

[24]. Heavy metal concentrations were determined using the DTPA extraction process and an atomic absorption spectrophotometer (AAS), following the method of Lindsay and Norwell [25].

Statistical analysis

The data in the table are given as an interactive mean of sites and seasons in accordance with the usual technique outlined by Gomez and Gomez[26]. Two-way ANOVA (analysis of variance) was performed at a 5% level of significance to test for differences across sites and seasons. In the univariate statistical pairwise comparison between all the samples comparing the means of two populations that are seasons and sites in our situations, analysis of variance with post hoc testing (Duncan's multiple range test) is performed [27]. The significance is shown by the various letters, and vice versa [27].

Results

pH determines the acidity or alkalinity of soil solutions and refers to the soil's hydrogen ion content. In our study among seasons maximum mean pH was recorded in winter (6.6), followed by spring (6.5), autumn (6.4) and minimum was recorded in summer season (6.3). Among sites maximum mean pH was recorded in control (6.7), outside (6.6), inside (6.3) and minimum (6.2) was recorded at center as depicted in Table 1. The pH values varied significantly between summer, autumn, and winter, but there was no significant difference between spring and autumn. Additionally, the pH values at different locations—inside, outside, and control—were significantly different, though there was no significant difference between the center and inside locations. During this study mean EC of dumpsite soil was found to deviate from 0.27 dS/m to 3.43 dS/m. Maximum mean value of EC among seasons was recorded in summer (1.81 dS/m),

spring (1.66 dS/m), autumn (1.48 dS/m) and minimum was recorded in winter (1.35 dS/m). Among sites maximum mean value of EC was recorded at center (3.04 dS/m), followed by inside (2.01 dS/m), outside (0.86 dS/m) and minimum was recorded at control (0.40 dS/m). The EC values during spring, summer, autumn and winter were significantly different. In addition to this EC values at all sites were significantly different. In this study, the mean moisture content values ranged from 12.87% to 48.42%. Moisture content was recorded maximum in winter (36.95 %) among seasons, followed by spring (32.48%), autumn (29.34 %) and minimum was recorded in summer (25.23 %). Among sites mean maximum moisture content was recorded at center (42.51%), followed by inside (34.07 %), outside (28.83%) and minimum was recorded at control site (18.58%). The moisture content values between all seasons were significantly different, in addition this, the moisture content values between all sites were also significantly different.

Table 1. Seasonal and site wise variation in pH, EC (dS/m) and Moisture content (%) of soil

Parameters	Seasons	Sites				Mean	C.D (P≤0.05)
		Center	Inside	Outside	(Control)		
pH	Spring	6.3±0.05^{fgh}	6.4±0.09^{efg}	6.6±0.06^{bc}	6.8±0.10^{ab}	**6.5^{b}**	Sites: 0.12<br>Seasons: 0.12<br>Sites × Seasons: 0.24
	Summer	6.1±0.05^{h}	6.2±0.11^{gh}	6.4±0.06^{cdef}	6.5±0.14^{bcde}	**6.3^{c}**	
	Autumn	6.2±0.35^{gh}	6.3±0.09^{fgh}	6.5±0.04b^{cd}	6.7±0.18^{ab}	**6.4^{b}**	
	Winter	6.4±0.21^{defg}	6.5±0.13^{cdef}	6.7±0.05^{ab}	6.9±0.15^{a}	**6.6^{a}**	
	**Mean**	**6.2^{c}**	**6.3^{c}**	**6.6^{b}**	**6.7^{a}**		
EC	Spring	3.16±0.28^{ab}	2.12±0.23^{cd}	0.95±0.24^{ef}	0.41±0.10^{gh}	**1.66^{ab}**	Sites: 0.21<br>Seasons: 0.21<br>Sites× Seasons:0.42
	Summer	3.43±0.25^{a}	2.23±0.29^{c}	1.06±0.24^{e}	0.55±0.22^{fgh}	**1.818^{a}**	
	Autumn	2.84±0.21^{b}	1.96±0.19^{cd}	0.79±0.20^{efg}	0.35±0.13^{h}	**1.485^{bc}**	
	Winter	2.74±0.26^{b}	1.74±0.51^{d}	0.66±0.25^{efgh}	0.27±0.06^{h}	**1.35^{c}**	
	**Mean**	**3.04^{a}**	**2.01^{b}**	**0.86^{c}**	**0.40^{d}**		
**Moisture content (%)**	Spring	43.34±1.01^{b}	36.48±0.87^{d}	29.44±0.47^{g}	20.67±1.01^{j}	**32.48^{b}**	Sites:1.797<br>Seasons: 1.797<br>Sites× Seasons:3.594
	Summer	36.87±0.99^{d}	26.65±0.99^{h}	24.55±0.97^{i}	12.87±0.69^{l}	**25.23^{d}**	
	Autumn	41.43±1.01^{c}	31.37±1.03^{f}	28.09±0.99^{gh}	16.48±1.01^{k}	**29.34^{c}**	
	Winter	48.42±1.01^{a}	41.79±1.02^{bc}	33.27±1.00^{e}	24.32±1.01^{i}	**36.95^{a}**	
	**Mean**	**42.51^{a}**	**34.07^{b}**	**28.83^{c}**	**18.58^{d}**		
Standard limits (ICAR manual)	pH<br>Acidic < 6.0<br>Normal to saline (6.0-8.5)<br>Tending to became alkaline (8.9 -9.0)<br>Alkaline > 9.0		EC<br>Low <1.0 dS/m<br>Medium (1.0-3.0 dS/m)<br>High > 3.0 dS/m			Moisture content<br>Low (3- 10%)<br>Medium (20 – 40 %)<br>High > 40 %	

Mean N concentration in soils of the experimental site ranged from 110.67 to 320.49 (mg/kg). The concentration of N was maximum at center of dumping site (285.43 mg/kg), followed by inside (266.73 mg/kg), outside (241.61 mg/kg) and least (131.26 mg/kg) was reported at control site during all the seasons. Among seasons the maximum N concentration was observed during summer season (264.11 mg/kg), followed by spring season (244.93 mg/kg), autumn season (221.85 mg/kg) and least (194.14 mg/kg) was observed in winter season as depicted in Table 2. Additionally, the mean values of N between sites change significantly, and the mean values of N between all seasons were significantly different.

Soil phosphorus content at the experimental site ranged from 15.38 to 87.36 mg/kg. The highest mean P level was found at the center of the dumping site (70.07 mg/kg), followed by the inside (55.12 mg/kg) and outside (41.16 mg/kg) locations, with the lowest at the control site (22.16 mg/kg), as shown in Table 2. Seasonally, the highest mean phosphorus content was observed during the summer (60.98 mg/kg), followed by spring (49.51 mg/kg), autumn (41.85 mg/kg), and the lowest during winter (36.15 mg/kg). Additionally, there are considerable differences in the mean values of P between locations and the mean values of P throughout all seasons.

Soil potassium levels ranged from 96.54 to 301.28 mg/kg. Among seasons maximum mean potassium (239.70 mg/kg) was recorded in summer season followed by spring (222.63 mg/kg), autumn (204.14 mg/kg) and minimum (183.58 mg/kg) was recorded in winter season. Among sites mean maximum value of potassium was recorded in center (265.71 mg/kg), followed by inside (242.35 mg/kg), outside (225.85 mg/kg) and minimum (116.16 mg/kg) was recorded at control site as depicted in Table 2. Additionally, there are considerable differences in

the mean values of K between sites as well as the mean values of K across all seasons.

Table 2. Seasonal and site wise variation in N, P and K (mg/Kg) of Soil

Parameters	Seasons	Sites				Mean	C.D (P≤0.05)
		Center	Inside	Outside	Control		
N	Spring	300.33±2.43^{c}	283.40±2.74^{d}	256.73±2.06^{e}	139.27±3.13^{k}	**244.93^{b}**	Sites: 4.700<br>Seasons: 4.700<br>Sites × Seasons: 9.401
	Summer	320.49±1.80^{a}	307.15±0.65^{b}	282.34±1.95^{d}	146.47±1.16^{j}	**264.11^{a}**	
	Autumn	280.41±1.79^{d}	252.50±2.19^{f}	225.85±2.08^{h}	128.64±2.07^{l}	**221.85^{c}**	
	Winter	240.51±2.04^{g}	223.86±1.52^{h}	201.54±1.32^{i}	110.67±2.24^{m}	**194.14^{d}**	
	**Mean**	**285.43^{a}**	**266.73^{b}**	**241.61^{c}**	**131.26^{d}**		
P	Spring	71.29±1.12 ^{c}	57.42±0.89^{e}	43.95±1.71^{h}	25.39±1.08^{k}	**49.51^{b}**	Sites: 2.017<br>Seasons: 2.017<br>Sites × Seasons: 4.034
	Summer	87.36±0.85^{a}	73.44±1.87^{b}	54.68±1.24^{f}	28.45±2.01^{l}	**60.98^{a}**	
	Autumn	63.35±1.04^{d}	48.60±1.24^{g}	36.06±1.23^{j}	19.42±0.95^{m}	**41.86^{c}**	
	Winter	58.29±1.02^{e}	41.01±41.01^{i}	29.94±0.54^{k}	15.38±1.08^{n}	**36.15^{d}**	
	**Mean**	**70.07^{a}**	**55.12^{b}**	**41.16^{c}**	**22.16^{d}**		
K	Spring	275.70±0.38^{b}	251.77±0.97^{d}	237.70±1.08^{e}	125.35±1.03^{l}	**222.63^{b}**	Sites: 6.887<br>Seasons: 6.887<br>Sites × Seasons:13.774
	Summer	301.28±1.03^{a}	273.33±1.05^{c}	251.77±1.63^{d}	132.41±1.12^{k}	**239.70^{a}**	
	Autumn	253.38±1.12^{d}	234.29±0.90^{f}	218.64±1.12^{h}	110.35±0.94^{m}	**204.14^{c}**	
	Winter	232.49±1.07^{g}	210.03±1.03^{i}	195.28±0.95j	96.54±1.11^{n}	**183.58^{d}**	
	**Mean**	**265.71^{a}**	**242.35^{b}**	**225.85^{c}**	**116.16^{d}**		
Standard Limits (ICAR manual)		**N**<br>Low<107.1 mg/kg<br>Medium (107.1-214.2 mg/kg)<br>High >214.2 mg/kg		**P**<br>Low < 13.2mg/kg<br>Medium (13.2 – 33 mg/kg)<br>High > 33 mg/kg		K<br>Low < 49.10 mg/kg<br>Medium (49.10 -125 mg/kg)<br>High > 125 mg/kg	

N: Nitrogen, P: Phosphorus, K: Potassium

During the present study, mean Ca concentration ranged from 668.33 to 984.39 (mg/Kg). Mean maximum concentration of Calcium among seasons was recorded in summer season (913.52 mg/kg), followed by Spring (893.44 mg/kg), autumn (856.92) and minimum was recorded in winter (826.58). Among sites mean maxium concentration of calcium was recorded in center (957.67 mg/kg), inside (941.24 mg/kg), outside (876.38 mg/kg) and minimum (712.91 mg/kg) was recorded at control. The mean values of Ca between all seasons were significantly different; furthermore the mean values of Ca between sites also vary significantly.

During the present study the mean Mg concentration ranged from 280.82 to 418.61 (mg/Kg). Magnesium concentration was recorded maximum (385.09 mg/kg) during summer season, followed by spring (374.66 mg/kg), autumn (357.80 mg/kg) and minimum (349.58 mg/kg) was recorded in winter. Among sites maximum mean concentration of magnesium was recorded in center (402.42 mg/kg), followed by inside (396.72 mg/kg), outside (368.48 mg/kg) and minimum (299.49 mg/kg) was recorded at control site as depicted in Table 3. Additionally, there are considerable differences in the mean levels of Mg between locations as well as the mean values of Mg across all seasons.

Table 3. Seasonal and site wise variation in Ca and Mg (mg/kg) of soil

Parameters	Seasons	Sites				Mean	C.D (P≤0.05)
		Center	Inside	Outside	Control		
**Ca**	Spring	982.31±0.96^{c}	965.34±0.97^{d}	893.60±0.99^{i}	723.50±0.94^{n}	**893.44^{b}**	Sites:1.809<br>Seasons:1.809<br>Sites × Seasons:3.618
	Summer	997.74±1.13^{a}	984.39±0.90^{b}	913.43±0.93^{g}	758.51±0.88^{m}	**913.52^{a}**	
	Autumn	940.40±1.06^{e}	925.85±0.73^{f}	860.11±0.94^{l}	701.31±1.10^{o}	**856.92^{c}**	
	Winter	910.25±0.96^{h}	889.40±1.08^{j}	838.37±1.04^{k}	668.33±0.95^{p}	**826.58^{d}**	
	**Mean**	**957.67^{a}**	**941.24^{b}**	**876.38^{c}**	**712.91^{d}**		
**Mg**	Spring	412.86±0.71^{b}	405.61±1.35^{c}	376.28±0.81^{g}	303.89±1.25^{l}	**374.66^{b}**	Sites:3.873<br>Seasons:3.873<br>Sites × Seasons:7.745
	Summer	419.24±0.97^{a}	418.61±0.93^{a}	383.85±1.02^{f}	318.66±1.21^{k}	**385.09^{a}**	
	Autumn	395.12±0.77^{d}	388.99±0.90^{e}	352.50±1.27^{j}	294.61±1.13^{m}	**357.80^{c}**	
	Winter	382.47±0.95^{f}	373.67±0.94^{h}	361.38±1.03^{i}	280.82±1.17^{n}	**349.58^{d}**	
	**Mean**	**402.42^{a}**	**396.72^{b}**	**368.48^{c}**	**299.49^{d}**		
**Standard limits (ICAR manual)**		**Ca**<br>Low< 500 mg/kg<br>Medium (500-2500 mg/kg)<br>High > 2500 mg/kg			Mg<br>Sandy soils (51- 250 mg/kg)<br>Clayey soils (101-500 mg/kg)		

Ca: Calcium, Mg: Magnesium

Zinc (Zn) concentration in the soil in the current study ranged from 0.75 to 3.57 (mg/Kg). Among seasons maximum mean concentration of Zn (2.89 mg/kg) was recorded in winter season, followed by autumn (2.54 mg/kg), spring (1.75 mg/kg) and minimum was recorded in summer (1.53 mg/kg). Among sites maximum mean concentration of Zn was recorded at center (2.92 mg/kg), followed by inside (2.50 mg/kg), outside (2.03 mg/kg) and minimum (1.27 mg/kg) was recorded at control. The mean values of Zn between spring, summer and autumn were significantly different, while as no significant difference was reported between autumn and winter. However the mean values of all sites were significantly different. The mean maximum Fe in the current study ranged from 1.51 to 12.51 (mg/Kg). For iron (Fe) maximum mean concentration among seasons was recorded in winter (8.78 mg/kg), followed by autumn (8.26 mg/kg), spring (5.48 mg/kg) and minimum (4.65 mg/kg) was recorded in summer, while as among sites maximum mean concentration was recorded at center (10.56 mg/kg), followed by inside (6.52 mg/kg), outside (6.37 mg/kg) and minimum (3.71 mg/kg) was recorded at control. The mean values of Fe between all seasons were significantly different. Among sites the values of Fe were significantly different between center, inside and control, however no significant difference was reported between inside and outside dumpsite. Mean concentration of Cu ranged from 0.17 to 2.72 (mg/Kg). Copper (Cu) was recorded maximum among seasons in winter (2.11 mg/kg), followed by autumn (1.97 mg/kg), spring (1.19 mg/kg) and minimum was recorded in summer (0.88 mg/kg). Among sites maximum mean concentration of Cu was recorded in center (2.07 mg/kg), followed by inside (1.80 mg/kg), outside (1.74 mg/kg) and minimum was recorded at control (0.54 mg/kg). The mean values of Cu were significantly different throughout all seasons, and it was also observed that these differences were significant between sites.

Mean Manganese (Mn) concentration of soils ranged from 4.54 to 11.83 (mg/Kg) shown in Table 4. Manganese was recorded maximum in winter (8.92 mg/kg), followed by autumn (8.38 mg/kg), spring (7.62 mg/kg) and minimum was recorded in summer (7.24 mg/kg). Among sites maximum mean concentration was recorded at center (10.73 mg/kg), followed by inside (9.34 mg/kg), outside (7.42 mg/kg) and minimum was recorded at control (4.68 mg/kg) as depicted in Table 4. The mean Mn values also varied significantly between different locations and across all seasons.

Table 4: Seasonal and site wise variation in micronutrients, viz Zn, Fe, Cu and Mn (mg/kg) of soil

Parameters	Seasons	Sites				Mean	C.D (P≤0.05)
		Center	Inside	Outside	Control		
**Zn**	Spring	2.39±0.14^{de}	2.01±0.31^{ef}	1.55±0.33^{gh}	1.07±0.28^{ij}	**1.75^{b}**	Sites:0.212<br>Seasons:0.212<br>Sites × Seasons:0.424
	Summer	2.34±0.52^{de}	1.76±0.01^{fg}	1.30±0.04^{hi}	0.75±0.32^{j}	**1.53^{c}**	
	Autumn	3.36±0.21^{ab}	2.99±0.09^{bc}	2.53±0.17^{d}	1.27±0.12^{hi}	**2.54^{a}**	
	Winter	3.57±0.29^{a}	3.26±0.15^{ab}	2.74±0.24^{cd}	1.98±0.24^{ef}	**2.89^{a}**	
	**Mean**	**2.92^{a}**	**2.50^{b}**	**2.03^{c}**	**1.27^{d}**		
**Fe**	Spring	9.25±0.29^{b}	5.21±0.15^{f}	5.14±0.32^{f}	2.31±0.14^{h}	**5.48^{c}**	Sites:0.250<br>Seasons:0.250<br>Sites × Seasons:0.50
	Summer	8.45±0.21^{c}	4.41±0.24^{g}	4.24±0.23^{g}	1.51±0.18^{i}	**4.65^{d}**	
	Autumn	12.05±0.17^{a}	8.01±0.27^{cd}	7.85±0.11^{d}	5.13±0.19^{f}	**8.26^{b}**	
	Winter	12.51±0.89^{a}	8.46±0.15^{c}	8.27±0.18^{cd}	5.88±0.06^{e}	**8.78^{a}**	
	**Mean**	**10.56^{a}**	**6.52^{b}**	**6.37^{b}**	**3.71^{c}**		
**Cu**	Spring	1.62±0.24^{d}	1.36±0.09^{e}	1.29±0.13^{e}	0.49±0.12^{h}	**1.19^{c}**	Sites:0.117<br>Seasons:0.117<br>Sites × Seasons: 0.235
	Summer	1.33±0.11^{e}	1.04±0.08^{f}	0.99±0.11^{f}	0.17±0.06^{i}	**0.88^{d}**	
	Autumn	2.61±0.24^{ab}	2.33±0.15^{c}	2.28±0.18^{c}	0.65±0.05^{gh}	**1.97^{b}**	
	Winter	2.72±0.06^{a}	2.46±0.07^{bc}	2.39±0.19^{bc}	0.87±0.10^{fg}	**2.11^{a}**	
	**Mean**	**2.07^{a}**	**1.80^{b}**	**1.74^{b}**	**0.54^{c}**		
Mn	Spring	10.21±0.63^{cd}	8.79±0.25^{f}	6.89±0.39^{i}	4.58±0.17^{j}	**7.62^{c}**	Sites: 0.212<br>Seasons: 0.212<br>Sites × Seasons: 0.425
	Summer	9.73±0.04^{e}	8.32±0.28^{g}	6.47±0.08^{i}	4.54±0.05^{j}	**7.24^{d}**	
	Autumn	11.18±0.23^{b}	9.83±0.11d^{e}	7.81±0.39^{h}	4.69±0.06^{j}	**8.38^{b}**	
	Winter	11.83±0.06^{a}	10.42±0.09^{c}	8.53±0.21^{fg}	4.92±0.05^{j}	**8.92^{a}**	
	**Mean**	**10.73^{a}**	**9.34^{b}**	**7.42^{c}**	**4.68^{d}**		
Standard limits (ICAR manual)	**Zn**<br>Low < 0.5 mg/kg<br>Medium (1 – 3 mg/kg)<br>High > 5 mg/kg		**Fe**<br>Low < 2 mg/kg<br>Medium (4 -6mg/kg)<br>High > 10 mg/kg	**Cu**<br>Low < 0.1 mg/kg<br>Medium (0.3 -0.8mg/kg)<br>High > 3 mg/kg		Mn<br>Low< 0.5 mg/kg<br>Medium (1.2 -3.5 mg/kg)<br>High > 6 mg/kg	

Zn: Zinc, Fe: Iron, Cu: Copper, Mn: Manganese

In the present study the concentration of lead (Pb) ranged from 0.98mg/kg to 98.3 mg/kg. The mean maximum concentration of lead (Pb) among seasons was recorded in summer (72.1 mg/kg), followed by autumn (68.5 mg/kg), spring (59.08 mg/kg) and minimum was recorded in winter (35.9 mg/kg) while as among sites maximum mean concentration of Pb was recorded at center (85.02 mg/kg), followed by inside (76.02 mg/kg), outside (68.72 mg/kg) and minimum (5.90 mg/kg) was recorded at control. Additionally to the fact that the mean Pb values between sites varied greatly, these mean values of Pb between seasons varied significantly as well. The concentration of cadmium Cd ranged from 0.06mg/kg to 5.89 mg/kg. Cadmium (Cd) was recorded maximum in summer season (4.46 mg/kg) , followed by autumn (3.80 mg/kg), spring (3.22 mg/kg) and minimum (1.57 mg/kg) was recorded in winter and among maximum mean concentration of Cd was recorded at center (4.56 mg/kg), followed by inside (4.26 mg/kg), outside (4.08 mg/kg) and minimum was recorded at control (0.15 mg/kg). Among the sites, the mean cadmium values were significantly different between the center and control, and inside and control; however, no significant difference was reported between outside and control. The concentration of Nickel (Ni) ranged from 0.65mg/kg to 42.10 mg/kg. Ni was recorded maximum in summer (29.70 mg/kg), followed by autumn (22.23 mg/kg), spring (17.01 mg/kg) and minimum was recorded in winter (9.30 mg/kg). In case of sites it was recorded maximum at center (29.04 mg/kg), followed by inside (25.86 mg/kg), outside (21.88 mg/kg) and minimum (1.47 mg/kg) was recorded at control as depicted in Table 5. The mean values of Ni between seasons as well as sites differ significantly. The concentration of chromium (Cr) ranged from 2.31 mg/kg to 30.40 mg/kg. Among seasons max concentration (22.41 mg/kg) was

reported during summer season followed by autumn (19.67mg/kg), spring (15.37 mg/kg) and min during winter (9.88 mg/kg). Among sites maximum concentration (23.37 mg/kg) was reported at center of dumpsite, followed by inside (21.12 mg/kg), outside (18.61 mg/kg) and minimum was reported at control site (4.23mg/kg). The mean values of Cr between seasons were reported to be significantly different. Among sites the mean values of Cr between inside, outside, control were significantly different; however no significant difference in mean values of Cr was reported between center and inside. The value of Arsenic (As) ranged from 0.23 mg/kg to 18.50 mg/kg. Among seasons max concentration (12.63 mg/kg) was reported during summer season followed by autumn (9.86 mg/kg), spring (8.04 mg/kg) and min during winter (5.63 mg/kg). Among sites maximum concentration (14.10 mg/kg) was reported at center of dumpsite, followed by inside (11.53 mg/kg), outside (9.87 mg/kg) and minimum was reported at control site (0.66mg/kg) as depicted in Table 6. The mean values of As between seasons were significantly different. In addition to this the mean values of As between all sites was also reported to be significantly different. Furthermore the correlation between the recorded paramaters is depicted in Table 7.

Table 5. Seasonal and site wise variation in Pb, Cd and Ni (mg/Kg) of soil

Parameters	Seasons	Sites				Mean	C.D (P≤0.05)
		Center	Inside	Outside	Control		
Pb	Spring	89.1±3.34^{b}	76.4±1.62^{c}	65.8±2.44^{d}	5.03±0.58^{hi}	**59.08^{c}**	Sites: 1.799<br>Seasons: 1.799<br>Sites × Seasons: 3.557
	Summer	98.3±4.05^{a}	92.5±2.53^{ab}	87.4±2.25^{b}	10.2±1.22^{h}	**72.1^{a}**	
	Autumn	96.8±1.69^{a}	88.6±2.09^{b}	81.4±1.56^{c}	7.4±0.90^{h}	**68.5^{b}**	
	Winter	55.9±1.41^{e}	46.6±2.00^{f}	40.3±1.83^{g}	0.98±0.07^{i}	**35.9^{d}**	
	**Mean**	**85.02^{a}**	**76.02^{b}**	**68.72^{c}**	**5.90^{d}**		
Cd	Spring	4.69±1.08^{a}	4.12±1.00^{ab}	3.98±0.85^{ab}	0.12±0.02^{cd}	**3.22^{b}**	Sites: 1.10<br>Seasons:1.10<br>Sites × Seasons: 2.20
	Summer	6.11±1.28^{a}	5.89±0.76^{a}	5.61±0.66^{a}	0.25±0.08^{cd}	**4.46^{a}**	
	Autumn	5.11±1.23^{a}	5.01±0.50^{a}	4.89±1.28^{a}	0.19±0.03^{cd}	**3.80^{ab}**	
	Winter	2.34±0.57^{bc}	2.02±0.43^{bcd}	1.87±0.52^{bcd}	0.06±0.32^{d}	**1.57^{c}**	
	**Mean**	**4.56^{a}**	**4.26^{a}**	**4.08 ^{a}**	**0.15^{b}**		
Ni	Spring	26.20±1.02^{de}	22.50±2.15^{ef}	18.30±1.45^{fg}	1.06±0.24^{i}	**17.01^{c}**	Sites: 2.047<br>Seasons: 2.047<br>Sites × Seasons:4.093
	Summer	42.10±3.02^{a}	38.70±4.33^{ab}	35.70±2.80^{bc}	2.32±0.46^{i}	**29.70^{a}**	
	Autumn	32.40±2.11^{c}	29.80±3.51^{cd}	24.90±1.96^{de}	1.85±0.19^{i}	**22.23^{b}**	
	Winter	15.47±2.17^{g}	12.46±1.64^{gh}	8.65±1.10^{h}	0.65±0.18^{i}	**9.30^{d}**	
	**Mean**	**29.04^{a}**	**25.86^{b}**	**21.88^{c}**	**1.47^{d}**		

Permissible value of soil (mg/kg)	Permissible value of plant	Permissible value of water
Pb=85; Cd=0.8 ; Ni=35	Pb=2; Cd=0.02 ; Ni=10	Pb=0.05; Cd=0.005 ; Ni=0.07

Pb: Lead, Cd: Cadmium, Ni: Nickel

Table 6. Seasonal and site wise variation in Cr, and As (mg/Kg) of soil

Parameters	Seasons	Sites				Mean	C.D (P≤0.05)
		Center	Inside	Outside	Control		
Cr	Spring	21.80±1.52[cd]	19.60±1.62[de]	16.70±1.49[ef]	3.41±0.63[i]	**15.37[c]**	Sites: 1.43<br>Seasons: 1.43<br>Sites × Seasons: 2.86
	Summer	30.40±3.25[a]	27.60±1.58[ab]	25.30±2.01[bc]	6.34±0.72[hi]	**22.41[a]**	
	Autumn	26.10±1.21[abc]	24.90±1.95[bc]	22.80±2.52[cd]	4.89±1.03[i]	**19.67[b]**	
	Winter	15.20±1.47[ef]	12.40±1.54[fg]	9.64±0.41[gh]	2.31±0.26[i]	**9.88[d]**	
	**Mean**	**23.37[a]**	**21.12[a]**	**18.61[b]**	**4.23[c]**		
As	Spring	13.40±1.12[bcd]	9.65±0.65[ef]	8.67±0.42[ef]	0.45±0.07[h]	**8.04[c]**	Sites: 1.31<br>Seasons: 1.31<br>Sites × Seasons:2.62
	Summer	18.50±1.47[a]	16.30±1.76[ab]	14.60±1.45[bc]	1.12±0.12[h]	**12.63[a]**	
	Autumn	15.30±1.58[bc]	12.70±1.26[cd]	10.60±1.15[de]	0.86±0.05[h]	**9.86[b]**	
	Winter	9.21±0.79[ef]	7.48±0.68[fg]	5.62±1.10[g]	0.23±0.07[h]	**5.63 [d]**	
	**Mean**	**14.10[a]**	**11.53[b]**	**9.87[c]**	**0.66[d]**		
Permissible value of soil (mg/kg)<br>Cr= 100 ;<br>As=20				Permissible value of plant (mg/kg)<br>Cr= 1.30<br>As=0.05		Permissible value of Water (mg/kg)<br>Cr: 0.05<br>As: 0.05	

Cr: Chromium, As: Arsenic

Table 7: Correlation between various physico-chemical paramaters of soil

	pH	EC	MC	N	P	K	Ca	Mg	Zn	Fe	Cu	Mn	Pb	Cd	Ni	Cr	As
pH	1																
EC	-0.86	1															
MC	-0.48	0.78	1														
N	-0.87	0.81	0.61	1													
P	-0.92	0.92	0.60	0.93	1												
K	-0.86	0.82	0.65	0.99	0.92	1											
Ca	-0.88	0.84	0.67	0.98	0.92	0.98	1										
Mg	-0.87	0.84	0.67	0.98	0.92	0.98	0.99	1									
Zn	-0.36	0.57	0.85	0.37	0.34	0.43	0.45	0.45	1								
Fe	-0.39	0.64	0.86	0.40	0.41	0.46	0.45	0.45	0.93	1							
Cu	-0.33	0.46	0.80	0.39	0.28	0.46	0.47	0.46	0.95	0.88	1						
Mn	-0.68	0.84	0.93	0.71	0.69	0.75	0.78	0.78	0.88	0.86	0.85	1					
Pb	-0.87	0.75	0.56	0.95	0.86	0.95	0.93	0.92	0.43	0.46	0.48	0.70	1				
Cd	-0.84	0.67	0.43	0.93	0.83	0.92	0.89	0.87	0.31	0.33	0.36	0.57	0.98	1			
Ni	-0.89	0.71	0.41	0.92	0.88	0.91	0.87	0.86	0.29	0.33	0.30	0.57	0.95	0.98	1		
Cr	-0.90	0.73	0.45	0.93	0.87	0.92	0.90	0.88	0.35	0.38	0.38	0.62	0.98	0.98	0.98	1	
As	-0.90	0.79	0.54	0.95	0.91	0.95	0.92	0.91	0.41	0.45	0.42	0.69	0.97	0.96	0.98	0.98	1

Discussion

Soil pH determines the acidity or alkalinity of soil solutions and refers to the soil's hydrogen ion content. Natural soil typically has a pH between 7 and 8.5, although this range can vary based on biological activity, temperature, and municipal waste disposal practices [28]. Soil pH influences the availability of essential plant nutrients and the concentration of toxic elements, thereby affecting plant growth [29]. As it impacts the availability of all nutrients in the soil it has a direct effect on the survival and development of plants. In this, study pH of dumpsite soils ranged from 6.1 to 6.9 throughout study period. The mean pH values ranged from slightly acidic to neutral, which may be due to the decomposition of organic matter leading to the formation of organic acids. However, the pH increased with increase in distance from dumpsite which may be attributed to release of less organic acids away from dumpsite. Further, the lowest pH value reported during summer season may be attributed to more degradation of organic matter leading to increase in production of organic acids because of increased microbial activity and favorable temperature and moisture conditions during summer season as compared to winter season as depicted in Fig 2.

Fig 2: Graphical representation of pH among different sites and seasons. S1 is spring, S2 is summer, S3 is autumn and S4 is winter, while as C1 is center, C2 is inside, C3 is outside and C4 is Control.

The pH value of control site (Shalimar) soil was higher than the other three sites during all the seasons, this may be due to presence of less organic matter and hence less microbial activity. The slightly acidic pH of the soils at three other sites suggests that solid waste contributed to soil

acidity, leading to a decrease in the availability of essential plant nutrients such as phosphorus and molybdenum, while increasing the availability of toxic elements, particularly aluminum and manganese [30]. Electrical conductivity is an indirect measurement that is correlated with a number of the physical and chemical characteristics of soil. EC is affected by moisture content that soil particles hold. The total quantity of anions and cations determines electrical conductivity. It also depends on the primary salts in the soil solution, which have a negative impact on the qualities of the soil [31]. The optimal EC is crop specific, and depends on environmental conditions [32]. In general, higher EC hinders nutrient uptake by increasing the osmotic pressure of the nutrient solution, and the increased discharged of nutrients into the environment, resulting in environmental pollution. Lower EC may severely affect plant health and yield [33, 34]. In the current study, maximum mean value of EC was reported at center of dumping site, which may be due to increased concentration of cations and anions within the dumpsite soil [35] and minimum was reported at control site. Among seasons maximum mean EC value was reported during summer season, which may be due to increase in degradation of salts and release of large number of cations and anions favoured by temperature and minimum was reported during winter due to decrease in degradation of organic matter as shown in Table 1 and Fig 3.

Fig 3: Graphical representation of EC (dS/m) among different sites and seasons. S1 is spring, S2 is summer, S3 is autumn and S4 is winter, while as C1 is center, C2 is inside, C3 is outside and C4 is Control

The electrical conductivity of the soil samples collected within the dumpsite was high compared to the soil sample collected from the control site. Moisture content refers to the amount of water

soil can hold. Most soil samples from municipal solid waste have moisture content ranging from 15% to 40% [31]. The highest mean moisture content was observed at the center of the dumping site, which may be because dumpsite soil retains more moisture due to the natural ground being covered by municipal solid waste, which has high moisture content and prevents soil moisture from evaporating directly [36]. The mean moisture content of soil decreased with the increase of the distance from the dumpsite. This higher moisture content in the dumpsite soil may be associated with the presence of high organic matter which has capacity to hold more water [37]. This excess amount of water suffocates plant roots by reducing the amount of oxygen available to them. Without enough oxygen, roots are not able to absorb nutrients and water effectively, which in turn inhibits the growth of plants. The moisture content was high during winter season which may be attributed to the high precipitation rate during winter. Nitrogen (N) is the most important ingredient that plants take up from the soil. In the present study, the concentration of N was maximum at center of dumping site during all the seasons as shown in Table 2. The samples within the dumpsite have high N concentration than the samples away from dumpsite, which may be attributed to abundant amount of the putrescible waste (biowaste, kitchen waste) [38]. The maximum N concentration observed during summer season may be due to increase in reaction rate because of high temperature. Additionally, the high levels of organic matter present in dump soil may be responsible for the high N content [39]. It has been reported that application of N significantly increases tuber yield and other yielding components in Colocasia [40]. Although N is a necessary component for the synthesis of proteins and amino acids, a high concentration of nitrogen in the tissues can be hazardous to the plant's physiological and phonological responses [41]. The movement of leachate from municipal solid waste (MSW) disposal site can increase the phosphorus (P) content in soil at dumpsites. In the current study the

highest mean P level was found at the center of the dumping site. e which may be attributed to the large quantities of organic waste, paper and cardboard, textiles, and plastics [42]. Seasonally, the highest mean phosphorus content was observed during the summer (and the lowest during winter. The concentration of P was higher in the soil samples of the dumping site than the controlled site. This may be explained by the fact that organic matter in waste contains sizable amounts of P, and that orthophosphate is released into the soil solution during mineralization. Additionally, humic substances and organic acids can be absorbed into soil surfaces, leading to depletion in ability of P adsorption by obstructing sites for the formation of complexes with Al, Fe, and Ca. Inorganic and organic products are also produced during the partial decomposition of organic waste [43]. Potassium readily dissolves in water; however, it remains largely intact in undisturbed soils. After being released from decomposing organic matter, potassium quickly and strongly binds to clay particles [31]. In this study the highest concentration of potassium was found at the center of the dumping site, likely due to the decomposition of a substantial amount of kitchen waste. Conversely, the lowest concentration was observed at the control site, where there was less organic waste as given in Table 2. Among seasons, maximum K content reported during summer may be due to high decomposition rate of kitchen waste and less leaching and minimum content of it reported during winter may be due to high leaching losses by precipitation. Similar results were obtained by Deshmukh and Aher [28]. Despite the fact that K controls stomatal opening and shutting, cell enlargement, and other critical physiological activities. Numerous research have examined importance of potassium on plant growth, however high K concentrations in soil solutions restrict magnesium uptake, leading to magnesium deficiency in plants [44]. Calcium (Ca) is crucial element in figuring out the soil's physical and chemical properties, especially its structure and pH. Clay platelets in soil colloids (caused by

calcium ions) bind or aggregate to produce crumbs or peds. Frequent correlation has been reported between pH and exchangeable calcium levels in soil. For example in the pH range of 7.0 to 8.5, Ca is accessible, while as low Exchangeable calcium levels were reported in acidic pH [31]. In the current study maximum Ca content reported during summer may be because of degradation of calcium rich waste such as bones and eggshells present in the kitchen waste and minimum was reported during winter, which may be because of low rate of degradation of waste. Among sites, maximum Ca content reported at center of dumping site may be attributed to the clayey nature of soils of dumpsite as shown in Table 3. Magnesium (Mg) is found in both relatively soluble forms and ionic form, Mg^{2+}, which is attached to the soil colloidal complex. It has been reported that magnesium levels in acidic soils, especially sandy soils, are frequently low, while as neutral soils usually contain more exchangeable magnesium [31]. In this investigation among sites maximum Mg concentration reported at center of dumping site may be because of its less mobility due to presence of high organic waste and clayey nature of soil [45] and minimum at control site may be because of the presence of less organic matter as given in Table 3. Among seasons maximum Mg concentration reported during summer season may be beca use of less leaching and high rate of decomposition of organic waste and minimum during winter may be attributed to high leaching loss due to the high positive water balance [45]. An excessive amount of magnesium in the soil inhibits plant growth because it reduces the ability of roots to reduce TTC (2, 3, 5-Triphenyl Tetrazolium chloride) through the inhibition of the dehydrogenase enzyme linked to the respiratory systems [46].

Micronutrients are essential for sustaining the fertility of the soil and crop productivity. Compared to macronutrients, micronutrients are required in smaller amounts. Even if the supply of macronutrients is balanced and high yielding varieties are cultivated, lack of micronutrients

will prevent maximum yield from being achieved [47]. In this study, the presence of Zn element in both naturally occurring materials, such as yard wastes and food wastes, as well as man-made materials like pigments, inks, metals, and plastics may account for the highest mean Zn value at the centre of the dumping site, while the lowest mean Zn value was reported at the control site, as shown in Table 4. Furthermore micronutrients are more tightly bonded to the soil at high pH than at low pH, the highest mean Zn concentration during the winter season may be attributed to low pH, whereas the lowest mean Zn concentration during the summer season may be attributed to high pH [48]. Although Zn serves as a nutrient for plants, but is hazardous to them in larger amounts. Zn toxicity in plants results in growth inhibition, a reduction in biomass production, competition for nutrients, inactivation of enzymes, removal of vital components from functional locations, chlorosis, and, in certain situations, blockage of Fe translocation [49].

Ferric oxide (Fe_2O_3), also known as hematite, is the most prevalent form of Iron (Fe) in soils. It is exceedingly insoluble and gives soil its red colour. Commonly, the oxide form is hydrated [50]. In this study the maximum mean Fe concentration recorded at the centre of the dumping site may be attributed to the presence of organic matter in soils, as organic matter rich soils contain Fe in the reduced state (Fe^{2+}) or is adsorbed on the surface of soil particle [50]. The maximum iron concentration reported during winter season depicted in Table 4 may be attributed to low pH, because with increase in pH, ferrous ion (Fe^{2+}) gets converted to ferric ion (Fe^{3+}). Low solubility in solution makes the ferric ion (Fe^{3+}) compounds less bioavailable, and a minimum concentration was recorded during the summer [51]. The root cells and plasma membrane are damaged by the extra iron ions, which results in oxidative stress and increased formation of reactive oxygen species (ROS). Root cells may die as a result of ROS harming cellular components such lipids, proteins, and

DNA [52]. Furthermore it, can also affect the uptake of other essential elements, such as phosphorus and zinc, by competing with the transporters that facilitate their uptake by the roots. This interference can lead to nutrient imbalances, reduced growth, and overall plant health [53].

Compared to other trace metals, copper (Cu) has extremely little mobility because it is firmly adsorbed onto soil particles. Due to this reduced mobility, Cu has a tendency to concentrate on soil particles. Leaching of Cu occurs when the amount of Cu in the soil exceeds the capacity of the soil type to hold the copper ions. The pH, cation exchange capacity, organic matter content, and presence of iron, manganese, and aluminium oxides all affect how much Cu is present in soils. The amount of water in the soil affects the ability of the soil to store copper through biotic and abiotic oxidation-reduction reactions [31]. Among sites mean maximum concentration reported at center of dumping site may be due to the higher stability constants of Cu complexes with organic matter and minimum was reported at control site as shown in Table 4. Among seasons, maximum Cu concentration reported during winter season may be because of low pH, as at low pH Cu compounds are more soluble and minimum concentration of Cu reported during summer may be because of high pH, as high pH results in precipitation of copper (II) hydroxide ($Cu(OH)_2$) and Copper(I) hydroxide (CuOH) [54]. All phases of plant growth are affected by high Cu concentration toxicity, which has considerable detrimental impacts on morphology, physiological function, and molecular level [55]. Additionally, too much copper in the soil inhibits the activity of -amylase and invertase isoenzymes, which limits the ability of many plant species to break down reserve food supplies like starch and sucrose [56]. Maximum concentration of Mn reported at center of dumping site may be due to decomposition of plants and animals waste

as well as animal excrement [57]. The lowest concentration of Mn was reported at control site as given in Table 4. The highest Mn concentration observed during the winter season, is likely due to a decrease in soil pH. At low pH, trace elements are typically more soluble because of high desorption and low adsorption [31]. Conversely, the lowest Mn concentration was reported during the summer, which may be attributed to increased adsorption [58]. Manganese is essential for terrestrial plants, but excessive accumulation in leaves can cause toxicity and reduce crop yield.

Heavy metals are significant pollutants that enter in to environment through various channels, including landfill leachate, solid waste, and wastewater. According to Radfard et al. [59] and Kusin et al. [60], heavy metals in leachate contaminate soil and subsurface water supplies. As these contaminants move up the food chain, they impact human health and cause various ailments. Leachate is a critical source of pollution, releasing chemicals into soil and water sources, and its toxicity to human cells highlights its environmental impact [61]. In the current study among seasons max mean concentration of Pb was reported during summer season and min during winter. Among sites maximum concentration was reported at center of dumpsite and minimum was reported at control site. The values of Pb are higher than permissible limit (85mg/kg) at center during spring, summer and autumn, inside during summer and autumn and outside during summer season as depicted in Table 5 and Fig 4.

Fig 4: Graphical representation of Pb (mg/kg) among different sites and seasons. S1 is spring, S2 is summer, S3 is autumn and S4 is winter, while as C1 is center, C2 is inside, C3 is outside and C4 is Control.

Lead-acid batteries used in automobiles, rechargeable nickel-cadmium batteries, consumer electronics, glass, ceramics, and plastic products including PVC resins are sources of Pb in

landfill leachate. Other sources of lead include soldered cans, pigments, brass and bronze products, rubber goods, spent motor oil, and wine bottle wrappers made of lead foil. Among seasons max concentration of Cd was reported during summer season and min during winter. Among sites maximum concentration was reported at center of dumpsite, and minimum was reported at control site. Cadmium (Cd) levels at all sites exceeded the permissible limit of 0.8 mg/kg across all seasons as depicted in Table 5 and Fig 5.

Fig 5. Graphical representation of Cd (mg/kg) among different sites and seasons. S1 is spring, S2 is summer, S3 is autumn and S4 is winter, while as C1 is center, C2 is inside, C3 is outside and C4 is Control.

The sources of cadmium in waste include nickel-cadmium batteries, pigments, stabilizers in plastics (primarily PVC), consumer electronics (notably the steel chassis of vintage TVs and radios that were cadmium-plated to prevent corrosion), antique appliances with cadmium-plated parts, textile dyes and paints, glass and ceramics, and pigments used in non-newspaper printing inks. Cadmium is one of the most ecotoxic metals, causing detrimental effects on soil health, plant metabolism, and the health of humans and animals [62]. Even at very low concentrations, chronic exposure to cadmium can lead to anemia, anosmia, cardiovascular diseases, and renal problems [63]. Ni was reported maximum during summer season and min during winter. Among sites maximum concentration was reported at center of dumpsite and minimum was reported at control site as depicted in Table 5 and Fig 6.

Fig 6. Graphical representation of Ni (mg/kg) among different sites and seasons. S1 is spring, S2 is summer, S3 is autumn and S4 is winter, while as C1 is center, C2 is inside, C3 is outside and C4 is Control.

The values of Ni were higher than the permissible limits (35 mg/kg) during summer season at all sites. The values were lower than the permissible limits during other seasons. The sources of Ni are lead cadmium batteries, electroplating materials, clinical wastes [64]. Because nickel is non-biodegradable and has the potential to accumulate in the food web, it is responsible for a number of health issues in humans, including allergic skin, headache, vertigo, nausea, vomiting, insomnia, and irritability [64, 65]. Among seasons max concentration of Cr was reported during summer season and min during winter. Among sites maximum concentration was reported at center of dumpsite, and minimum was reported at control site as depicted in Table 6 and Fig 7.

Fig 7. Graphical representation of Cr (mg/kg) among different sites and seasons. S1 is spring, S2 is summer, S3 is autumn and S4 is winter, while as C1 is center, C2 is inside, C3 is outside and C4 is Control.

The sources of Cr are electroplating and Cr containing wastes. The concentration of Cr was less than permissible value at all sites. The sorption properties of the soil, such as its clay, iron oxide, and organic matter contents, influence the mobility of Cr. Surface runoff can carry chromium to surface waters in both its soluble and precipitated forms. Chromium compounds, both soluble and unadsorbable, can seep into groundwater from the soil. As soil pH rises, Cr (VI) has a greater propensity to leach. However, the majority of Cr discharged into natural rivers is particle associated and eventually deposits into the sediment [66]. Human allergic dermatitis and chromium are related [67]. The value of Arsenic (As) ranged from 0.23 mg/kg to 18.50 mg/kg. Among seasons max concentration was reported during summer season and min during winter. Among sites maximum concentration was reported at center of dumpsite, and minimum was

reported at control site as depicted in Table 6 and Fig 8.

Fig 8. Graphical representation of As (mg/kg) among different sites and seasons. S1 is spring, S2 is summer, S3 is autumn and S4 is winter, while as C1 is center, C2 is inside, C3 is outside and C4 is Control.

The permissible limit of As in soil is 20 mg/kg. The concentration of heavy metals, specifically Pb, Cd, Ni, Cr, and As, is higher during the summer than other seasons. The leaching of heavy metals is also higher during the summer because temperature influences it; as the temperature rises, the leaching of heavy metals also increases. However, the minimum concentration of heavy metals during winter season might be attributed to runoff effect during winter season which facilitates the leaching of heavy metals from soil and contributes to the dilution of soil solution during winter season.

Heavy metal toxicity in plants severely hampers nutrient and water uptake, while intensifying oxidative damage, ultimately stunting plant growth. Cadmium (Cd) has been shown to interact with plants at the physio-biochemical level, leading to reduced growth. Cd toxicity disrupts nutrient and water absorption, heightens oxidative stress, and impairs plant metabolism, and negatively impacts plant morphology and physiology [68]. Similarly, chromium (Cr) toxicity inhibits seed germination, slows plant growth, impairs enzymatic activities, and disrupts the photosynthetic machinery, leading to oxidative imbalances [69, 70]. Lead (Pb) toxicity has been found to inhibit ATP production, elevate reactive oxygen species (ROS) levels, and cause DNA damage. It significantly affects germination, root elongation, plant growth, chlorophyll synthesis, and disturbs transcriptome sequencing [71]. Nickel (Ni) toxicity reduces seed germination, root and shoot development, biomass accumulation, and overall yield. It also causes chlorosis,

necrosis, and disrupts physiological processes like photosynthesis and transpiration, while inducing oxidative stress. The threat posed by Ni increases with its concentration in the environment, particularly in soils [72]. Arsenic (As) exposure adversely affects plants at both biochemical and molecular levels, inhibiting key physiological processes such as overall growth, photosynthetic efficiency, and biomass production. Arsenic induces oxidative stress by increasing ROS production or reducing their elimination, leading to damage of lipids, proteins, and nucleic acids. It interferes with metabolic pathways, either by acting as a competitive inhibitor of phosphate (Pi) or by disrupting enzyme activities. Additionally, As inhibits seed germination, root and shoot development, and other critical early-stage processes during seedling growth [73].

Available management strategy for remediation of soil health:

Soil is very important asset for plant and human health, so the need of hour is to maintain the soil quality and prevent its degradation. The waste generated from various sources like houses, restaurants, agricultural fields, orchards is mostly organic (65-75%), which is diverted to landfill in huge amounts on daily basis can be segregated at source and then converted into compost. The compost generated can then be used to ameliorate the nutrient status of soil quality as well as to enhance the plant growth and development. Furthermore the compost generated will replace the chemical fertilizers used. The plan of utilization of microbial consortium technology for degradation of waste is depicted in flow chart given in Fig 9.

Fig 9: Flow chat depicting management strategy for waste

Conclusion:

The soil plays a crucial role in controlling the flow of pollutants into the food chain. Population growth is causing waste generation to grow rapidly as well. Landfills have been a popular choice for residue dumping for the past 20 years. Our investigation has shown that, regardless of the time of year or the distance from the waste site, landfills seriously degrade the soil quality of nearby communities. pH was more acidic during the summer season and at center of the dumping site due to an increase in the production of organic acids, while as it was less acidic during the winter season and away from the dump site. The acidic pH results in a decrease in the availability of essential elements viz, N.P and K, however, it results in an increase in the availability of toxic heavy metals viz, Pb, Cd, Ni, Cr, As. The heavy metals were reported to be maximum during the summer season at all sites and minimum during the winter season. Among sites the heavy metals were maximum and decreased with increase in distance from the landfill. Our findings led to the conclusion that there is a need for global attention on improving or restoring soil health. Assessment of soil health indicators is expected to enhance our understanding of the factors underlying processes that contribute to sustainable agriculture. The leachate must be treated before disposal and that landfills must have adequate lining. Before disposing of rubbish, segregate non-biodegradable items like polythene and plastic bags since they take roughly 500 years to degrade. Biodegradable garbage must be handled using ecologically friendly techniques like anaerobic digestion and composting. The garbage that needs to be landfilled should only be non-biodegradable, inert, or unsuitable for recycling. MSW must not be combined with biomedical waste. Media should be used to inform people about trash management. The organic waste ought to be isolated at the point of generation and turned into compost using microbial processes.

Acknowledgement:

The authors acknowledge the support of SKUAST Kashmir and Division of Environmental sciences in carrying out the research.

Author contribution: Shayesta Islam: methodology, writing; statistical analysis; Haleema Bano: methodology; Malik Ahsaf Aziz: review and editing; Fahad Naseer Alotaibi: Review and Editing.

References:

1. IPBES (2018): The IPBES assessment report on land degradation and restoration. Montanarella, L., Scholes, R., and Brainich, A. (eds.). Secretariat of the Intergovernmental Science-Policy Platform on Biodiversity and Ecosystem Services, Bonn, Germany. 744.doi:https://doi.org/10.5281/zenodo.3237392"

2. Stoessel F, Sonderegger T, Bayer P, Hellweg S. Assessing the Environmental Impacts of Soil Compaction in Life Cycle Assessment. The Science of the Total Environment. 2018 Feb ; 630: 913−921. doi: https://doi.org/10.1016/j.scitotenv.2018.02.222"

3. Brevik EC, Sauer TJ. The past, present, and future of soils and human health studies. Soil. 2015 Jan 06; 1: 35–46. doi: https://doi.org/10.5194/soil-1-35-2015"

4. Nielsen UN, Wall DH, Six J. Soil biodiversity and the environment. Annual Review of Environment and Resources. 2015 Nov 11; 40: 63–90. doi:http: //dx.doi.org /10.1146/ annurev-environ-102014-021257"

5. Horton RE. The role of infiltration in the hydrologic cycle. Eos, Transactions of the American Geophysical Union. 1993 Jan 01; 14: 446–460. doi:http:// dx.doi.org /10.1029/ TR014i001p00446"

6. Ahmad R, Gao J, Li W, Yuanyuan Z, Gao Z, Khan A, et al. Response of soil nutrients, enzyme activities, and fungal communities to biochar availability in the rhizosphere of mountainous apple trees. Plant Soil. 2023 April 25. 489:277–293. doi: https://doi.org/10.1007/s11104-023-06016-4

7. Kaza S, Yao LC, Bhada-Tata P, Van Woerden F. What a Waste 2.0 : A Global Snapshot of Solid Waste Management to 2050. Urban Development. Washington, DC: World Bank. 2018 sept 20. Available from: https://hdl.handle.net/10986/30317.

8. Bhat R.A., Dervash M.A., Mehmood M.A., Hakeem, K.R. Municipal Solid Waste Generation and its Management, a Growing Threat to Fragile Ecosystem in Kashmir Himalaya. American Journal of Environmental sciences. 2017; 13: 388-397. doi:https://doi.org/10.3844/ajessp.2017.388.397"

9. Wani MA, Ahmad S. Challenges, issues of solid waste management in himalayas: A case study of srinagar city. African Journal of Basic and Applied Sciences. 2013; 5(1): 25-29. doi:https://api.semanticscholar.org/CorpusID:110732616"

10. Nazir MI, Sundan S, Fayaz S, Dar MA, Raju JL. The effect of dumping site leachate on ground water quality: A case study of Srinagarcity, India. International Journal of Civil, Structural, Environmental and Infrastructure Engineering Research and Development. 2014; 4(1): 1-8. https://api.semanticscholar.org/CorpusID:201821214"

11. Naveen B P, Mahapatra DM, Sitharam T G, SivapullaiahPV, Ramachandra TV. Physico-chemical and biological characterization of urban municipal landfill leachate. Environmental Pollution. 2017 Jan; 220: 1–12. doi:http:// dx.doi.org/ 10.1016/ j. envpol.2016.09.002”

12. Arunbabu V, Indu K S, Ramasamy EV. Leachate pollution index as an effective tool in determining the phytotoxicity of municipal solid waste leachate. Waste Management . 2017Jul 21; 68: 329–336. doi:10.1016/j.wasman.2017.07.012”

13. Bolan N, Kunhikrishnan A, Thangarajan R, Kumpiene J, Park J, Makino T, et al. Remediation of heavy metal(loid)s contaminated soils – To mobilize or to immobilize. Journal of Hazardous Materials. 2014 Feb 15; 226: 141-166. doi:10.1016/ j.jhazmat. 2013.12.018”

14. Sweety P. Impacts of Soil Pollution on Environment. Natural Volatiles and Essential Oils. 2021; 8(5): 3314-3320.

15. Ngatia L, Johnny M, Grace III, Moriasi D, Taylor R. Nitrogen and Phosphorus Eutrophication in Marine Ecosystems. In: Fouzia HB, editor. Monitoring of Marine Pollution. London, England: Intech open; 2019.pp. 1-17. doi:10.5772/intechopen.81869”

16. Jahany M, Rezapour S. Assessment of the quality indices of soils irrigated with treated wastewater in a calcareous semi-arid environment. Ecological Indicators. 2020 Feb; 109:105800. doi:https://doi.org/10.1016/j.ecolind.2019.105800”

17. Rezapour S, Samadi A, Kalavrouziotis IK, Ghaemian N. Impact of the uncontrolled leakage of leachate from a municipal solid waste landfill on soil in a cultivated-calcareous

environment. Waste Management. 2018 Dec; 82:51–61. doi:10.1016/j.wasman.2018.10.013”

18. Zaman MU, Pandit BA, Kumar R, Khan S. Challenges, Issues and Opportunities towards Management of Solid wastes in Indian Cities: A Case Study of Srinagar City. International Journal for Research in Applied Science & Engineering Technology. 2022 Dec; 10(12). doi:10.22214/ijraset.2022.48196”

19. Khan NA. Perspectives of Transport and Disposal of Municipal Solid Waste in Srinagar City. International Journal of Engineering Research and General Science. 2014 Jul; 2(4): 238-251. www.ijergs.org”

20. Prihar SS, Sandhu BS. A rapid method for soil moisture determination. Soil Science. 1968; 105(3): 142-144.

21. Jackson ML. Soil chemical analysis. New Delhi: Prentice hall of India Private limited; 1973.

22. Subbaih BV, Asija GL. A rapid procedure for the estimation of available nitrogen in the soil. Current Science Journal. 1956; 25: 259-266.

23. Olsen SR, Cole C V, Watanabe FS, Dean LA. Estimation of available phosphorous in soils by extraction with sodium bicarbonate. Circular of the United States Department of Agriculture. Washington. D. C. 939. 1954.

24. Chesin L, Yein CH. Turbid metric determination of available sulphur. Soil Science Society of America Proceedings. 1951; 15: 149-151.

25. Lindsay WL, Norvell WA. Development of a DTPA-soil test for Zn, Fe, Mn and Cu. Soil Science Society of America Journal. 1978; 42(3): 421-428.

26. Gomez KA, Gomez AA. Statistical Procedures for Agricultural Research. 2nd ed. New York: John Wiley and Sons; 1984.

27. Pereira DG, Afonso A, Medeiros FM. Over view of Friedman's test and post-hoc analysis. Communications in Statistics-Simulation and Computation. 2015Nov; 44(10): 2636–2653. doi:https://doi.org/10.1080/03610918.2014.931971"

28. Deshmukh KK, Aher S P. Assessment of soil fertility around municipal solid waste disposal site near Sangamner City, Maharashtra, India. Current World Environment Journal. 2017 May 20; 12(2): 401-410. doi: http://dx.doi.org/10.12944/CWE.12.2.24"

29. Brady NC, Weil RR. The nature and properties of soils. 13th ed. New Jersey: Pearson Education; 2002.

30. Havlin JL, Beaton JD, Tisdale SL, Nelson WL. Soil Fertility and Fertilizers: An Introduction to Nutrient Management. 8th ed. Pearson Education; 2013.

31. Ahmed I, Prasad Rao T V D, Hussain M. Quality assessment of soil at municipal solid waste dumpsite and possibilities of reclamation of land. International Journal of Innovative Science, Engineering and Technology. 2014 Sept; 1 (7): 274-294.

32. Sonneveld C, Voogt W. Plant Nutrition of Greenhouse Crops, Springer, ISBN 9048125316, New York, U.S.A. 2009.

33. Signore A, Serio F, Santamaria P. A targeted management of the nutrient solution in a soilless tomato crop according to plant needs. Frontiers in Plant Science. 2016 March 30; 7: 391. doi: https://doi.org/10.3389/fpls.2016.00391”

34. Samarakoon UC, Weerasinghe PA, Weerakkody AP. Effect of Electrical Conductivity [EC] of the Nutrient Solution on Nutrient Uptake, Growth and Yield of Leaf Lettuce (Lactuca sativa L.) in Stationary Culture. Tropical Agricultural Research. 2006 Jan 18; 18: 13–21. doi:https://dl.nsf.gov.lk/handle/1/12138”

35. Praveena GS, Rao PVV. Impact of leachate on soil properties in the dumpsite: A case study of greater Visakhapatnam. International Journal of Engineering Research and General Science. 2016 Jan-Feb; 4(1): 2091-2730. doi: https: //api. Semantic scholar. org/ CorpusID:106395088”

36. Krishna MK, Chaitra BR Kumari J. Effect of municipal solid waste leachate on the quality of soil. International Journal of Engineering Science Invention. 2016 Jun; 5(6): 69-72.

37. Zhang S, Lovdahl L, Grip H, Jansson P, Tong Y. Modelling the effects of mulching and fallow cropping on water balance in the Chinese Loess Plateau. Soil and Tillage Research. 2007 April; . 93 (2): 283-298. doi:10.1016/j.still.2006.05.002”

38. Jokela JPY, Rintala JA. Anaerobic solubilisation of nitrogen from municipal solid waste (MSW). Reviews in Environmental Science and Biotechnology. 2003 March; 2: 67-77. doi:https://doi.org/10.1023/B:RESB.0000022830.62176.36”

39. Soheil RI, Hossien MH, Gholamreza S, Hassan E, Jamei M, Etesami H. Effects of composted municipal waste and its leachate on some soil chemical properties and corn

plant responses. 2012 Jan; 2(6): 801-814. doi:http://www.ecisi.com"

40. Ahmad R, Muhammad D, Mussarat M, Fahad S, Ullah S, Ahmad T, et al. Effect of Different Levels of Nitrogen on Yield of Colocasia (Colocasia esculenta) at District Malakand Dargai. Open Journal of Soil Science. 2018 Feb 27;8: 87-98. doi:10.4236/ojss.2018.82007"

41. Sheikh S, Ishak CF. Effect of nitrogen fertilization on antioxidant activity of Mas cotek (Ficus deltoidea Jack). Journal of Medicinal Plants Studies. 2016 Jun 30; 4(4): 208–214.

42. Sokka L, Antikainen R, Kauppi P. Flows of nitrogen and phosphorus in municipal waste: a substance flow analysis in Finland. Progress in Industrial Ecology. 2004 Jan 16; 1: 165 - 186. doi:10.1504/PIE.2004.004677"

43. Fuentes B, Bolan N, Naidu R, Mora MDL. Phosphorus in organic waste soil system. Journal of Soil Science and Plant Nutrition. 2006; 6 (2): 64-83. doi:10.4067/S0718-27912006000200006"

44. Trankner M, Tavakol E, Jakli B. Functioning of potassium and magnesium in photosynthesis, photosynthate translocation and photoprotection. Physiologia Plantarum. 2018 April 18;163: 414–431. doi:10.1111/ppl.12747"

45. Gransee A, Fuhrs H. Magnesium mobility in soils as a challenge for soil and plant analysis, magnesium fertilization and root uptake under adverse growth conditions. Plant and Soil. 2012Dec 29; 368: 5-21. doi:10.1007/s11104-012-1567-y"

46. Shimada N. Excess Injury of Calcium and Magnesium in the Crops. Japan Agricultural

Research Quartely. 1973 Jul 01; . 7(3): 173-177.

47. Yadav BK. micronutrient status of soils under legume crops in arid region of Western Rajasthan, India. Academic Journal of Plant Sciences. 2011; 4 (3): 94-97.

48. Havlin HL, Beaton JD, Tisdale SL, Nelson WL. Soil fertility and fertilizers-an introduction to nutrient management. 7th ed. New Delhi: PHI Learning Private Limited; 2010.

49. Rout GR, Das P. Effect of Metal Toxicity on Plant Growth and Metabolism: I. Zinc. Agronomie. 2003 Jan; 23 (1): 3–11. doi:10.1007/978-90-481-2666-8_53"

50. Lucena JJ, Apaolaza LH. Iron nutrition in plants: an overview. Plant and Soil. 2017 Sept ; 418 (1-2). doi:10.1007/s11104-017-3316-8"

51. Landon JR. Booker tropical soil manual: A hand book of soil survey and agricultural land. Burnt Mill. UK: Booker agriculture international limited; 1991.

52. Onyango DA, Entila F, Dida MM, Ismail AM, Drame KN. Mechanistic understanding of iron toxicity tolerance in contrasting rice varieties from Africa: 1. Morpho-physiological and biochemical responses. Functional Plant Biology. 2019 Oct 29; 46(1):93-105. doi:10.1071/FP18129"

53. Meharg, A. 2011. Marschner's mineral nutrition of higher plants. Edited by P. Marschner. Amsterdam, Netherlands: Elsevier/ Academic Press. (2011), pp. 684, . ISBN 978-0-12-3849052. Experimental agriculture. 2012; 48(2):305. doi:10.1017/S001447971100130X"

54. Das DK. Micronutrients: their behaviour in soils and plants. Kalyani publishers; 2000

55. Adrees M, Ali S, Rizwan M, Ibrahim M, Abbas F, Farid M, et al. The Effect of Excess Copper on Growth and Physiology of Important Food Crops: A Review. Environmental Science and pollution Research. 2015 Jun 26; 22 (11): 8148–8162. doi:10.1007/s11356-015-4496-5"

56. Mir AR, Pichtel J, Hayat S. Copper: Uptake, Toxicity and Tolerance in Plants and Management of Cu-Contaminated Soil. Biometals. 2021 Aug; 34(4): 737–759. doi:10.1007/s10534-021-00306-z"

57. Stokes, P. M., Campbell, P. G. C., Schroeder, W. H., Trick, C., France, R. L, et al. 1988. Manganese in the Canadian environment. Ottawa, Ontario: National Research Council of Canada, Associate Committee on Scientific Criteria for Environmental Quality.

58. Neina D. The role of soil pH in plant nutrition and soil remediation. Applied and Environmental Soil Science. 2019 Nov 03. pp. 9. doi: https:// doi.org/ 10.1155/ 2019/ 5794869"

59. Radfard M, Yunesian M, Nabizadeh R, Biglar H, Nazmara S, et al. Drinking water quality and arsenic health risk assessment in Sistan and Baluchestan, Southeastern Province, Iran. Human and Ecological Risk Assessment. 2018 May;25(4): 1-17. doi:10.1080/10807039.2018.1458210

60. Kusin FM, Azani NNM, HasanS NMS, Sulong NA. Distribution of heavy metals and metalloid in surface sediments of heavily-mined area for bauxite ore in Pengerang, Malaysia and associated risk assessment. CATENA. 2018 Jun; 165: 454–464. doi:10.1016/J.CATENA.2018.02.029"

61. World Health Organization. Manganese in Drinking-Water: Background Document for Development of WHO Guidelines for Drinking-Water Quality 2004.Available from: https://iris.who.int/handle/10665/75376.

62. Pendias AK. Trace element in soils and plants. Boca Raton, FL, USA: CRC Press; 2000. doi:https://doi.org/10.1201/b10158"

63. Sharma RK, Agrawal M, Marshall F. Heavy metal contamination in vegetables grown in wastewater irrigated areas of Varanasi, India. Bulletin of Environmental Contamination and Toxicology. 2006 Sept; 77 (2):312-318. doi:10.1007/s00128-006-1065-0"

64. Das K K, Das SN, Dhundasi SA. Nickel, its adverse health effects & oxidative stress. Indian Journal of Medical Research. 2008 Oct;128: 412-425.

65. Ogundiran OO, Afolabi TAA. Assessment of the physicochemical parameters and heavy metal toxicity of leachates from municipal solid waste open dumpsite. International Journal of Environmental Science and Technology. 2008 March; 5 (2): 243-250. doi:10.1007/BF03326018"

66. Smith LA, Means JL, Chenetal A. Remedial Options for Metals-Contaminated Sites. , Boca Raton, Fla, USA: Lewis Publishers; 1995.

67. Scragg A. Environmental Biotechnology. 2nd ed. Oxford, UK: Oxford University Press; , 2006.

68. Haider FU, Liqun C, Jeffrey A, Coulter, Cheema SA, Wu J, Zhang R, Wenjun M, Farooq M. Cadmium toxicity in plants: Impacts and remediation strategies. Ecotoxicology and Environmental Safety. 2021; 211: doi: https:// doi.org/ 10.1016/ j.ecoenv.2020.111887"

69. Ugwu EI, Agunwamba JCA. Review on the Applicability of Activated Carbon Derived from Plant Biomass in Adsorption of Chromium, Copper, and Zinc from Industrial Wastewater. Environ. Monit. Assess. 2020; 192:240. doi:10.1007/s10661-020-8162-0”

70. Patra DK, Pradhan C, Patra HK. Chromium Bioaccumulation, Oxidative Stress Metabolism and Oil Content in Lemon Grass Cymbopogon Flexuosus (Nees Ex Steud.) W. Watson Grown in Chromium Rich over Burden Soil of Sukinda Chromite Mine, India. Chemosphere. 2019;218: 1082–1088. doi:10.1016/j.chemosphere.2018.11.211”

71. Rahman S, Qin A, Zain M, Mushtaq Z, Mehmood F, Riaz L, Naveed S, Ansari MJ, Saeed M, Ahmad I, Shehzad M. Pb uptake, accumulation, and translocation in plants: Plant physiological, biochemical, and molecular response: A review. Heliyon. 2024; 10(6). doi:https://doi.org/10.1016/j.heliyon.2024.e27724”

72. Hassan MU, Chattha MU, Khan I, Chattha MB, Aamer M, Nawaz M, Ali A, Khan MAU, Khan TA. Nickel toxicity in plants: reasons, toxic effects, tolerance mechanisms, and remediation possibilities-a review. Environ Sci Pollut Res Int. 2019; 26(13):12673-12688. doi:10.1007/s11356-019-04892-x”

73. Abbas G, Murtaza B, Bibi I, Shahid M, Niazi NK, Khan MI, Amjad M, Hussain M; Natasha. Arsenic Uptake, Toxicity, Detoxification, and Speciation in Plants: Physiological, Biochemical, and Molecular Aspects. Int J Environ Res Public Health. 2018;15(1):59. doi:10.3390/ijerph15010059”

928

Click here to access/download;Figure;Fig 7.tif

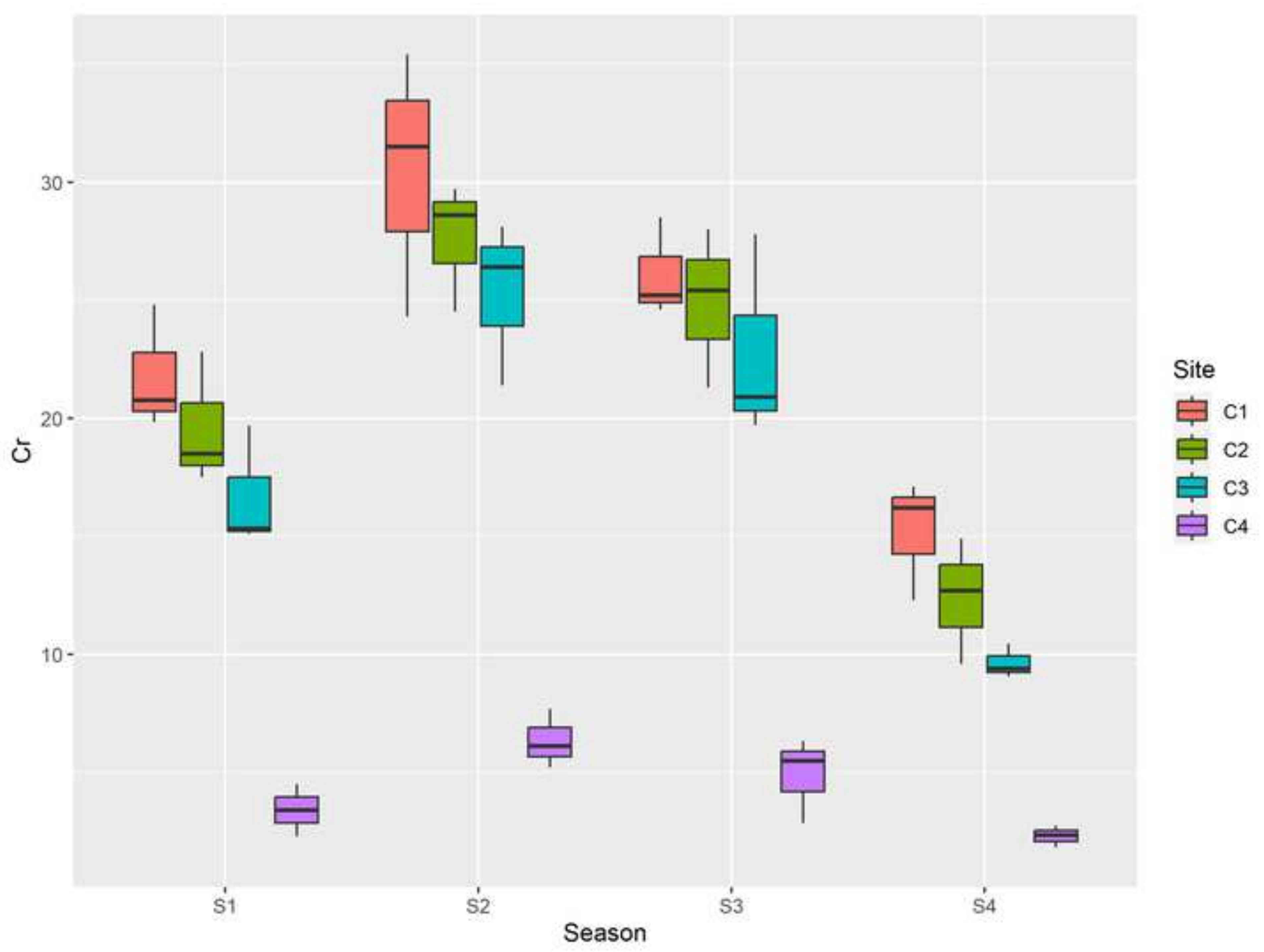

Click here to access/download;Figure;Fig 8.tif

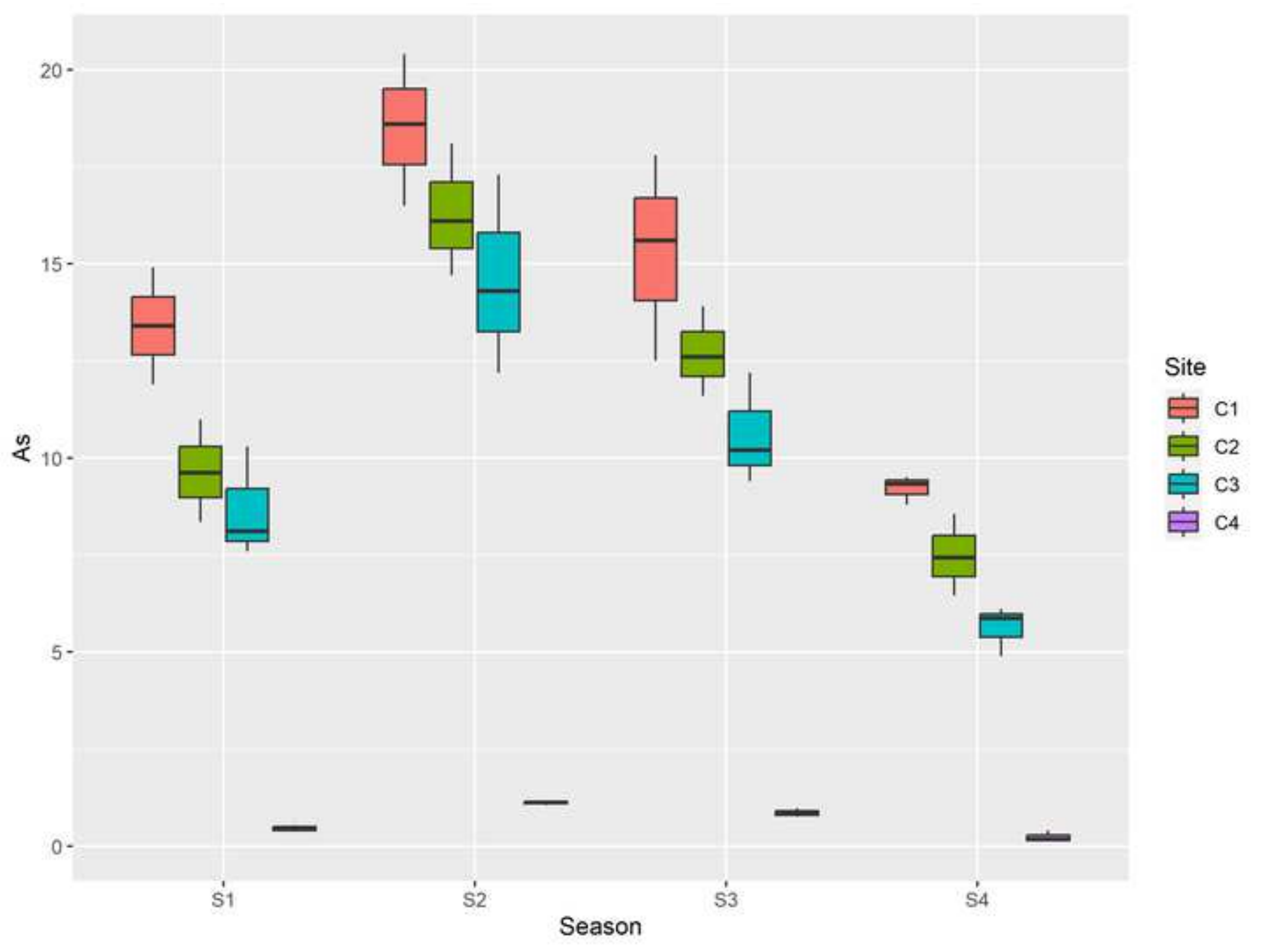

Click here to access/download;Figure;Fig 1.tif

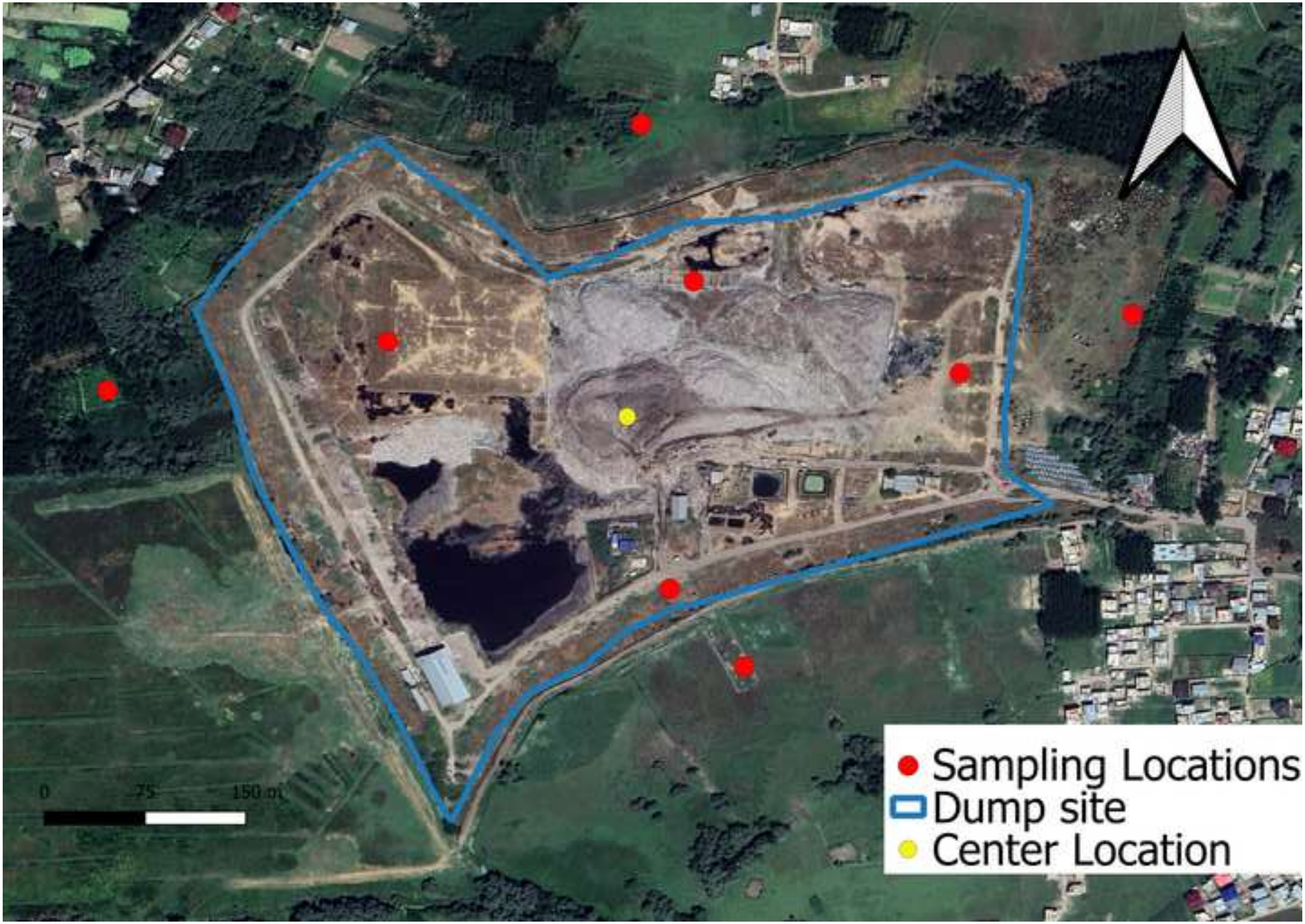

Click here to access/download;Figure;Fig 2.tif

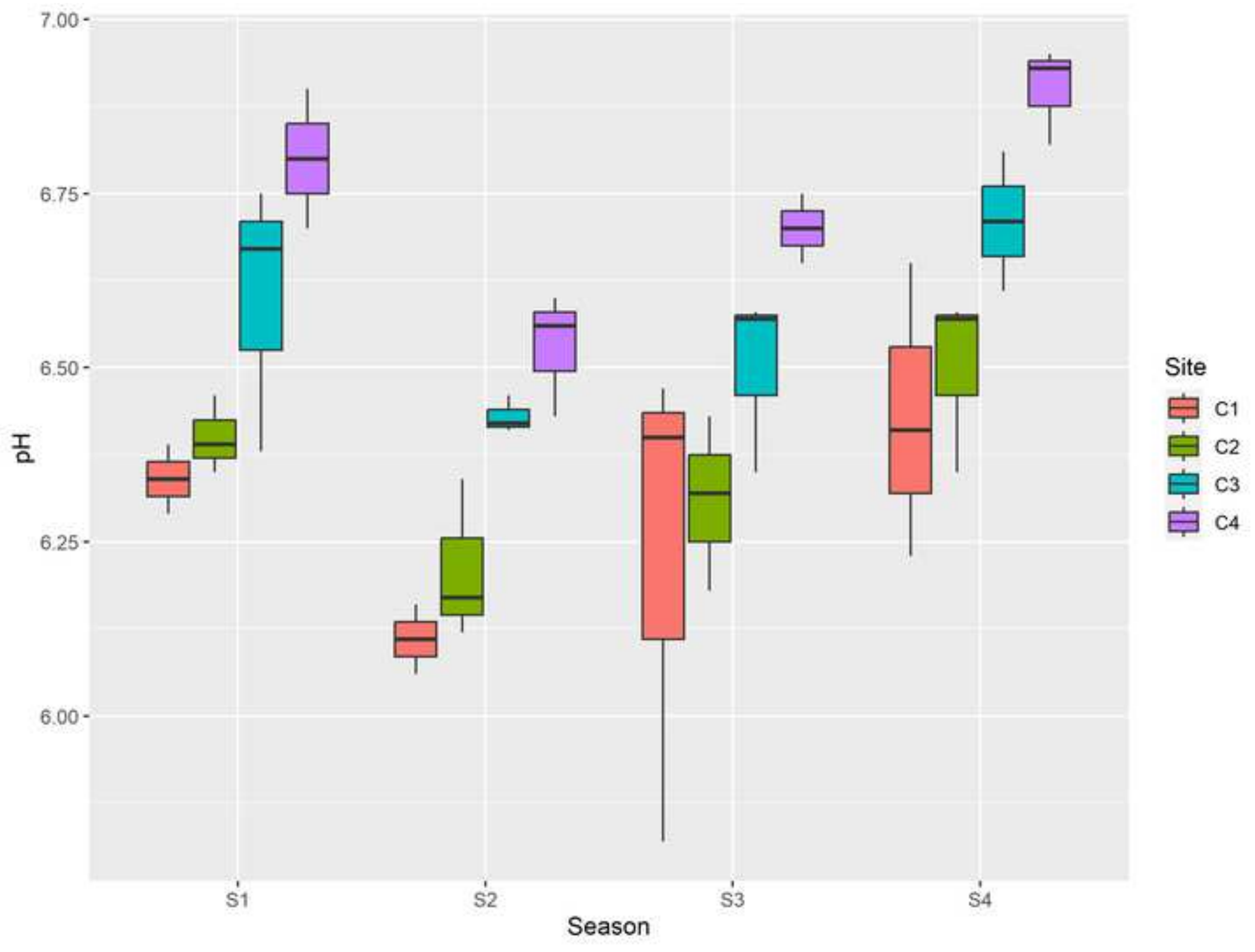

Click here to access/download;Figure;Fig 3.tif

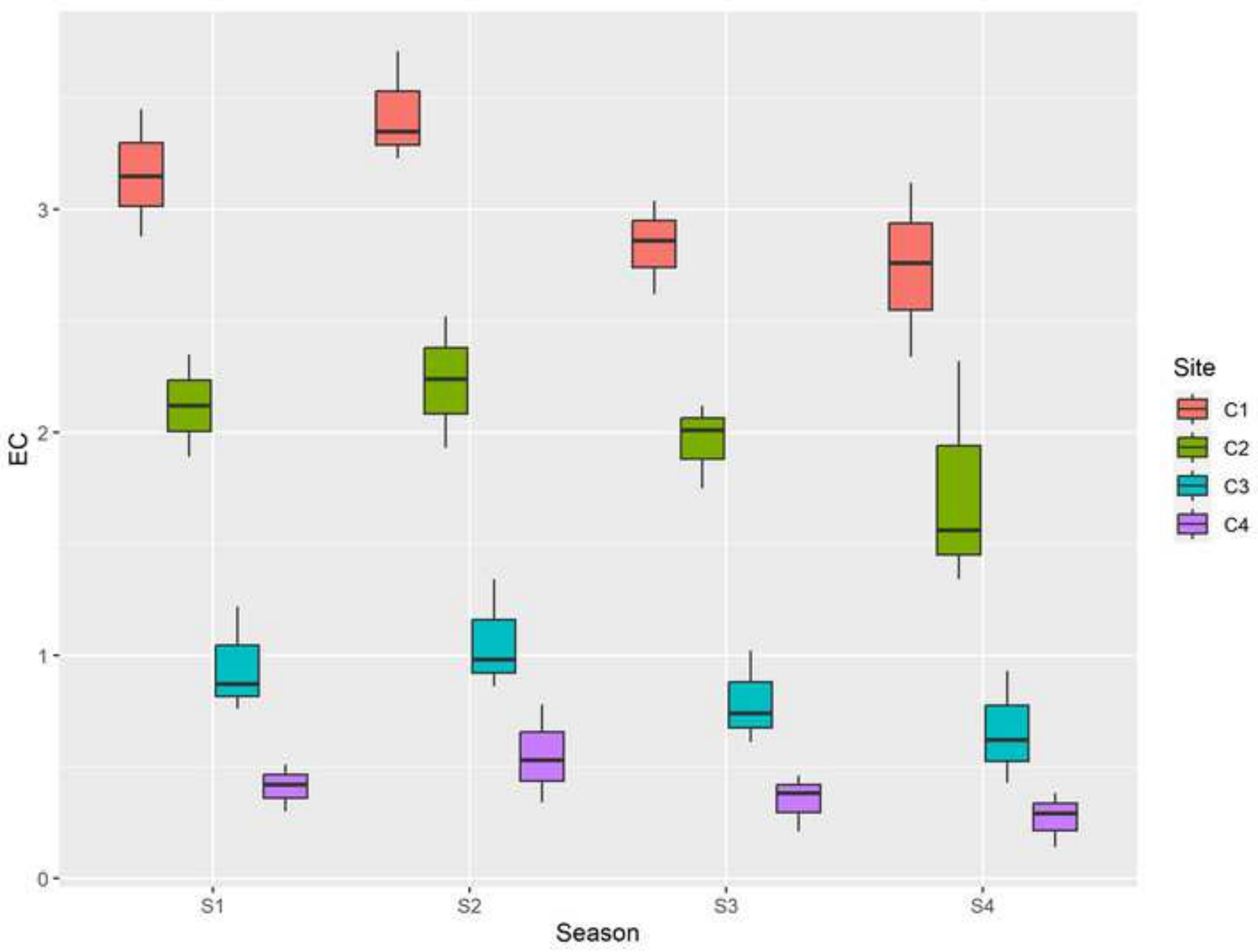

Click here to access/download;Figure;Fig 9.tif

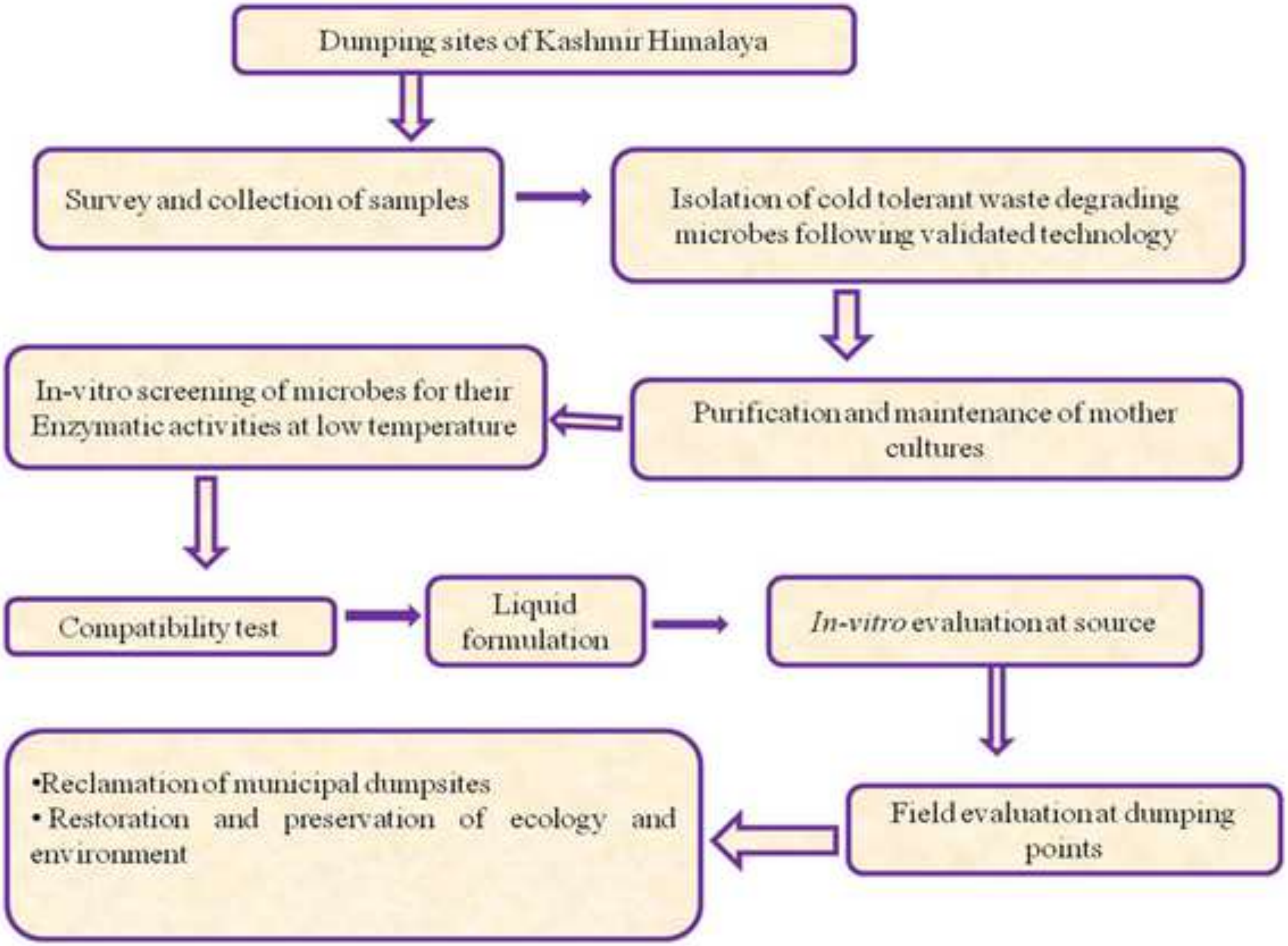

Click here to access/download;Figure;Fig 4.tif

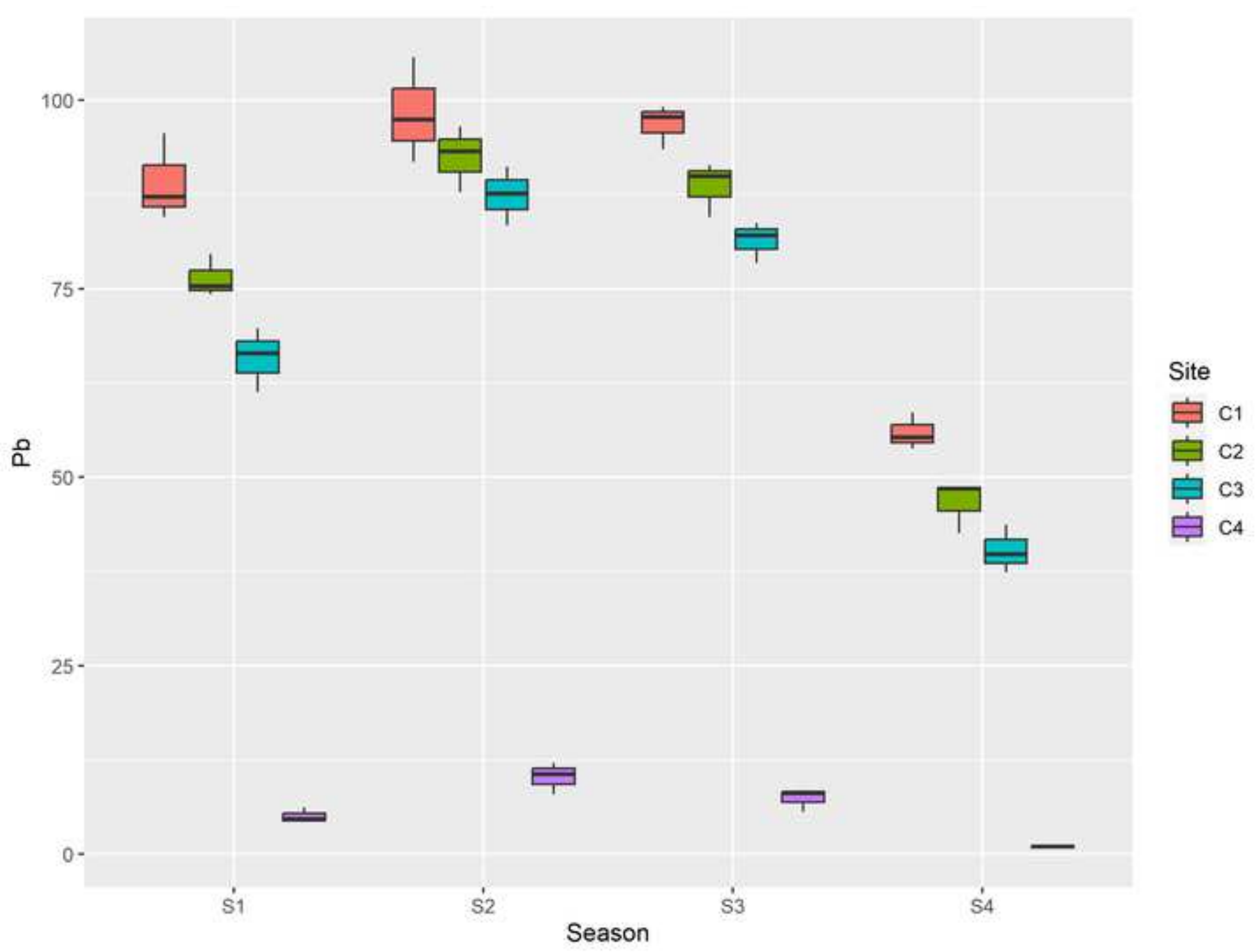

Click here to access/download;Figure;Fig 5.tif

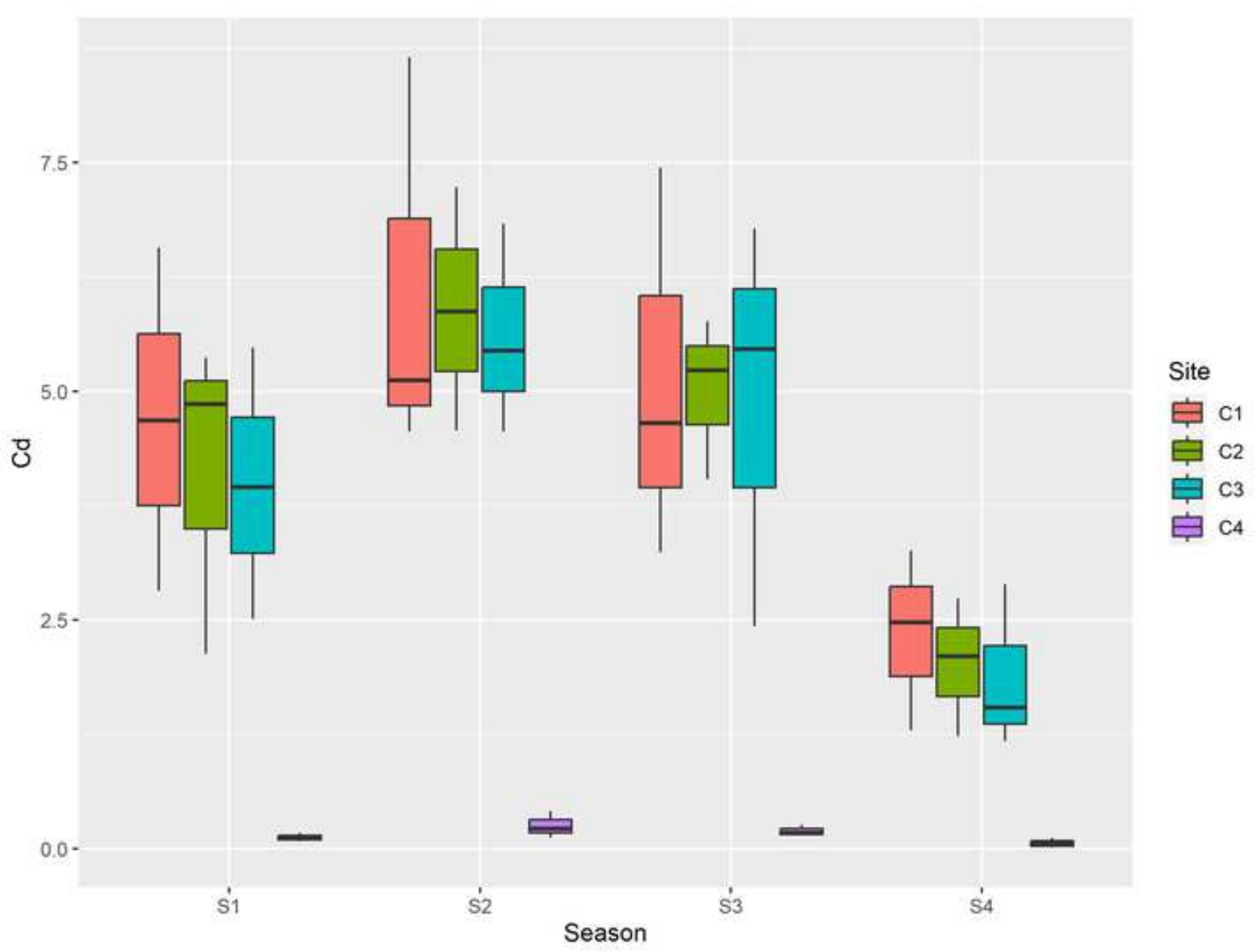

Click here to access/download;Figure;Fig 6.tif

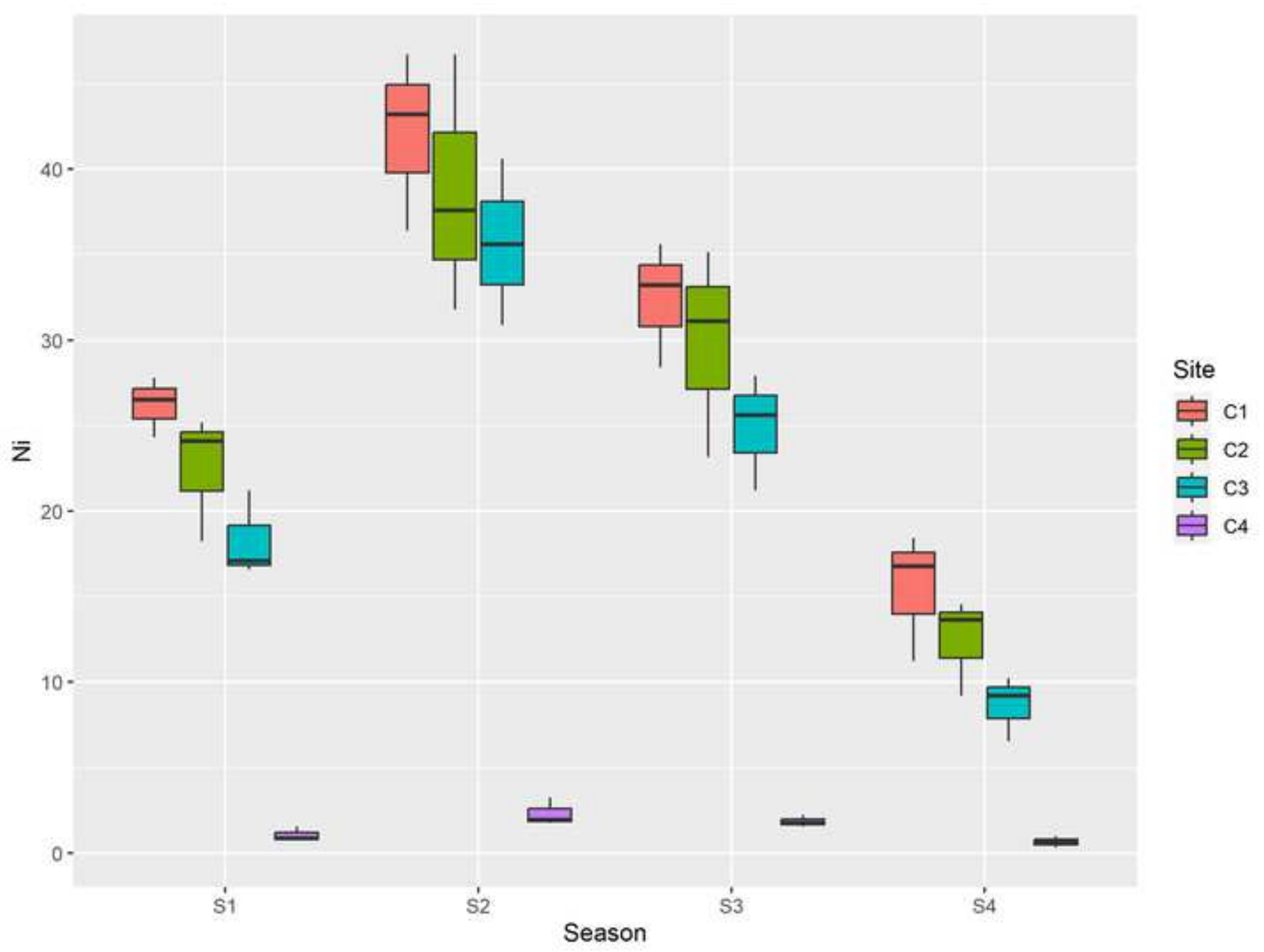

Click here to access/download
Supporting Information
Suplem. Data - PLOS.xlsx

Landfill leachate: an invisible threat to soil quality of temperate Himalayas

Shayesta Islam[1¶], Haleema Bano[1¶], Malik Ahsaf Aziz[2*], Fahad Alotaibi[3&]

1: Division of Environmental Sciences, SKUAST K, Srinagar, Jammu and Kashmir, India

2: Division of Basic Science and Humanities, SKUAST K, Srinagar, Jammu and Kashmir, India

3: Department of Soil Science, College of Food and Agriculture Sciences, King Saud University, P. O. Box 2460, Riyadh 11451, Saudi Arabia

*Corresponding author

Email id: drasifskuast@gmail.com (MAA)

[¶] These authors contributed equally to this work.

[&] This author also contributed equally to this work.

Abstract:

Landfills are the most affordable and popular method for managing waste in many parts of the world, However, in most developing nations, including India, the infiltration of hazardous materials from improperly managed dumping sites continues to be a significant environmental problem. Around the world, leachate is a significant point source of contamination in numerous environmental media, including soil, groundwater, and surface water. Soil is an important asset as it they is are the key factor s for food production and have tremendous significance in achieving sustainable development goals (SDGs). The contaminants from soil enter into food chain and ultimately reach humans. So in order to prevent the adverse effects of toxic elements on humans, there is need to maintain the soil quality and to prevent deterioration. Keeping in view the consequences of unscientific management of waste, the goal of the experiment was to determine how landfill leachate from Achan landfill affected the soil quality in the temperate Himalayas. All four seasons of the year, viz Spring, Summer, Autumn, and Winter, at four sites viz, Center of dumping site, inside, Outside and Control were monitored. Among sites center was

found to have maximum value of EC (3.04 dS/m), Moisture content (42.51%), N (285.43 mg/kg), P (70.07 mg/kg), K (265.71 mg/kg), Ca (957.67 mg/kg), Mg(402.42 mg/kg), Zn (2.02 mg/kg), Fe (10.56 mg/kg), Cu (2.07 mg/kg), Mn (10.73 mg/kg), Pb (85.02 mg/kg), Cd (4.50 mg/kg), Ni (29.04 mg/kg), Cr (23.37 mg/kg), As (14.10 mg/kg). While as the lowest value of all parameters was reported at control site. ~~Among seasons summer was reported to have maximum value of EC (1.818 dS/m), N (264.11 mg/kg), P (60.98 mg/kg), K (239.70 mg/kg), Ca (913.52 mg/kg), Mg (385.09 mg/kg), Pb (72.1 mg/kg), Cd (4.46 mg/kg), Ni (29.70 mg/kg), Cr (22.41 mg/kg), As (12.63 mg/kg), While as lowest value of these parameters was reported during winter season. Among sites Minimum pH (6.2) was reported at center of dumping site and maximum (6.7) was reported at control site, while as among seasons maximum pH was reported during winter season (6.6) and minimum (6.3) was reported during summer season. Zn, Fe, Cu and Mn were reported maximum during winter season and minimum during summer season.~~ From the study it is recommended that the waste generated is mostly organic (65-75%), thus can be segregated and treated at source. The waste can be treated at source using microbial consortium technology in order to transform the waste in to wealth in a sustainable way and to prevent the ~~soil quality~~ ~~deteroriation~~ deterioration of soil quality.

Key words: Landfill, Leachate, Soil quality, Heavy metals

Introduction:

Soil ecosystems are the foundation for food production and are crucial for achieving sustainable development goals (SDGs), so are regarded as significant environmental assets [1]. It supports or carries out a variety of tasks, such as biomass production, storing, filtering, and changing a wide variety of materials, including water and nutrients [2]. The majority of the carbon (C) and nutrient elements that support life are generated by soils, which also act as a

repository for them [3]. Soils are also the pedestal that plants rely on to remain upright. They also serve as the habitat for a vast biodiversity and biomass of soil organisms [4]. Soils hold the water that plants and soil creatures need to exist and thrive, and they restrict the pace of water movement, limiting erosion and soil loss [5]~~.~~ It has been reported that improving soil health through various amendments like biochar application improves soil physicochemical properties and enzymatic activities [6]. ~~Furthermore soil health is an integrative quality that indicates the soil's ability to respond to agricultural intervention in order to continue supporting agricultural production as well as the supply of other ecosystem services. Soil conditions are known to influence plant mineral and trace element concentration [7], which is the human dietary supply, as humans require at least 25 mineral elements for good health [8].. The food and feed demand of an ever-increasing population can be satisfied by preserving soil health.~~

~~Soil health is important for sustainable development of agricultural systems to meet the needs of increasingly growing population as healthy soil suppress pathogens, sustain biological activities, decompose organic matter, inactivate toxic materials and recycle nutrient, energy, and water [9]. Healthy soils have significantly enhanced nutrient content and desirable abundance of bacterial phylum paving way for sustainable production [10].~~ But this valuable asset is ~~C~~currently under ~~the~~ serious threat due to ~~to this soil ecosystem is posed by~~ generation of excessive quantity of waste due to rapid population growth and urbanization. According to the reports of world bank, the world wide generation of municipal solid waste in 2018 was 1.3 billion tons per year and is expected to reach approximately 2.2 billion tons by 2025 [7~~11~~]. Kashmir Valley is also grappling with significant challenges stemming from increasing waste generation. Srinagar city alone produces 0.526 kg of waste per capita per day, with Anantnag following at 0.479 kg, Ganderbal at 0.400 kg, and Budgam at 0.397 kg respectively. The total

annual waste generated across these districts amounts to 57,199.99 Metric Tonnes (MT), with Srinagar producing the highest at 236,732.75 MT and Budgam the lowest at 42,840.00 MT 8[12]. This enormous amount of waste can pose great challenge especially to low- and middle-income countries and developing nations.

The waste produced from various sources is disposed of by public to the landfill, where it undergoes biodegradation as a result of various physical, chemical, and biological processes resulting in the release of leachate. Leachate is a dark brown liquid with a stinking smell and contains soluble and suspended material. The leachate is composed of soluble organic and inorganic compounds, nutrients, suspended particles, heavy metals, and many hazardous chemicals, causing significant damage to both natural and agricultural ecosystems when released in an untreated and uncontrolled manner [13, 14]. The leachate from these waste dumping sites have adverse effects on soil quality owing to high concentration of nutrients, heavy metals, and soluble salts [13,14]

The immense volume of waste generated from various sources ultimately finds its way to landfills, with Srinagar city in the Kashmir Valley being no exception. Here, all the waste produced across the city is disposed of at the Achan landfill site, the sole dumping ground for Srinagar. This site, located in the northern part of the city, between the coordinates 34° 09′ N latitude and 74° 79′ E longitude, lies approximately 5-6 kilometers from the city's center.

Before 1985, Srinagar produced minimal waste, free from the burden of plastic. Back then, the Srinagar Municipal Corporation (SMC) disposed of the city's waste either in the Noorbagh grounds or along the banks of the river Jhelum. However, with the advent of stricter environmental policies, the Government of Jammu and Kashmir, following state cabinet approval, transferred around 30.35 hectares (75 acres) of state land to the SMC for waste disposal at Achan [9]. By 1986, this site became the primary location for the open dumping of municipal solid waste.

Formatted: Justified, Line spacing: 1.5 lines

For many years, vast amounts of refuse were deposited here without consideration for the environmental consequences. Achan remained the sole dumping ground for Srinagar until 2008 [10], with waste from 518 collection points across the city being transported to this location [9].

Once a thriving wetland, home to vibrant birds and lush vegetation, Achan has since become a source of pollution, contaminating water bodies, air, and soil. The nearby population has also been affected, suffering from various diseases. The waste deposited at the site undergoes biodegradation through a combination of physical, chemical, and biological processes, leading to the production of leachate—a dark brown liquid with a foul odor. This leachate contains dissolved organic and inorganic compounds, nutrients, suspended particles, heavy metals, and hazardous chemicals. When left untreated and uncontrolled, it poses a serious threat to natural and agricultural ecosystems, significantly degrading soil quality due to its high concentrations of nutrients, heavy metals, and soluble salts [11, 12].

Formatted: Normal (Web), Indent: First line: 0", Line spacing: 1.5 lines

Formatted: Font color: Auto

Contaminated soil affects ecosystems and human, plant and animal health in a number of ways [13~~15~~]. The acidic leachate formed due to production of organic acids liberated by the degradation of waste, leads to the acidic surroundings that destroys micro-organisms, which are helpful in improving the composition of the soil. Furthermore contaminated soil has the potential to alter agricultural metabolism and diminish crop yields by forcing trees and crops to absorb soil pollutants [14~~16~~]. In addition to this, contaminated soils with high nitrogen and phosphorus levels will leach into waterways, leading in death of aquatic organisms by diminishing the amount of dissolved oxygen [15~~17~~]. The metals in leachate are common environmental pollutants that are non-biodegradable, can deplete available soil resources, and have a negative impact on plant growth and yield [16~~8~~,17~~9~~]. .

Keeping in view the far reaching consequences of landfill leachate and contaminated soil and need for global attention on improving or restoring soil health, it is imperative to evaluate the affect of leachate on soil physico chemical properties for proper management strategy to

prevent soil degradation and alleviate the threats posed by the expansion of waste. Understanding and estimating the quality of leachate-impacted soils can establish an opportunity to judge the sustainability of land management and land-use systems. . In this context, the present study aimed to evaluate the influence of landfill leachate from Srinagar's Achan dump on soil physico-chemical parameters across different seasons and sites.

Study area:

~~The study was carried out in Achan landfill, which is located in Srinagar city of J and K UT at an elevation of 1600m above mean sea level with coordinates between 74°41' 6" and 74°57' 27" East Longitude and 33° 59' 14" and 34°12' 37" North Latitude as depicted in Fig 1. SMC unloads 350 metric tonnes of rubbish per day at this location, out of 450 metric tonnes generated daily, and waste generation is predicted to rise 1723 metric tonnes per day by 2035. In Srinagar, 62% of total garbage generated per day is organic waste, while the rest is inorganic, including 7% of plastic waste [20]). 65-70% of municipal solid waste generated in Srinagar city is collected by door-to-door collection method and street bin systems and is transported for dumping to this site and the remaining 30-35% of waste is dumped illegally into depressions, river embankments, unattended open spaces or is locally burnt both by individuals or Safia Karamcharis creating nuisance for public as well as acting as breeding centers of some diseases [21]. Due to lack of proper management and handling of waste, heaps of wastes have been deposited. There is a lot of foul smell creating a nuisance for surrounding inhabitants. During the rainy season the leachate percolates in the soil affecting the soil physico- physico-chemical properties and has made the surrounding soil unfit for agriculture.~~

The study was conducted at the Achan landfill, located in Srinagar city, Jammu and Kashmir UT, at an elevation of 1600 meters above mean sea level. The landfill's coordinates range between 74°41' 6" and 74°57' 27" East Longitude and 33° 59' 14" and 34°12' 37" North Latitude, as shown in Fig 1.

Formatted: Justified, Line spacing: 1.5 lines

Fig 1: **Map of study area (Map prepared by USGS)**

Formatted: Normal, Justified

Formatted: Font: Bold, Font color: Text 1

Spanning an area of 30.35 hectares (75 acres), the landfill is situated amidst residential settlements and water bodies, directly impacting the lives of residents in nine villages: Saidapora, Shonglipora, Waganpora, Sangam, Braywar, Danmar, Guzerbal, Noorshah Colony, and Bagh-i-Lal Pandith.

Formatted: Justified, Line spacing: 1.5 lines

Every day, the Srinagar Municipal Corporation (SMC) disposes of 450 metric tonnes in this site. Waste generation is projected to surge to 1,723 metric tonnes per day by 2035. Currently, 62% of the total waste produced daily in Srinagar consists of organic material, with 7% being plastic waste [18].

Approximately 65-70% of Srinagar's municipal solid waste is collected through door-to-door methods and street bin systems, and then transported to the Achan landfill. However, the remaining 30-35% is illegally discarded into depressions, river embankments, open spaces, or is burned locally by individuals and Safai Karamcharis. This not only causes a public nuisance but also creates breeding grounds for various diseases [19].

Due to inadequate waste management and improper handling, large heaps of waste have accumulated at the site, emitting a foul odor that disturbs nearby residents. During the rainy season, leachate from the waste seeps into the soil, altering its physico-chemical properties and rendering the surrounding land unsuitable for agriculture.

Materials and methods ~~ethodology:~~

~~Soil samples were collected from Achan dumpsite Srinagar during all the four seasons of year *viz*, spring, summer, autumn and winter after taking proper permission from Srinagar~~

Municipal Corporation, Head office Kara Nagar, Srinagar, Jammu and Kashmir . The number of sampling sites selected was four *viz,* Center of dumping site, inside (four cardinal points inside dumpsite), outside (four cardinal points outside dumpsite), and control site was taken at Shalimar campus of SKUAST Kashmir. At each site three replications were taken. The soil samples were taken in separate moisture cans in order to retain moisture content. The soil samples were then dried in shade, after drying these were grinded and then sieved through 2mm sieve. After this different physico-chemical parameters were analyzed. The gravimetric method by Prihar and Sandhu [22] was used to measure soil moisture. With the aid of a pH meter and an electrical conductivity meter, soil pH and electrical conductivity were measured using the potentiometric approach [23] . The potassium permanganate method was used to calculate the nitrogen content of the soil [24]. Olsen et al.[25] spectrophotometer method was used to measure the amount of available phosphorus. The method outlined by Jackson [23] was used to calculate the potassium that was accessible. Utilizing the Versenate method described by Chesin and Yein [26], calcium and magnesium were measured. Using the DTPA extraction process and an atomic absorption spectrophotometer (AAS), Lindsay and Norwell's method [27] was used to detect the presence of heavy metals.

Soil samples were collected from the Achan dumpsite in Srinagar throughout all four seasons of 2022—spring, summer, autumn, and winter—following proper authorization from the Srinagar Municipal Corporation, headquartered at Kara Nagar, Srinagar, Jammu and Kashmir. Four distinct sampling sites were chosen: the center of the dumpsite, inside the dumpsite (four cardinal points within), outside the dumpsite (four cardinal points outside), and a control site located at the Shalimar campus of SKUAST Kashmir. These sites were selected to assess the impact of leachate produced from the waste on soil properties at varying distances from the dumping points. Three replicates were collected from each site.

Formatted: Justified, Line spacing: 1.5 lines

Samples were taken solely from the soil, as leachate was directly seeping into the ground. The soil was collected in separate moisture cans to preserve its moisture content. Once collected, the samples were air-dried in the shade, then ground and sieved through a 2mm mesh before analyzing various physico-chemical parameters. Soil moisture content was determined using the gravimetric method by Prihar and Sandhu [20], while soil pH and electrical conductivity were measured using the potentiometric method with a pH meter and an electrical conductivity meter [21]. Nitrogen content was determined via the potassium permanganate method [22], and the available phosphorus was measured using the spectrophotometric method by Olsen et al. [23]. The Jackson method [21] was used to quantify available potassium, while calcium and magnesium levels were assessed using the Versenate method, as outlined by Chesin and Yein [24]. Heavy metal concentrations were determined using the DTPA extraction process and an atomic absorption spectrophotometer (AAS), following the method of Lindsay and Norwell [25].

~~**Ethics approval and consent to participate:** Wherever necessary, appropriate approval ethics have been applied.~~

Statistical analysis

~~:~~ The data in the table are given as an interactive mean of sites and seasons in accordance with the usual technique outlined by Gomez and Gomez~~—~~[26~~8~~]. Two-way ANOVA (analysis of variance) was performed at a 5% level of significance to test for differences across sites and seasons. In the univariate statistical pairwise comparison between all the samples comparing the means of two populations that are seasons and sites in our situations, analysis of variance with post hoc testing (Duncan's multiple range test) is performed [27~~9~~]. The significance is shown by the various letters, and vice versa [27~~9~~].

Results ~~and Discussion:~~

Soil pH determines the acidity or alkalinity of soil solutions and refers to the soil's hydrogen ion content. Natural soil typically has a pH between 7 and 8.5, although this range can vary based on biological activity, temperature, and municipal waste disposal practices [30]. Soil pH influences the availability of essential plant nutrients and the concentration of toxic elements, thereby affecting plant growth [31]. As it impacts the availability of all nutrients in the soil, soil pH has a direct effect on the survival and development of plants.In this, study pH of dumpsite soils ranged from 6.1 to 6.9 throughout study period as depicted in Table 1. The mean pH values ranged from slightly acidic to neutral, which may be due to the decomposition of organic matter leading to the formation of organic acids. However, the pH increased with increase in distance from dumpsite which may be attributed to release of less organic acids away from dumpsite. Further, the lowest pH value (6.3) reported during summer, followed by autumn (6.4), spring (6.5) and highest (6.6) during winter season as depicted in Table 1 Fig 2, may be attributed to more degradation of organic matter leading to increase in production of organic acids because of increased microbial activity and favorable temperature and moisture conditions during summer season as compared to winter season. The pH value of control site (Shalimar) soil was higher than the other three sites during all the seasons, this may be due to presence of less organic matter and hence less microbial activity. The slightly acidic pH of the soils at three other sites suggests that solid waste contributed to soil acidity, leading to a decrease in the availability of essential plant nutrients such as phosphorus and molybdenum, while increasing the availability of toxic elements, particularly aluminum and manganese. The pH values varied significantly between summer, autumn, and winter, but there was no significant difference between spring and autumn. Additionally, the pH values at different locations inside, outside,

~~and control — were significantly different, though there was no significant difference between the center and inside locations.~~

pH determines the acidity or alkalinity of soil solutions and refers to the soil's hydrogen ion content. In our study among seasons maximum mean pH was recorded in winter (6.6), followed by spring (6.5), autumn (6.4) and minimum was recorded in summer season (6.3). Among sites maximum mean pH was recorded in control (6.7), outside (6.6), inside (6.3) and minimum (6.2) was recorded at center as depicted in Table 1. The pH values varied significantly between summer, autumn, and winter, but there was no significant difference between spring and autumn. Additionally, the pH values at different locations—inside, outside, and control—were significantly different, though there was no significant difference between the center and inside locations. During this study mean EC of dumpsite soil was found to deviate from 0.27 dS/m to 3.43 dS/m.Maximum mean value of EC among seasons was recorded in summer (1.81 dS/m), spring (1.66 dS/m), autumn (1.48 dS/m) and minimum was recorded in winter (1.35 dS/m). Among sites maximum mean value of EC was recorded at center (3.04 dS/m), followed by inside (2.01 dS/m), outside (0.86 dS/m) and minimum was recorded at control (0.40 dS/m). Moisture content was recorded maximum in winter (36.95 %) among seasons, followed by spring (32.48%), autumn (29.34 %) and minimum was recorded in summer (25.23 %). Among sites mean maximum moisture content was recorded at center (42.51%), followed by inside (34.07 %), outside (28.83%) and minimum was recorded at control site (18.58%). The EC values during spring, summer, autumn and winter were significantly different. In addition to this EC values at all sites were significantly different.

In this study, the mean moisture content values ranged from 12.87% to 48.42%.

The moisture content values between all seasons were significantly different, in addition this, the moisture content values between all sites were also significantly different.

Formatted: Font:

Table 1. Seasonal and site wise variation in pH, EC (dS/m) and Moisture content (%) of soil

**Parameters**	**Seasons**	**Sites**				**Mean**	**C.D (P≤0.05)**
		**Center**	**Inside**	**Outside**	**(Control)**		
**pH**	Spring	6.3±0.05^{fgh}	6.4±0.09^{efg}	6.6±0.06^{bc}	6.8±0.10^{ab}	**6.5^{b}**	Sites: 0.12<br>Seasons: 0.12<br>Sites × Seasons: 0.24
	Summer	6.1±0.05^{h}	6.2±0.11^{gh}	6.4±0.06^{cdef}	6.5±0.14^{bcde}	**6.3^{c}**	
	Autumn	6.2±0.35^{gh}	6.3±0.09^{fgh}	6.5±0.04b^{cd}	6.7±0.18^{ab}	**6.4^{b}**	
	Winter	6.4±0.21^{defg}	6.5±0.13^{cdef}	6.7±0.05^{ab}	6.9±0.15^{a}	**6.6^{a}**	
	**Mean**	**6.2^{c}**	**6.3^{c}**	**6.6^{b}**	**6.7^{a}**		
**EC**	Spring	3.16±0.28^{ab}	2.12±0.23^{cd}	0.95±0.24^{ef}	0.41±0.10^{gh}	**1.66^{ab}**	Sites: 0.21<br>Seasons: 0.21<br>Sites× Seasons:0.42
	Summer	3.43±0.25^{a}	2.23±0.29^{c}	1.06±0.24^{e}	0.55±0.22^{fgh}	**1.818^{a}**	
	Autumn	2.84±0.21^{b}	1.96±0.19^{cd}	0.79±0.20^{efg}	0.35±0.13^{h}	**1.485^{bc}**	
	Winter	2.74±0.26^{b}	1.74±0.51^{d}	0.66±0.25^{efgh}	0.27±0.06^{h}	**1.35^{c}**	
	**Mean**	**3.04^{a}**	**2.01^{b}**	**0.86^{c}**	**0.40^{d}**		
**Moisture content (%)**	Spring	43.34±1.01^{b}	36.48±0.87^{d}	29.44±0.47^{g}	20.67±1.01^{j}	**32.48^{b}**	Sites:1.797<br>Seasons: 1.797<br>Sites× Seasons:3.594
	Summer	36.87±0.99^{d}	26.65±0.99^{h}	24.55±0.97^{i}	12.87±0.69^{l}	**25.23^{d}**	
	Autumn	41.43±1.01^{c}	31.37±1.03^{f}	28.09±0.99^{gh}	16.48±1.01^{k}	**29.34^{c}**	
	Winter	48.42±1.01^{a}	41.79±1.02^{bc}	33.27±1.00^{e}	24.32±1.01^{i}	**36.95^{a}**	
	**Mean**	**42.51^{a}**	**34.07^{b}**	**28.83^{c}**	**18.58^{d}**		
Standard limits (ICAR manual)	pH<br>Acidic < 6.0<br>Normal to saline (6.0-8.5)<br>Tending to became alkaline (8.9 -9.0)<br>Alkaline > 9.0	**EC**<br>Low <1.0 dS/m<br>Medium (1.0-3.0 dS/m)<br>High > 3.0 dS/m				Moisture content<br>Low (3- 10%)<br>Medium (20 – 40 %)<br>High > 40 %	

~~EC: Electrical conductivity~~

Formatted: Font: 12 pt
Formatted: Font: 14 pt
Formatted: Left
Formatted Table
Formatted: Font: 12 pt
Formatted: Left

Mean N concentration in soils of the experimental site ranged from 110.67 to 320.49 (mg/kg). The concentration of N was maximum at center of dumping site (285.43 mg/kg), followed by inside (266.73 mg/kg), outside (241.61 mg/kg) and least (131.26 mg/kg) was reported at control site during all the seasons. Among seasons the maximum N concentration was observed during summer season (264.11 mg/kg), followed by spring season (244.93 mg/kg), autumn season (221.85 mg/kg) and least (194.14 mg/kg) was observed in winter season as depicted in Table 2. Additionally, the mean values of N between sites change significantly, and the mean values of N between all seasons were significantly different.

Soil phosphorus content at the experimental site ranged from 15.38 to 87.36 mg/kg. The highest mean P level was found at the center of the dumping site (70.07 mg/kg), followed by the inside (55.12 mg/kg) and outside (41.16 mg/kg) locations, with the lowest at the control site (22.16 mg/kg), as shown in Table 2. Seasonally, the highest mean phosphorus content was observed during the summer (60.98 mg/kg), followed by spring (49.51 mg/kg), autumn (41.85 mg/kg), and the lowest during winter (36.15 mg/kg). Additionally, there are considerable differences in the mean values of P between locations and the mean values of P throughout all seasons.

Soil potassium levels ranged from 96.54 to 301.28 mg/kg. Among seasons maximum mean potassium (**239.70** mg/kg) was recorded in summer season followed by spring (**222.63** mg/kg), Autumn (204.14 mg/kg) and minimum (**183.58 mg/kg**) was recorded in winter season. Among sites mean maximum value of potassium was recorded in center (**265.71 mg/kg**), followed by inside (**242.35 mg/kg**), outside (**225.85 mg/kg**) and minimum (**116.16 mg/kg**) was recorded at control site as depicted in Table 2. Additionally, there are considerable differences in

the mean values of K between sites as well as the mean values of K across all seasons.

Electrical conductivity is an indirect measurement that is correlated with a number of the physical and chemical characteristics of soil. The quantity of moisture that soil particles can hold affects the electrical conductivity of the soil. The total quantity of anions and cations determines electrical conductivity. It also depends on the primary salts in the soil solution, which have a negative impact on the qualities of the soil [32]. The optimal EC is crop specific, and depends on environmental conditions [33]. In general, higher EC hinders nutrient uptake by increasing the osmotic pressure of the nutrient solution, wastes nutrients, and the increases discharged of nutrients into the environment, resulting in environmental pollution. Lower EC may severely affect plant health and yield [34, 35]. During this study mean EC of dumpsite soil was found to deviate from 0.27 dS/m to 3.43 dS/m. The maximum mean value of EC was reported at center of dumping site (3.04 dS/m), which may be due to increased concentration of cations and anions within the dumpsite soil [36] and minimum (0.40 dS/m) was reported at control site as depicted in Table 1. Among seasons maximum mean EC value was reported during summer season (1.818 dS/m), which may be due to increase in degradation of salts and release of large number of cations and anions favoured by temperature and minimum was reported during winter (1.35dS/m) due to decrease in degradation of organic matter as shown in table 1 and Fig 3. The electrical conductivity of the soil samples collected within the dumpsite was high compared to the soil sample collected from the control site. The EC values during spring, summer, autumn

and winter were significantly different. In addition to this EC values at all sites were significantly different.

Formatted: Normal (Web), Space Before: 20 pt, After: 20 pt, Widow/Orphan control, Pattern: Clear (White), Tab stops: Not at 0.49"

Moisture content refers to the amount of water soil can hold. Most soil samples from municipal solid waste have moisture content ranging from 15% to 40% [32]. In this study, the mean moisture content values ranged from 12.87% to 48.42%. The highest mean moisture content was observed at the center of the dumping site (42.51%), followed by the inside (34.07%) and outside (28.83%) locations, with the lowest at the control site (18.58%), as shown in Table 1 and Figure 4. This is likely because dumpsite soil retains more moisture due to the natural ground being covered by municipal solid waste, which has a high moisture content and prevents soil moisture from evaporating directly [37]. The mean moisture content of soil decreased with the increase of the distance away from the refuse dump. This higher moisture content in the dumpsite soil may be associated with the presence of high organic matter which has capacity to hold water [38]. This excess amount of water suffocates plant roots by reducing the amount of oxygen available to them. Without enough oxygen, roots are not able to absorb nutrients and water effectively, which in turn inhibits the growth of plants. The moisture content was high during winter season (36.95%), followed by spring season (32.48%) and autumn season (29.24%) and lowest was observed during summer season (25.23%). This may be attributed to the high precipitation rate during winter as shown in Fig 4. The moisture content values between all seasons were

significantly different, in addition this, the moisture content values between all sites were also significantly different.

Nitrogen (N) is the most important ingredient that plants take up from the soil. The level of nitrate in the soil rises due to higher utilization of nitrogenous fertilizers. In the present experiment, mean N concentration in soils of the experimental site ranged from 110.67 to 320.49 (mg/kg). The concentration of N was maximum at center of dumping site (285.43 mg/kg), followed by inside (266.73 mg/kg), outside (241.61 mg/kg) and least (131.26 mg/kg) was reported at control site during all the seasons as shown in Table 2. The samples within the dumpsite have high N concentration than the samples away from dumpsite. This may be attributed to abundant amount of the putrescible waste (bio waste, kitchen waste) [39]. The maximum N concentration was observed during summer season (264.11 mg/kg), followed by spring season (244.93 mg/kg), autumn season (221.85 mg/kg) and least (194.14 mg/kg) was observed in winter season. This may be due to increase in reaction rate because of high temperature. Additionally, the high levels of organic matter present in dump soil may be responsible for the high N content [40]. It has been reported that application of N significantly increased tuber yield and other yielding components in Colocasia [41]. Although N is a necessary component for the synthesis of proteins and amino acids, a high concentration of nitrogen in the tissues can be hazardous to the plant's physiological and phonological responses [42]. Additionally, the mean values of N between sites change significantly, and the mean values of N between all seasons were significantly different.

Table 2. Seasonal and site wise variation in N, P and K (mg/Kg) of Soil ~~soil~~

Parameters	Seasons	Sites				Mean	C.D (P≤0.05)
		**Center**	**Inside**	**Outside**	**Control**		
**N**	Spring	300.33±2.43^{c}	283.40±2.74^{d}	256.73±2.06^{e}	139.27±3.13^{k}	**244.93^{b}**	Sites: 4.700<br>Seasons: 4.700<br>Sites × Seasons: 9.401
	Summer	320.49±1.80^{a}	307.15±0.65^{b}	282.34±1.95^{d}	146.47±1.16^{j}	**264.11^{a}**	
	Autumn	280.41±1.79^{d}	252.50±2.19^{f}	225.85±2.08^{h}	128.64±2.07^{l}	**221.85^{c}**	
	Winter	240.51±2.04^{g}	223.86±1.52^{h}	201.54±1.32^{i}	110.67±2.24^{m}	**194.14^{d}**	
	**Mean**	**285.43^{a}**	**266.73^{b}**	**241.61^{c}**	**131.26^{d}**		
**P**	Spring	71.29±1.12 ^{c}	57.42±0.89^{e}	43.95±1.71^{h}	25.39±1.08^{k}	**49.51^{b}**	Sites: 2.017<br>Seasons: 2.017<br>Sites × Seasons: 4.034
	Summer	87.36±0.85^{a}	73.44±1.87^{b}	54.68±1.24^{f}	28.45±2.01^{l}	**60.98^{a}**	
	Autumn	63.35±1.04^{d}	48.60±1.24^{g}	36.06±1.23^{j}	19.42±0.95^{m}	**41.86^{c}**	
	Winter	58.29±1.02^{e}	41.01±41.01^{i}	29.94±0.54^{k}	15.38±1.08^{n}	**36.15^{d}**	
	**Mean**	**70.07^{a}**	**55.12^{b}**	**41.16^{c}**	**22.16^{d}**		
**K**	Spring	275.70±0.38^{b}	251.77±0.97^{d}	237.70±1.08^{e}	125.35±1.03^{l}	**222.63^{b}**	Sites: 6.887<br>Seasons: 6.887<br>Sites × Seasons:13.774
	Summer	301.28±1.03^{a}	273.33±1.05^{c}	251.77±1.63^{d}	132.41±1.12^{k}	**239.70^{a}**	
	Autumn	253.38±1.12^{d}	234.29±0.90^{f}	218.64±1.12^{h}	110.35±0.94^{m}	**204.14^{c}**	
	Winter	232.49±1.07^{g}	210.03±1.03^{i}	195.28±0.95j	96.54±1.11^{n}	**183.58^{d}**	
	**Mean**	**265.71^{a}**	**242.35^{b}**	**225.85^{c}**	**116.16^{d}**		
Standard Limits (ICAR manual)		**N**<br>Low<107.1 mg/kg<br>Medium (107.1-214.2 mg/kg)<br>High >214.2 mg/kg		**P**<br>Low < 13.2mg/kg<br>Medium (13.2 – 33 mg/kg)<br>High > 33 mg/kg		K<br>Low < 49.10 mg/kg<br>Medium (49.10 -125 mg/kg)<br>High > 125 mg/kg	

N: Nitrogen, P: Phosphorus, K: Potassium

Formatted: Font: 11 pt
Formatted: Left
Formatted: Left
Formatted: Font: 11 pt, Not Bold, Font color: Auto
Formatted: Left
Formatted: Font: 11 pt

The movement of leachate from municipal solid waste (MSW) disposal can increase the phosphorus (P) content in soil at dumpsites. In the current study, soil phosphorus content at the experimental site ranged from 15.38 to 87.36 mg/kg. The highest mean P level was found at the center of the dumping site (70.07 mg/kg), followed by the inside (55.12 mg/kg) and outside (41.16 mg/kg) locations, with the lowest at the control site (22.16 mg/kg), as shown in Table 2. This elevated phosphorus level is likely due to the large quantities of organic waste, paper and cardboard, textiles, and plastics [43]. Seasonally, the highest mean phosphorus content was observed during the summer (60.98 mg/kg), followed by spring (49.51 mg/kg), autumn (41.85 mg/kg), and the lowest during winter (36.15 mg/kg). The concentration of P was higher in the soil samples of the dumping site than the controlled site. This may be explained by the fact that organic matter in waste contains sizable amounts of P, and that orthophosphate is released into the soil solution during mineralization. Additionally, humic substances and organic acids can be absorbed into soil surfaces, leading to depletion in ability of P adsorption by obstructing sites for the formation of complexes with Al, Fe, and Ca. Inorganic and organic products are also produced during the partial decomposition of organic waste [44]. Additionally, there are considerable differences in the mean values of P between locations and the mean values of P throughout all seasons.

Potassium readily dissolves in water; however, it remains largely intact in undisturbed soils. After being released from decomposing organic matter, potassium quickly and strongly binds to clay particles [32]. At the Achan dumping site, soil potassium levels ranged from 96.54

to 301.28 mg/kg. The highest concentration, 265.71 mg/kg, was found at the center of the dumping site, likely due to the decomposition of a substantial amount of kitchen waste. Conversely, the lowest concentration, 116.16 mg/kg, was observed at the control site, where there was less organic waste as given in Table 2. Among seasons, maximum K content reported during summer may be due to high decomposition rate of kitchen waste and less leaching and minimum content of it reported during winter may be due to high leaching losses by precipitation. Similar results were obtained by Deshmukh and Aher [30]. Despite the fact that K controls stomatal opening and shutting, cell enlargement, and other critical physiological activities. Numerous research have examined how much potassium affects plant growth, however high K concentrations in soil solutions restrict magnesium uptake, leading to magnesium deficit in plants [45]. Additionally, there are considerable differences in the mean values of K between sites as well as the mean values of K across all seasons.

Calcium (Ca) is crucial element in figuring out the soil's physical and chemical properties, especially its structure and pH. Clay platelets in soil colloids (caused by calcium ions) bind or aggregate to produce crumbs or peds. Acidity of the soil and exchangeable calcium levels are frequently correlated. In the pH range of 7.0 to 8.5, Ca is accessible. Exchangeable calcium levels in the soil are often low when the soil has an acidic pH [32]. During the present study, mean Ca concentration ranged from 668.33 to 984.39 (mg/Kg). Maximum Ca content reported during summer (913.52 mg/Kg) may be because of degradation of calcium rich waste such as bones and eggshells present in the kitchen waste and minimum (826.58 mg/kg) reported during winter may be because of low rate of degradation of waste. Among sites, maximum Ca content reported at center (957.67) of dumping site may be attributed to the clayey nature of soils of dumpsite as shown in Table 3. The mean values of Ca between all seasons were significantly

Formatted: Font: Not Bold

~~different; furthermore the mean values of Ca between sites also vary significantly.~~

During the present study, mean Ca concentration ranged from 668.33 to 984.39 (mg/Kg). Mean maximum concentration of Calcium among seasons was recorded in summer season (913.52 mg/kg), followed by Spring (893.44 mg/kg), autumn (856.92) and minimum was recorded in winter (826.58). Among sites mean maxium concentration of calcium was recorded in center (957.67 mg/kg), inside (941.24 mg/kg), outside (876.38 mg/kg) and minimum (712.91 mg/kg) was recorded at control. The mean values of Ca between all seasons were significantly different; furthermore the mean values of Ca between sites also vary significantly.

Formatted: Font: Not Bold

During the present study the mean Mg concentration ranged from 280.82 to 418.61 (mg/Kg). . Magnesium concentration was recorded maximum (385.09 mg/kg) during summer season, followed by spring (374.66 mg/kg), autumn (357.80 mg/kg) and minimum (349.58 mg/kg) was recorded in winter. Among sites maximum mean concentration of magnesium was recorded in center (402.42 mg/kg), followed by inside (396.72 mg/kg), outside (368.48 mg/kg) and minimum (299.49 mg/kg) was recorded at control site as depicted in Table 3. Additionally, there are considerable differences in the mean levels of Mg between locations as well as the mean values of Mg across all seasons.

Formatted: Font: Not Bold

Table 3. Seasonal and site wise variation in Ca and Mg (mg/kg) of soil

Parameters	Seasons	Sites				Mean	C.D (P≤0.05)
		Center	Inside	Outside	Control		
Ca	Spring	982.31±0.96^{c}	965.34±0.97^{d}	893.60±0.99^{i}	723.50±0.94^{n}	**893.44^{b}**	Sites:1.809<br>Seasons:1.809<br>Sites × Seasons:3.618
	Ssummer	997.74±1.13^{a}	984.39±0.90^{b}	913.43±0.93^{g}	758.51±0.88^{m}	**913.52^{a}**	
	Autumn	940.40±1.06^{e}	925.85±0.73^{f}	860.11±0.94^{l}	701.31±1.10^{o}	**856.92^{c}**	
	Winter	910.25±0.96^{h}	889.40±1.08^{j}	838.37±1.04^{k}	668.33±0.95^{p}	**826.58^{d}**	
	**Mean**	**957.67^{a}**	**941.24^{b}**	**876.38^{c}**	**712.91^{d}**		
Mg	Spring	412.86±0.71^{b}	405.61±1.35^{c}	376.28±0.81^{g}	303.89±1.25^{l}	**374.66^{b}**	Sites:3.873<br>Seasons:3.873<br>Sites × Seasons:7.745
	Ssummer	419.24±0.97^{a}	418.61±0.93^{a}	383.85±1.02^{f}	318.66±1.21^{k}	**385.09^{a}**	
	Autumn	395.12±0.77^{d}	388.99±0.90^{e}	352.50±1.27^{j}	294.61±1.13^{m}	**357.80^{c}**	
	Winter	382.47±0.95^{f}	373.67±0.94^{h}	361.38±1.03^{i}	280.82±1.17^{n}	**349.58^{d}**	
	**Mean**	**402.42^{a}**	**396.72^{b}**	**368.48^{c}**	**299.49^{d}**		
**Standard limits (ICAR manual)**		**Ca**<br>Low< 500 mg/kg<br>Medium (500-2500 mg/kg)<br>High > 2500 mg/kg		Mg<br>Sandy soils (51- 250 mg/kg)<br>Clayey soils (101-500 mg/kg)			

Ca: Calcium, Mg: Magnesium

Formatted: Left, Don't add space between paragraphs of the same style

Formatted: Don't add space between paragraphs of the same style

Formatted: Don't add space between paragraphs of the same style

489

Magnesium (Mg) is found in both relatively soluble forms and ionic form, Mg^{2+}, which is attached to the soil colloidal complex. Magnesium levels in acidic soils, especially sandy soils, are frequently rather low. Neutral soils usually contain more exchangeable magnesium [32]. During the present study the mean Mg concentration ranged from 280.82 to 418.61 (mg/Kg). Among sites maximum Mg concentration reported at center of dumping site (402.42 mg/kg) may be because of its less mobility due to presence of high organic waste and clayey nature of soil [46] and minimum (299.49) at control site may be because of the presence of less organic matter as given in Table 3. Among seasons maximum (385.09 mg/kg) Mg concentration reported during summer season may be because of less leaching and high rate of decomposition of organic waste and minimum (349.58 mg/kg) during winter may be attributed to high leaching loss due to the high positive water balance [46]. An excessive amount of magnesium in the soil inhibits plant growth because it reduces the roots' ability to reduce TTC (2, 3, 5-Triphenyl Tetrazolium chloride) because it inhibits the dehydrogenase enzyme linked to the respiratory systems [47]. Additionally, there are considerable differences in the mean levels of Mg between locations as well as the mean values of Mg across all seasons.

Micronutrients are essential for sustaining the fertility of the soil and crop productivity. Compared to macronutrients, micronutrients are required in smaller amounts. Even if the supply of macronutrients is balanced and high yielding varieties are cultivated, a lack of micronutrients will prevent maximum yields from being achieved [48]. Zinc (Zn) concentration in the soil in the current study ranged from 0.75 to 3.57 (mg/Kg) Table 4. The presence of this element in both

Formatted: Superscript

Formatted: Font: Not Bold

Formatted: Font: Not Bold

Formatted: Font: Not Bold

naturally occurring materials, such as yard wastes and food wastes, as well as man-made materials like pigments, inks, metals, and plastics may account for the highest mean Zn value of 2.92 mg/kg reported at the centre of the dumping site, while the lowest mean Zn value of 1.27 mg/kg was reported at the control site, as shown in Table 4. Because micronutrients are more tightly bonded to the soil at high pH than at low pH, the highest mean Zn concentration (reported as 2.89 mg/kg) during the winter season may be attributed to low pH, whereas the lowest mean Zn concentration (reported as 1.53 mg/kg) during the summer season may be attributed to high pH [49]. as shown in Fig 5. Although it serves as a nutrient for plants, zinc is hazardous to them at larger amounts. Zn toxicity in plants results in growth inhibition, a reduction in biomass production, competition for nutrients, inactivation of enzymes, removal of vital components from functional locations, chlorosis, and, in certain situations, blockage of Fe translocation [50]. The mean values of Zn between spring, summer and autumn were significantly different, while as no significant difference was reported between autumn and winter. However the mean values of all sites were significantly different.

Zinc (Zn) concentration in the soil in the current study ranged from 0.75 to 3.57 (mg/Kg). Among seasons maximum mean concentration of Zn (**2.89 mg/kg**) was recorded in winter season, followed by autumn (**2.54 mg/kg**), spring (1.75 mg/kg) and minimum was recorded in summer (1.53 mg/kg). Among sites maximum mean concentration of Zn was recorded at center (2.92 mg/kg), followed by inside (2.50 mg/kg), outside (2.03 mg/kg) and minimum (1.27 mg/kg) was recorded at control. The mean values of Zn between spring, summer and autumn were significantly different, while as no significant difference was reported between autumn and winter. However the mean values of all sites were significantly different.

The mean maximum Fe in the current study ranged from 1.51 to 12.51 (mg/Kg). For iron

(Fe) maximum mean concentration among seasons was recorded in winter (8.78 mg/kg), followed by autumn (8.26 mg/kg), spring (5.48 mg/kg) and minimum (4.65 mg/kg) was recorded in summer, while as among sites maximum mean concentration was recorded at center (10.56 mg/kg), followed by inside (6.52 mg/kg), outside (6.37 mg/kg) and minimum (3.71 mg/kg) was recorded at control. The mean values of Fe between all seasons were significantly different. Among sites the values of Fe were significantly different between center, inside and control, however no significant difference was reported between inside and outside dumpsite.

During the current study, mean concentration of Cu ranged from 0.17 to 2.72 (mg/Kg). Copper (Cu) was recorded maximum among seasons in winter (2.11 mg/kg), followed by autumn (1.97 mg/kg), spring (1.19 mg/kg) and minimum was recorded in summer (0.88 mg/kg). Among sites maximum mean concentration of Cu was recorded in center (2.07 mg/kg), followed by inside (1.80 mg/kg), outside (1.74 mg/kg) and minimum was recorded at control (0.54 mg/kg). The mean values of Cu were significantly different throughout all seasons, and it was also observed that these differences were significant between sites.

During the present study, mean Manganese (Mn) concentration of soils ranged from 4.54 to 11.83 (mg/Kg) shown in Table 4. Manganese was recorded maximum in winter (8.92 mg/kg), followed by autumn (8.38 mg/kg), spring (7.62 mg/kg) and minimum was recorded in summer (7.24 mg/kg). Among sites maximum mean concentration was recorded at center (10.73 mg/kg), followed by inside (9.34 mg/kg), outside (7.42 mg/kg) and minimum was recorded at control (4.68 mg/kg) as depicted in Table 4. The mean Mn values also varied significantly between different locations and across all seasons.

Formatted: Indent: First line: 0"

Table 4: Seasonal and site wise variation in micronutrients, viz Zn, Fe, Cu and Mn (mg/kg) of soil

Parameters	Seasons	Sites: Center	Inside	Outside	Control	Mean	C.D (P≤0.05)
Zn	Spring	2.39 ± 0.14^{de}	2.01 ± 0.31^{ef}	1.55 ± 0.33^{gh}	1.07 ± 0.28^{ij}	**1.75^{b}**	Sites:0.212<br>Seasons:0.212<br>Sites × Seasons:0.424
	Summer	2.34 ± 0.52^{de}	1.76 ± 0.01^{fg}	1.30 ± 0.04^{hi}	0.75 ± 0.32^{j}	**1.53^{c}**	
	Autumn	3.36 ± 0.21^{ab}	2.99 ± 0.09^{bc}	2.53 ± 0.17^{d}	1.27 ± 0.12^{hi}	**2.54^{a}**	
	Winter	3.57 ± 0.29^{a}	3.26 ± 0.15^{ab}	2.74 ± 0.24^{cd}	1.98 ± 0.24^{ef}	**2.89^{a}**	
	**Mean**	**2.92^{a}**	**2.50^{b}**	**2.03^{c}**	**1.27^{d}**		
Fe	Spring	9.25 ± 0.29^{b}	5.21 ± 0.15^{f}	5.14 ± 0.32^{f}	2.31 ± 0.14^{h}	**5.48^{c}**	Sites:0.250<br>Seasons:0.250<br>Sites × Seasons:0.50
	Summer	8.45 ± 0.21^{c}	4.41 ± 0.24^{g}	4.24 ± 0.23^{g}	1.51 ± 0.18^{i}	**4.65^{d}**	
	Autumn	12.05 ± 0.17^{a}	8.01 ± 0.27^{cd}	7.85 ± 0.11^{d}	5.13 ± 0.19^{f}	**8.26^{b}**	
	Winter	12.51 ± 0.89^{a}	8.46 ± 0.15^{c}	8.27 ± 0.18^{cd}	5.88 ± 0.06^{e}	**8.78^{a}**	
	**Mean**	**10.56^{a}**	**6.52^{b}**	**6.37^{b}**	**3.71^{c}**		
Cu	Spring	1.62 ± 0.24^{d}	1.36 ± 0.09^{e}	1.29 ± 0.13^{e}	0.49 ± 0.12^{h}	**1.19^{c}**	Sites:0.117<br>Seasons:0.117<br>Sites × Seasons: 0.235
	Summer	1.33 ± 0.11^{e}	1.04 ± 0.08^{f}	0.99 ± 0.11^{f}	0.17 ± 0.06^{i}	**0.88^{d}**	
	Autumn	2.61 ± 0.24^{ab}	2.33 ± 0.15^{c}	2.28 ± 0.18^{c}	0.65 ± 0.05^{gh}	**1.97^{b}**	
	Winter	2.72 ± 0.06^{a}	2.46 ± 0.07^{bc}	2.39 ± 0.19^{bc}	0.87 ± 0.10^{fg}	**2.11^{a}**	
	**Mean**	**2.07^{a}**	**1.80^{b}**	**1.74^{b}**	**0.54^{c}**		
Mn	Spring	10.21 ± 0.63^{cd}	8.79 ± 0.25^{f}	6.89 ± 0.39^{i}	4.58 ± 0.17^{j}	**7.62^{c}**	Sites: 0.212<br>Seasons: 0.212<br>Sites × Seasons: 0.425
	Summer	9.73 ± 0.04^{e}	8.32 ± 0.28^{g}	6.47 ± 0.08^{i}	4.54 ± 0.05^{j}	**7.24^{d}**	
	Autumn	11.18 ± 0.23^{b}	$9.83\pm0.11de$	7.81 ± 0.39^{h}	4.69 ± 0.06^{j}	**8.38^{b}**	
	Winter	11.83 ± 0.06^{a}	10.42 ± 0.09^{c}	8.53 ± 0.21^{fg}	4.92 ± 0.05^{j}	**8.92^{a}**	
	**Mean**	**10.73^{a}**	**9.34^{b}**	**7.42^{c}**	**4.68^{d}**		
Standard limits (ICAR manual)	**Zn**<br>Low < 0.5 mg/kg<br>Medium (1 – 3 mg/kg)<br>High > 5 mg/kg		**Fe**<br>Low < 2 mg/kg<br>Medium (4 -6mg/kg)<br>High > 10 mg/kg		**Cu**<br>Low < 0.1 mg/kg<br>Medium (0.3 -0.8mg/kg)<br>High > 3 mg/kg		Mn<br>Low< 0.5 mg/kg<br>Medium (1.2 -2.5 mg/kg)<br>High > 6 mg/kg

Formatted Table

Formatted: Left, Don't add space between paragraphs of the same style

Formatted: Don't add space between paragraphs of the same style

Formatted: Don't add space between paragraphs of the same style

Formatted: Left, Don't add space between paragraphs of the same style

Zn: Zinc, Fe: Iron, Cu: Copper, Mn: Manganese

Ferric oxide (Fe_2O_3), also known as hematite, is the most prevalent form of Iron (Fe) in soils. It is exceedingly insoluble and gives soil its red colour. Commonly, the oxide form is hydrated [51]. The mean maximum Fe in the current study ranged from 1.51 to 12.51 (mg/Kg). Because Fe occurs in the reduced state (Fe++)

in the soil solution or is adsorbed onto the surface of soil particle surfaces in soils rich in organic matter, the greatest (10.56 mg/kg) Fe concentration found at the centre of the dumping site may be attributed to the presence of organic matter in soils [51]. The maximum iron concentration reported during winter season (8.78 mg/kg) depicted in table 4 and Fig 6 may be attributed to low pH, because with increase in pH, ferrous ion (Fe^{2+}) gets converted to ferric ion (Fe^{3+}). Low solubility in solution makes the ferric ion (Fe^{3+}) compounds less bioavailable, and a minimum of 4.65 mg/kg was

Formatted: Subscript

Formatted: Subscript

Formatted: Font: Not Bold

Formatted: Superscript

recorded during the summer [52]. The root cells and plasma membrane are damaged by the extra iron ions, which results in oxidative stress and increased formation of reactive oxygen species (ROS). Root cells may die as a result of ROS harming cellular components such lipids, proteins, and DNA [53]. Furthermore it, can also affect the uptake of other essential elements, such as phosphorus and zinc, by competing with the transporters that facilitate their uptake by the roots. This interference can lead to nutrient imbalances, reduced growth, and overall plant health [54]. The mean values of Fe between all seasons were significantly different. Among sites the values of Fe were significantly different between center, inside and control, however no significant difference was reported between inside and outside dumpsite.

Compared to other trace metals, copper (Cu) has extremely little mobility because it is firmly adsorbed onto soil particles. Due to this reduced mobility, Cu has a tendency to concentrate on soil particles. Leaching can happen when the amount of Cu in the soil exceeds the capacity of the soil type to hold the copper ions. The pH, cation exchange capacity, organic matter content, and presence of iron, manganese, and aluminium oxides all affect how much Cu is present in soils. The amount of water in the soil affects the ability of the soil to store copper through biotic and abiotic oxidation-reduction reactions [32]. During the current study, mean concentration of Cu ranged from 0.17 to 2.72 (mg/Kg). Among sites mean maximum (2.07 mg/kg) concentration reported at center of dumping site may be due to the higher stability constants of Cu complexes with organic matter and minimum (0.54) was reported at control site as shown in Table 4. Among seasons, maximum Cu concentration reported during winter season (2.11mg/kg) may be because of low pH, as at

Formatted: Indent: First line: 0"

low pH Cu compounds are more soluble and minimum concentration (0.88 mg/kg) of Cu reported during summer may be because of high pH, as high pH results in precipitation of copper (II) hydroxide (Cu $(OH)_2$) and Copper(I) hydroxide (CuOH) [55] as shown in Fig 7. All phases of plant growth are affected by high Cu concentration toxicity, which has considerable detrimental impacts on morphology, physiological function, and molecular level [56]. .Additionally, too much copper in the soil inhibits the activity of -amylase and invertase isoenzymes, which limits the ability of many plant species to break down reserve food supplies like starch and sucrose [57]. The mean values of Cu were significantly different throughout all seasons, and it was also observed that these differences were significant between sites.

During the present study, mean Manganese (Mn) concentration of soils ranged from 4.54 to 11.83 (mg/Kg) shown in Table 4. Maximum (10.73 mg/kg) concentration of Mn reported at center of dumping site may be due to decomposition of plants and animals waste as well as animal excrement [58]. The lowest (4.68 mg/kg) concentration of Mn was reported at control site as given in Table 4.

The highest Mn concentration was observed during the winter season (8.92 mg/kg), likely due to a decrease in soil pH. At low pH, trace elements are typically more soluble because of high desorption and low adsorption [32]. Conversely, the lowest Mn concentration (7.24 mg/kg) was reported during the summer, attributed to increased adsorption [59], as shown in Fig 8. Although excessive Mn accumulation in leaves can cause toxicity and reduce crop yield, manganese is

essential for terrestrial plants. The mean Mn values also varied significantly between different locations and across all seasons.

Heavy metals are significant pollutants that enter the environment through various channels, including landfill leachate, solid waste, and wastewater. According to Radfard et al. [60] and Kusin et al. [61], heavy metals in leachate can contaminate soil and subsurface water supplies. As these contaminants move up the food chain, they can impact human health and cause various ailments. Leachate is a critical source of pollution, releasing chemicals into soil and water sources, and its toxicity to human cells highlights its environmental impact [62]. In the present study the concentration of lead (Pb) ranged from 0.98mg/kg to 98.3 mg/kg. Among seasons max concentration (72.1 mg/kg) was reported during summer season followed by autumn (68.5 mg/kg), spring (59.08 mg/kg) and min during winter (35.9). Among sites maximum concentration (85.02 mg/kg) was reported at center of dumpsite, followed by inside (76.02 mg/kg), outside (68.72 mg/kg) and minimum was reported at control site (5.90mg/kg) as depicted in Fig 9.The values of Pb are higher than permissible limit (85mg/kg) at center during spring, summer and autumn, Inside during summer and autumn and outside during summer season. Lead-acid batteries used in automobiles, rechargeable nickel-cadmium batteries, consumer electronics, glass, ceramics, and plastic products including PVC resins are sources of Pb in landfill leachate. Other sources of lead include soldered cans, pigments, brass and bronze products, rubber goods, spent motor oil, and wine bottle wrappers made of lead foil. Additionally to the fact that the mean Pb values between sites varied greatly, these mean values of Pb between seasons varied significantly as well.

Formatted: Indent: First line: 0", Pattern: Clear

In the present study the concentration of lead (Pb) ranged from 0.98mg/kg to 98.3 mg/kg. The mean maximum concentration of lead (Pb) among seasons was recorded in summer (72.1 mg/kg), followed by autumn (68.5 mg/kg), spring (59.08 mg/kg) and minimum was recorded in winter (35.9 mg/kg) while as among sites maximum mean concentration of Pb was recorded at center (85.02 mg/kg), followed by inside (76.02 mg/kg), outside (68.72 mg/kg) and minimum (5.90 mg/kg) was recorded at control. Additionally to the fact that the mean Pb values between sites varied greatly, these mean values of Pb between seasons varied significantly as well.

The concentration of cadmium Cd ranged from 0.06mg/kg to 5.89 mg/kg. Cadmium (Cd) was recorded maximum in summer season (4.46 mg/kg) , followed by autumn (3.80 mg/kg), spring (3.22 mg/kg) and minimum (1.57 mg/kg) was recorded in winter and among maximum mean concentration of Cd was recorded at center (4.56 mg/kg), followed by inside (4.26 mg/kg), outside (4.08 mg/kg) and minimum was recorded at control (0.15 mg/kg). Among the sites, the mean cadmium values were significantly different between the center and control, and inside and control; however, no significant difference was reported between outside and control.

The concentration of Nickel (Ni) ranged from 0.65mg/kg to 42.10 mg/kg. Ni was recorded maximum in summer (29.70 mg/kg), followed by autumn (22.23 mg/kg), spring (17.01 mg/kg) and minimum was recorded in winter (9.30 mg/kg). In case of sites it was recorded maximum at center (29.04 mg/kg), followed by inside (25.86 mg/kg), outside () and minimum (21.88 mg/kg) was recorded at control (1.47 mg/kg) as depicted in Table 5. The mean values of Ni between seasons as well as sites differ significantly.

The concentration of chromium (Cr) ranged from 2.31 mg/kg to 30.40 mg/kg. Among seasons max concentration (22.41 mg/kg) was reported during summer season followed by autumn (19.67mg/kg), spring (15.37 mg/kg) and min during winter (9.88

mg/kg). Among sites maximum concentration (23.37 mg/kg) was reported at center of dumpsite, followed by inside (21.12 mg/kg), outside (18.61 mg/kg) and minimum was reported at control site (4.23mg/kg). The mean values of Cr between seasons were reported to be significantly different. Among sites the mean values of Cr between inside, outside, control were significantly different; however no significant difference in mean values of Cr was reported between center and inside.

The value of Arsenic (As) ranged from 0.23 mg/kg to 18.50 mg/kg. Among seasons max concentration (12.63 mg/kg) was reported during summer season followed by autumn (9.86 mg/kg), spring (8.04 mg/kg) and min during winter (5.63 mg/kg). Among sites maximum concentration (14.10 mg/kg) was reported at center of dumpsite, followed by inside (11.53 mg/kg), outside (9.87 mg/kg) and minimum was reported at control site (0.66mg/kg) as depicted in Table 6. The mean values of As between seasons were significantly different. In addition to this the mean values of As between all sites was also reported to be significantly different. Furthermore the correlation between the recorded paramaters is depicted in Table 7.

Table 5. Seasonal and site wise variation in Pb, Cd and Ni (mg/Kg) of soil

Parameters	Seasons	Sites				Mean	C.D (P≤0.05)
		Center	Inside	Outside	Control		
**Pb**	Spring	89.1±3.34^{b}	76.4±1.62^{c}	65.8±2.44^{d}	5.03±0.58^{hi}	**59.08^{c}**	Sites: 1.799<br>Seasons: 1.799<br>Sites × Seasons: 3.557
	Summer	98.3±4.05^{a}	92.5±2.53^{ab}	87.4±2.25^{b}	10.2±1.22^{h}	**72.1^{a}**	
	Autumn	96.8±1.69^{a}	88.6±2.09^{b}	81.4±1.56^{c}	7.4±0.90^{h}	**68.5^{b}**	
	Winter	55.9±1.41^{e}	46.6±2.00^{f}	40.3±1.83^{g}	0.98±0.07^{i}	**35.9^{d}**	
	**Mean**	**85.02^{a}**	**76.02^{b}**	**68.72^{c}**	**5.90^{d}**		
**Cd**	Spring	4.69±1.08^{a}	4.12±1.00^{ab}	3.98±0.85^{ab}	0.12±0.02^{cd}	3.22^{b}	Sites: 1.10<br>Seasons:1.10<br>Sites × Seasons: 2.20
	Summer	6.11±1.28^{a}	5.89±0.76^{a}	5.61±0.66^{a}	0.25±0.08^{cd}	4.46^{a}	
	Autumn	5.11±1.23^{a}	5.01±0.50^{a}	4.89±1.28^{a}	0.19±0.03^{cd}	3.80^{ab}	
	Winter	2.34±0.57^{bc}	2.02±0.43^{bcd}	1.87±0.52^{bcd}	0.06±0.32^{d}	1.57^{c}	
	**Mean**	4.56^{a}	4.26^{a}	4.08 ^{a}	0.15^{b}		
**Ni**	Spring	26.20±1.02^{de}	22.50±2.15^{ef}	18.30±1.45^{fg}	1.06±0.24^{i}	17.01^{c}	Sites: 2.047<br>Seasons: 2.047<br>Sites × Seasons:4.093
	Summer	42.10±3.02^{a}	38.70±4.33^{ab}	35.70±2.80^{bc}	2.32±0.46^{i}	29.70^{a}	
	Autumn	32.40±2.11^{c}	29.80±3.51^{cd}	24.90±1.96^{de}	1.85±0.19^{i}	22.23^{b}	
	Winter	15.47±2.17^{g}	12.46±1.64^{gh}	8.65±1.10^{h}	0.65±0.18^{i}	9.30^{d}	
	**Mean**	29.04^{a}	25.86^{b}	21.88^{c}	1.47^{d}		
Permissible value of soil (mg/kg)<br>Pb=85; Cd=0.8 ; Ni=35				Permissible value of plant<br>Pb=2; Cd=0.02 ; Ni=10		Permissible value of water<br>Pb=0.05; Cd=0.005 ; Ni=0.07	

Pb: Lead, Cd: Cadmium, Ni: Nickel

The concentration of cadmium Cd ranged from 0.06mg/kg to 5.89 mg/kg. Among seasons max concentration (4.46 mg/kg) was reported during summer season followed by autumn (3.80mg/kg), spring (3.22 mg/kg) and min during winter (1.57). Among sites maximum concentration (4.50 mg/kg) was reported at center of dumpsite, followed by inside (4.21 mg/kg), outside (4.19 mg/kg) and minimum was reported at control site (0.15mg/kg) as depicted in Fig 10.

Cadmium (Cd) levels at all sites exceeded the permissible limit of 0.8 mg/kg across all seasons. The sources of cadmium in waste include nickel-cadmium batteries, pigments, stabilizers in plastics (primarily PVC), consumer electronics (notably the steel chassis of vintage TVs and radios that were cadmium-plated to prevent corrosion), antique appliances with cadmium-plated parts, textile dyes and paints, glass and ceramics, and pigments used in non-newspaper printing inks. Cadmium is one of the most ecotoxic metals, causing detrimental effects on soil health, plant metabolism, and the health of humans and animals [63]. Even at very low concentrations, chronic exposure to cadmium can lead to anemia, anosmia, cardiovascular diseases, and renal problems [64]. Significant differences in mean cadmium values were observed between seasons. Among the sites, the mean cadmium values were significantly different between the center and control, and inside and control; however, no significant difference was reported between outside and control.

The concentration of Nickel (Ni) ranged from 0.65mg/kg to 42.10 mg/kg. Among seasons max concentration (29.70 mg/kg) was reported during summer season followed by autumn (22.23mg/kg), spring (17.01 mg/kg) and min during winter (9.30). Among sites maximum concentration (29.04 mg/kg) was reported at center of dumpsite, followed by inside (25.86

mg/kg), outside (21.88 mg/kg) and minimum was reported at control site (1.47mg/kg) as depicted in Table 5, Fig 11. The values of Ni were higher than the permissible limits (35 mg/kg) during summer season at all sites. The values were lower than the permissible limits during other seasons. The sources of Ni are lead cadmium batteries, electroplating materials, clinical wastes [65]. Because nickel is non-biodegradable and has the potential to accumulate in the food web, it is responsible for a number of health issues in humans, including allergic skin, headache, vertigo, nausea, vomiting, insomnia, and irritability [65, 66]. The mean values of Ni between seasons as well as sites differ significantly.

The concentration of chromium (Cr) ranged from 2.31 mg/kg to 30.40 mg/kg. Among seasons max concentration (22.41 mg/kg) was reported during summer season followed by autumn (19.67mg/kg), spring (15.37 mg/kg) and min during winter (9.88 mg/kg). Among sites maximum concentration (23.37 mg/kg) was reported at center of dumpsite, followed by inside (21.12 mg/kg), outside (18.61 mg/kg) and minimum was reported at control site (4.23mg/kg) as depicted in Table 6 and Fig 12. The sources of Cr are electroplating and Cr containing wastes. The concentration of Cr was less than permissible value at all sites. The sorption properties of the soil, such as its clay, iron oxide, and organic matter contents, influence the mobility of Cr. Surface runoff can carry chromium to surface waters in both its soluble and precipitated forms. Chromium compounds, both soluble and unadsorbable, can seep into groundwater from the soil. As soil pH rises, Cr (VI) has a greater propensity to leach. However, the majority of Cr discharged into natural rivers is particle associated and eventually deposits into the sediment [67]. Human allergic dermatitis and chromium are related [68]. The mean values of Cr between seasons were reported to be significantly different. Among sites the mean values of Cr between inside, outside, control were significantly different; however no significant difference in mean

~~values of Cr was reported between center and inside.~~

Table 6. Seasonal and site wise variation in Cr, and As (mg/Kg) of soil

Parameters	Seasons	Sites				Mean	C.D (P≤0.05)
		Center	Inside	Outside	Control		
Cr	Spring	21.80±1.52^{cd}	19.60±1.62^{de}	16.70±1.49^{ef}	3.41±0.63^{i}	**15.37^{c}**	Sites: 1.43<br>Seasons: 1.43<br>Sites × Seasons: 2.86
	Summer	30.40±3.25^{a}	27.60±1.58^{ab}	25.30±2.01^{bc}	6.34±0.72^{hi}	**22.41^{a}**	
	Autumn	26.10±1.21^{abc}	24.90±1.95^{bc}	22.80±2.52^{cd}	4.89±1.03^{i}	**19.67^{b}**	
	Winter	15.20±1.47^{ef}	12.40±1.54^{fg}	9.64±0.41^{gh}	2.31±0.26^{i}	**9.88^{d}**	
	**Mean**	**23.37^{a}**	**21.12^{a}**	**18.61^{b}**	**4.23^{c}**		
As	Spring	13.40±1.12^{bcd}	9.65±0.65^{ef}	8.67±0.42^{ef}	0.45±0.07^{h}	**8.04^{c}**	Sites: 1.31<br>Seasons: 1.31<br>Sites × Seasons:2.62
	Summer	18.50±1.47^{a}	16.30±1.76^{ab}	14.60±1.45^{bc}	1.12±0.12^{h}	**12.63^{a}**	
	Autumn	15.30±1.58^{bc}	12.70±1.26^{cd}	10.60±1.15^{de}	0.86±0.05^{h}	**9.86^{b}**	
	Winter	9.21±0.79^{ef}	7.48±0.68^{fg}	5.62±1.10^{g}	0.23±0.07^{h}	**5.63 ^{d}**	
	**Mean**	**14.10^{a}**	**11.53^{b}**	**9.87^{c}**	**0.66^{d}**		

Permissible value of soil (mg/kg)	Permissible value of plant (mg/kg)	Permissible value of Water (mg/kg)
Cr= 100 ;<br>As=20	Cr= 1.30<br>As=0.05	Cr: 0.05<br>As: 0.05

Cr: Chromium, As: Arsenic

The value of Arsenic (As) ranged from 0.23 mg/kg to 18.50 mg/kg. Among seasons max concentration (12.63 mg/kg) was reported during summer season followed by autumn (9.86 mg/kg), spring (8.04 mg/kg) and min during winter (5.63 mg/kg). Among sites maximum concentration (14.10 mg/kg) was reported at center of dumpsite, followed by inside (11.53 mg/kg), outside (9.87 mg/kg) and minimum was reported at control site (0.66mg/kg) as depicted in table 6, Fig 13. The permissible limit of As is soil is 20 mg/kg. The mean values of As between seasons were significantly different. In addition to this the mean values of As between all sites was also reported to be significantly different. The concentration of heavy metals, specifically Pb, Cd, Ni, Cr, and As, is higher during the summer than it is during other seasons. The leaching of heavy metals is also higher during the summer because temperature influences it; as the temperature rises, the leaching of heavy metals also increases. However, the minimum concentration of heavy metals during winter season might be attributed to runoff effect during winter season which facilitates the leaching of heavy metals from soil and contributes to the dilution of soil solution during winter season.

Formatted: Indent: First line: 0"

Formatted: Indent: First line: 0"

Formatted: Section start: Continuous, Width: 11", Height: 8.5"

Table 7: Correlation between various physico-chemical paramaters of soil

	pH	EC	MC	N	P	K	Ca	Mg	Zn	Fe	Cu	Mn	Pb	Cd	Ni	Cr	As
pH	1																
EC	-0.86	1															
MC	-0.48	0.78	1														
N	-0.87	0.81	0.61	1													
P	-0.92	0.92	0.60	0.93	1												
K	-0.86	0.82	0.65	0.99	0.92	1											
Ca	-0.88	0.84	0.67	0.98	0.92	0.98	1										
Mg	-0.87	0.84	0.67	0.98	0.92	0.98	0.99	1									
Zn	-0.36	0.57	0.85	0.37	0.34	0.43	0.45	0.45	1								
Fe	-0.39	0.64	0.86	0.40	0.41	0.46	0.45	0.45	0.93	1							
Cu	-0.33	0.46	0.80	0.39	0.28	0.46	0.47	0.46	0.95	0.88	1						

Formatted: Font: Bold
Formatted: Font: Bold
Formatted Table
Formatted: Font: 10 pt
Formatted: Line spacing: single
Formatted: Line spacing: single
Formatted: Font: 10 pt
Formatted: Line spacing: single
Formatted: Font: 10 pt
Formatted: Line spacing: single
Formatted: Font: 10 pt
Formatted: Line spacing: single
Formatted: Font: 10 pt
Formatted: Line spacing: single
Formatted: Font: 10 pt
Formatted: Font: 10 pt
Formatted: Line spacing: single
Formatted: Font: 10 pt
Formatted: Line spacing: single
Formatted: Font: 10 pt
Formatted: Line spacing: single
Formatted: Font: 10 pt
Formatted: Line spacing: single
Formatted: Font: 10 pt
Formatted: Line spacing: single
Formatted: Font: 10 pt
Formatted: Line spacing: single
Formatted: Font: 10 pt

Mn	-0.68	0.84	0.93	0.71	0.69	0.75	0.78	0.78	0.88	0.86	0.85	1					
Pb	-0.87	0.75	0.56	0.95	0.86	0.95	0.93	0.92	0.43	0.46	0.48	0.70	1				
Cd	-0.84	0.67	0.43	0.93	0.83	0.92	0.89	0.87	0.31	0.33	0.36	0.57	0.98	1			
Ni	-0.89	0.71	0.41	0.92	0.88	0.91	0.87	0.86	0.29	0.33	0.30	0.57	0.95	0.98	1		
Cr	-0.90	0.73	0.45	0.93	0.87	0.92	0.90	0.88	0.35	0.38	0.38	0.62	0.98	0.98	0.98	1	
As	-0.90	0.79	0.54	0.95	0.91	0.95	0.92	0.91	0.41	0.45	0.42	0.69	0.97	0.96	0.98	0.98	1

Formatted: Font: 10 pt
Formatted: Line spacing: single
Formatted: Line spacing: single
Formatted: Font: 10 pt
Formatted: Line spacing: single
Formatted: Font: 10 pt
Formatted: Font: 10 pt
Formatted: Line spacing: single
Formatted: Line spacing: single
Formatted: Font: 10 pt
Formatted: Line spacing: single
Formatted: Font: 10 pt
Formatted: Indent: First line: 0"

Discussion

Soil pH determines the acidity or alkalinity of soil solutions and refers to the soil's hydrogen ion content. Natural soil typically has a pH between 7 and 8.5, although this range can vary based on biological activity, temperature, and municipal waste disposal practices [28]. Soil pH influences the availability of essential plant nutrients and the concentration of toxic elements, thereby affecting plant growth [29]. As it impacts the availability of all nutrients in the soil it has a direct effect on the survival and development of plants. In this, study pH of dumpsite soils ranged from 6.1 to 6.9 throughout study period. The mean pH values ranged from slightly acidic to neutral, which may be due to the decomposition of organic matter leading to the formation of organic acids. However, the pH increased with increase in distance from dumpsite which may be attributed to release of less organic acids away from dumpsite. Further, the lowest pH value reported during summer season may be attributed to more degradation of organic matter leading to increase in production of organic acids because of increased microbial activity and favorable temperature and moisture conditions during summer season as compared to winter season as depicted in Fig 2.

Fig 2: Graphical representation of pH among different sites and seasons. S1 is spring, S2 is summer, S3 is autumn and S4 is winter, while as C1 is center, C2 is inside, C3 is outside and C4 is Control.

The pH value of control site (Shalimar) soil was higher than the other three sites during all the seasons, this may be due to presence of less organic matter and hence less microbial activity. The slightly acidic pH of the soils at three other sites suggests that solid waste contributed to soil acidity, leading to a decrease in the availability of essential plant nutrients such as phosphorus

Formatted: Font: 18 pt, Bold

Formatted: Indent: First line: 0"

Formatted: Left, Space Before: 0 pt, After: 10 pt, Line spacing: Multiple 1.15 li

Formatted: Font: (Default) +Body (Calibri), 11 pt, Font color: Auto

and molybdenum, while increasing the availability of toxic elements, particularly aluminum and manganese [30].

Formatted: Font:

Electrical conductivity is an indirect measurement that is correlated with a number of the physical and chemical characteristics of soil. EC is affected by moisture content that soil particles hold. The total quantity of anions and cations determines electrical conductivity. It also depends on the primary salts in the soil solution, which have a negative impact on the qualities of the soil [31]. The optimal EC is crop specific, and depends on environmental conditions [32]. In general, higher EC hinders nutrient uptake by increasing the osmotic pressure of the nutrient solution, and the increased discharged of nutrients into the environment, resulting in environmental pollution. Lower EC may severely affect plant health and yield [33, 34]. In the current study, maximum mean value of EC was reported at center of dumping site, which may be due to increased concentration of cations and anions within the dumpsite soil [35] and minimum was reported at control site. Among seasons maximum mean EC value was reported during summer season, which may be due to increase in degradation of salts and release of large number of cations and anions favoured by temperature and minimum was reported during winter due to decrease in degradation of organic matter as shown in Table 1 and Fig 3.

Fig 3: Graphical representation of EC (dS/m) among different sites and seasons. S1 is spring, S2 is summer, S3 is autumn and S4 is winter, while as C1 is center, C2 is inside, C3 is outside and C4 is Control

Formatted: Normal, Space Before: 6 pt, After: 6 pt, Line spacing: 1.5 lines, No widow/orphan control, Pattern: Clear, Tab stops: 0.49", Left

The electrical conductivity of the soil samples collected within the dumpsite was high compared to the soil sample collected from the control site.

Moisture content refers to the amount of water soil can hold. Most soil samples from municipal

solid waste have moisture content ranging from 15% to 40% [31]. The highest mean moisture content was observed at the center of the dumping site, which may be because dumpsite soil retains more moisture due to the natural ground being covered by municipal solid waste, which has a high moisture content and prevents soil moisture from evaporating directly [36]. The mean moisture content of soil decreased with the increase of the distance from the dumpsite. This higher moisture content in the dumpsite soil may be associated with the presence of high organic matter which has capacity to hold more water [37]. This excess amount of water suffocates plant roots by reducing the amount of oxygen available to them. Without enough oxygen, roots are not able to absorb nutrients and water effectively, which in turn inhibits the growth of plants. The moisture content was high during winter season which may be attributed to the high precipitation rate during winter.

Nitrogen (N) is the most important ingredient that plants take up from the soil. In the present study, the concentration of N was maximum at center of dumping site during all the seasons as shown in Table 2. The samples within the dumpsite have high N concentration than the samples away from dumpsite, which may be attributed to abundant amount of the putrescible waste (biowaste, kitchen waste) [38]. The maximum N concentration observed during summer season may be due to increase in reaction rate because of high temperature. Additionally, the high levels of organic matter present in dump soil may be responsible for the high N content [39]. It has been reported that application of N significantly increases tuber yield and other yielding components in Colocasia [40]. Although N is a necessary component for the synthesis of proteins and amino acids, a high concentration of nitrogen in the tissues can be hazardous to the plant's physiological and phonological responses [41].

The movement of leachate from municipal solid waste (MSW) disposal site can increase

the phosphorus (P) content in soil at dumpsites. In the current study, soil phosphorus content at the experimental site ranged from 15.38 to 87.36 mg/kg. In the current study Tthe highest mean P level was found at the center of the dumping site (70.07 mg/kg), followed by the inside (55.12 mg/kg) and outside (41.16 mg/kg) locations, with the lowest at the control site (22.16 mg/kg), as shown in Table 2. This elevated phosphorus level is likely due which may be attributed to the large quantities of organic waste, paper and cardboard, textiles, and plastics [4243]. Seasonally, the highest mean phosphorus content was observed during the summer (60.98 mg/kg), followed by spring (49.51 mg/kg), autumn (41.85 mg/kg), and the lowest during winter (36.15 mg/kg). The concentration of P was higher in the soil samples of the dumping site than the controlled site. This may be explained by the fact that organic matter in waste contains sizable amounts of P, and that orthophosphate is released into the soil solution during mineralization. Additionally, humic substances and organic acids can be absorbed into soil surfaces, leading to depletion in ability of P adsorption by obstructing sites for the formation of complexes with Al, Fe, and Ca. Inorganic and organic products are also produced during the partial decomposition of organic waste [434]. Additionally, there are considerable differences in the mean values of P between locations and the mean values of P throughout all seasons.

-Potassium readily dissolves in water; however, it remains largely intact in undisturbed soils. After being released from decomposing organic matter, potassium quickly and strongly binds to clay particles [312]. At the Achan dumping site, soil potassium levels ranged from 96.54 to 301.28 mg/kg. In this study Tthe highest concentration of potassium , 265.71 mg/kg, was found at the center of the dumping site, likely due to the decomposition of a substantial amount of kitchen waste. Conversely, the lowest concentration, 116.16 mg/kg, was observed at the control site, where there was less organic waste as given in Table 2. Among seasons, maximum

K content reported during summer may be due to high decomposition rate of kitchen waste and less leaching and minimum content of it reported during winter may be due to high leaching losses by precipitation. Similar results were obtained by Deshmukh and Aher [28~~30~~]. Despite the fact that K controls stomatal opening and shutting, cell enlargement, and other critical physiological activities. Numerous research have examined importance of ~~how much~~ potassium on plant ~~affects~~ plant growth, however high K concentrations in soil solutions restrict magnesium uptake, leading to magnesium deficiency~~t~~ in plants [44~~5~~]. ~~Additionally, there are considerable differences in the mean values of K between sites as well as the mean values of K across all seasons.~~

Calcium (Ca) is crucial element in figuring out the soil's physical and chemical properties, especially its structure and pH. Clay platelets in soil colloids (caused by calcium ions) bind or aggregate to produce crumbs or peds. Frequent correlation has been reported between pH and ~~Acidity of the soil and~~ exchangeable calcium levels in soil. ~~are frequently correlated.~~ For example ~~I~~in the pH range of 7.0 to 8.5, Ca is accessible, while as low ~~.~~Exchangeable calcium levels were reported in ~~in the soil are often low when the soil has an~~ acidic pH [31~~2~~]. ~~During the present study, mean Ca concentration ranged from 668.33 to 984.39 (mg/Kg).~~ In the current study ~~M~~maximum Ca content reported during summer ~~(913.52 mg/Kg)~~ may be because of degradation of calcium rich waste such as bones and eggshells present in the kitchen waste and minimum was ~~(826.58 mg/kg)~~ reported during winter, which ~~-~~may be because of low rate of degradation of waste. Among sites, maximum Ca content reported at center ~~(957.67)~~ of dumping site may be attributed to the clayey nature of soils of dumpsite as shown in Table 3. ~~The mean values of Ca between all seasons were significantly different; furthermore the mean values of Ca between sites also vary significantly.~~

Magnesium (Mg) is found in both relatively soluble forms and ionic form, Mg^{2+}, which is attached to the soil colloidal complex. It has been reported that magnesium levels in acidic soils, especially sandy soils, are frequently low, while as neutral soils usually contain more exchangeable magnesium [31]. In this investigation among sites maximum Mg concentration reported at center of dumping site may be because of its less mobility due to presence of high organic waste and clayey nature of soil [45] and minimum at control site may be because of the presence of less organic matter as given in Table 3. Among seasons maximum Mg concentration reported during summer season may be because of less leaching and high rate of decomposition of organic waste and minimum during winter may be attributed to high leaching loss due to the high positive water balance [45]. An excessive amount of magnesium in the soil inhibits plant growth because it reduces the ability of roots to reduce TTC (2, 3, 5-Triphenyl Tetrazolium chloride) through the inhibition of the dehydrogenase enzyme linked to the respiratory systems [46].

Micronutrients are essential for sustaining the fertility of the soil and crop productivity. Compared to macronutrients, micronutrients are required in smaller amounts. Even if the supply of macronutrients is balanced and high yielding varieties are cultivated, lack of micronutrients will prevent maximum yield from being achieved [47]. In this study, the presence of Zn element in both naturally occurring materials, such as yard wastes and food wastes, as well as man-made materials like pigments, inks, metals, and plastics may account for the highest mean Zn value at the centre of the dumping site, while the lowest mean Zn value was reported at the control site, as shown in Table 4. Furthermore micronutrients are more tightly bonded to the soil at high pH than at low pH, the highest mean Zn concentration during the winter season may be attributed to low pH, whereas the lowest mean Zn concentration during the summer season may be attributed

to high pH [48]. Although Zn serves as a nutrient for plants, but is hazardous to them in larger amounts. Zn toxicity in plants results in growth inhibition, a reduction in biomass production, competition for nutrients, inactivation of enzymes, removal of vital components from functional locations, chlorosis, and, in certain situations, blockage of Fe translocation [49].

Ferric oxide (Fe_2O_3), also known as hematite, is the most prevalent form of Iron (Fe) in soils. It is exceedingly insoluble and gives soil its red colour. Commonly, the oxide form is hydrated [50]. In this study the maximum mean Fe concentration recorded at the centre of the dumping site may be attributed to the presence of organic matter in soils, as organic matter rich soils contain Fe in the reduced state (Fe^{2+}) or is adsorbed on the surface of soil particle [50]. The maximum iron concentration reported during winter season depicted in Table 4 may be attributed to low pH, because with increase in pH, ferrous ion (Fe^{2+}) gets converted to ferric ion (Fe^{3+}). Low solubility in solution makes the ferric ion (Fe^{3+}) compounds less bioavailable, and a minimum concentration was recorded during the summer [51]. The root cells and plasma membrane are damaged by the extra iron ions, which results in oxidative stress and increased formation of reactive oxygen species (ROS). Root cells may die as a result of ROS harming cellular components such lipids, proteins, and DNA [52]. Furthermore it, can also affect the uptake of other essential elements, such as phosphorus and zinc, by competing with the transporters that facilitate their uptake by the roots. This interference can lead to nutrient imbalances, reduced growth, and overall plant health [53].

Formatted: Indent: Left: 0"

Compared to other trace metals, copper (Cu) has extremely little mobility because it is firmly adsorbed onto soil particles. Due to this reduced mobility, Cu has a tendency to

Formatted: Indent: Left: 0"

concentrate on soil particles. Leaching of Cu occurs when the amount of Cu in the soil exceeds the capacity of the soil type to hold the copper ions. The pH, cation exchange capacity, organic matter content, and presence of iron, manganese, and aluminium oxides all affect how much Cu is present in soils. The amount of water in the soil affects the ability of the soil to store copper through biotic and abiotic oxidation-reduction reactions [31]. Among sites mean maximum concentration reported at center of dumping site may be due to the higher stability constants of Cu complexes with organic matter and minimum was reported at control site as shown in Table 4. Among seasons, maximum Cu concentration reported during winter season may be because of low pH, as at low pH Cu compounds are more soluble and minimum concentration of Cu reported during summer may be because of high pH, as high pH results in precipitation of copper (II) hydroxide (Cu $(OH)_2$) and Copper(I) hydroxide (CuOH) [54]. All phases of plant growth are affected by high Cu concentration toxicity, which has considerable detrimental impacts on morphology, physiological function, and molecular level [55]. Additionally, too much copper in the soil inhibits the activity of -amylase and invertase isoenzymes, which limits the ability of many plant species to break down reserve food supplies like starch and sucrose [56]. Maximum concentration of Mn reported at center of dumping site may be due to decomposition of plants and animals waste as well as animal excrement [57]. The lowest concentration of Mn was reported at control site as given in Table 4.

The highest Mn concentration was observed during the winter season, likely due to a decrease in soil pH. At low pH, trace elements are typically more soluble because of high desorption and low adsorption [31]. Conversely, the lowest Mn concentration was reported during the summer, which may attributed to increased adsorption [58]. Manganese is

essential for terrestrial plants, but excessive accumulation in leaves can cause toxicity and reduce crop yield.

Heavy metals are significant pollutants that enter in to environment through various channels, including landfill leachate, solid waste, and wastewater. According to Radfard et al. [59] and Kusin et al. [60], heavy metals in leachate contaminate soil and subsurface water supplies. As these contaminants move up the food chain, they impact human health and cause various ailments. Leachate is a critical source of pollution, releasing chemicals into soil and water sources, and its toxicity to human cells highlights its environmental impact [61]. In the current study among seasons max mean concentration of Pb was reported during summer season and min during winter. Among sites maximum concentration was reported at center of dumpsite and minimum was reported at control site. The values of Pb are higher than permissible limit (85mg/kg) at center during spring, summer and autumn, Inside during summer and autumn and outside during summer season as depicted in Table 5 and Fig 4.

Formatted: Indent: Left: 0", First line: 0"

Fig 4: Graphical representation of Pb (mg/kg) among different sites and seasons. S1 is spring, S2 is summer, S3 is autumn and S4 is winter, while as C1 is center, C2 is inside, C3 is outside and C4 is Control.

Formatted: Left, Indent: Left: 0", First line: 0", Right: 0", Space Before: 0 pt, After: 10 pt, Line spacing: Multiple 1.15 li, Widow/Orphan control, Pattern: Clear, Tab stops: Not at 0.49"

Formatted: Font: (Default) +Body (Calibri), 11 pt, Font color: Auto

Lead-acid batteries used in automobiles, rechargeable nickel-cadmium batteries, consumer electronics, glass, ceramics, and plastic products including PVC resins are sources of Pb in landfill leachate. Other sources of lead include soldered cans, pigments, brass and bronze products, rubber goods, spent motor oil, and wine bottle wrappers made of lead foil.

Formatted: Indent: Left: 0", First line: 0"

Among seasons max concentration of Cd was reported during summer season and min during winter. Among sites maximum concentration was reported at center of dumpsite, and minimum was reported at control site. Cadmium (Cd) levels at all sites exceeded the permissible

Formatted: Indent: First line: 0.49"

limit of 0.8 mg/kg across all seasons as depicted in Table 5 and Fig 5.

Fig 5. Graphical representation of Cd(mg/kg) among different sites and seasons. S1 is spring, S2 is summer, S3 is autumn and S4 is winter, while as C1 is center, C2 is inside, C3 is outside and C4 is Control.

The sources of cadmium in waste include nickel-cadmium batteries, pigments, stabilizers in plastics (primarily PVC), consumer electronics (notably the steel chassis of vintage TVs and radios that were cadmium-plated to prevent corrosion), antique appliances with cadmium-plated parts, textile dyes and paints, glass and ceramics, and pigments used in non-newspaper printing inks. Cadmium is one of the most ecotoxic metals, causing detrimental effects on soil health, plant metabolism, and the health of humans and animals [62]. Even at very low concentrations, chronic exposure to cadmium can lead to anemia, anosmia, cardiovascular diseases, and renal problems [63].

Ni was reported maximum during summer season and min during winter. Among sites maximum concentration was reported at center of dumpsite and minimum was reported at control site as depicted in Table 5 and Fig 6.

Fig 6. Graphical representation of Ni (mg/kg) among different sites and seasons. S1 is spring, S2 is summer, S3 is autumn and S4 is winter, while as C1 is center, C2 is inside, C3 is outside and C4 is Control.

The values of Ni were higher than the permissible limits (35 mg/kg) during summer season at all sites. The values were lower than the permissible limits during other seasons. The sources of Ni are lead cadmium batteries, electroplating materials, clinical wastes [64]. Because nickel is non-biodegradable and has the potential to accumulate in the food web, it is responsible for a number of health issues in humans, including allergic skin, headache, vertigo, nausea, vomiting, insomnia, and irritability [64, 65].

Formatted: Left, Space Before: 0 pt, After: 10 pt, Line spacing: Multiple 1.15 li, Widow/Orphan control, Tab stops: Not at 0.49"

Formatted: Font: (Default) +Body (Calibri), 11 pt, Font color: Auto

Formatted: Indent: First line: 0.49"

Among seasons max concentration of Cr was reported during summer season and min during winter. Among sites maximum concentration was reported at center of dumpsite, and minimum was reported at control site as depicted in Table 6 and Fig 7.

Fig 7. Graphical representation of Cr (mg/kg) among different sites and seasons. S1 is spring, S2 is summer, S3 is autumn and S4 is winter, while as C1 is center, C2 is inside, C3 is outside and C4 is Control.

The sources of Cr are electroplating and Cr containing wastes. The concentration of Cr was less than permissible value at all sites. The sorption properties of the soil, such as its clay, iron oxide, and organic matter contents, influence the mobility of Cr. Surface runoff can carry chromium to surface waters in both its soluble and precipitated forms. Chromium compounds, both soluble and unadsorbable, can seep into groundwater from the soil. As soil pH rises, Cr (VI) has a greater propensity to leach. However, the majority of Cr discharged into natural rivers is particle associated and eventually deposits into the sediment [66]. Human allergic dermatitis and chromium are related [67].

The value of Arsenic (As) ranged from 0.23 mg/kg to 18.50 mg/kg. Among seasons max concentration was reported during summer season and min during winter. Among sites maximum concentration was reported at center of dumpsite, and minimum was reported at control site as depicted in Table 6 and Fig 8.

Fig 8. Graphical representation of As (mg/kg) among different sites and seasons. S1 is spring, S2 is summer, S3 is autumn and S4 is winter, while as C1 is center, C2 is inside, C3 is outside and C4 is Control.

The permissible limit of As in soil is 20 mg/kg. The concentration of heavy metals, specifically Pb, Cd, Ni, Cr, and As, is higher during the summer than other seasons. The leaching of heavy metals is also higher during the summer because temperature influences it; as the temperature rises, the leaching of heavy metals also increases. However, the minimum concentration of heavy metals during winter season might be attributed to runoff effect during

Formatted: Left, Indent: First line: 0", Space Before: 0 pt, After: 10 pt, Line spacing: Multiple 1.15 li, Widow/Orphan control, Tab stops: Not at 0.49"

Formatted: Font: (Default) +Body (Calibri), 11 pt, Font color: Auto

Formatted: Left, Indent: First line: 0", Space Before: 0 pt, After: 10 pt, Line spacing: Multiple 1.15 li, Widow/Orphan control, Tab stops: Not at 0.49"

Formatted: Font: (Default) +Body (Calibri), 11 pt, Font color: Auto

winter season which facilitates the leaching of heavy metals from soil and contributes to the dilution of soil solution during winter season.

Heavy metal toxicity in plants severely hampers nutrient and water uptake, while intensifying oxidative damage, ultimately stunting plant growth. Cadmium (Cd) has been shown to interact with plants at the physio-biochemical level, leading to reduced growth. Cd toxicity disrupts nutrient and water absorption, heightens oxidative stress, impairs plant metabolism, and negatively impacts plant morphology and physiology [68]. Similarly, chromium (Cr) toxicity inhibits seed germination, slows plant growth, impairs enzymatic activities, and disrupts the photosynthetic machinery, leading to oxidative imbalances [69, 70].

Formatted: Justified, Line spacing: 1.5 lines

Lead (Pb) toxicity has been found to inhibit ATP production, elevate reactive oxygen species (ROS) levels, and cause DNA damage. It significantly affects germination, root elongation, plant growth, chlorophyll synthesis, and disturbs transcriptome sequencing [71]. Nickel (Ni) toxicity reduces seed germination, root and shoot development, biomass accumulation, and overall yield. It also causes chlorosis, necrosis, and disrupts physiological processes like photosynthesis and transpiration, while inducing oxidative stress. The threat posed by Ni increases with its concentration in the environment, particularly in soils [72].

Arsenic (As) exposure adversely affects plants at both biochemical and molecular levels, inhibiting key physiological processes such as overall growth, photosynthetic efficiency, and biomass production. Arsenic induces oxidative stress by increasing ROS production or reducing their elimination, leading to damage of lipids, proteins, and nucleic acids. It interferes with metabolic pathways, either by acting as a competitive inhibitor of phosphate (Pi) or by disrupting enzyme activities. Additionally, As inhibits seed germination, root and shoot development, and other critical early-stage processes during seedling growth [73].

Formatted: Normal (Web), Indent: First line: 0", Space Before: 0 pt, After: 0 pt, Line spacing: 1.5 lines, Widow/Orphan control, Tab stops: Not at 0.49"

Formatted: Font: (Default) Times New Roman, 12 pt, Font color: Auto, Pattern: Clear

Formatted: Indent: First line: 0"

Available management strategy for remediation of soil health:

Soil is very important asset for plant and human health, so the need of hour is to maintain the soil quality and prevent its degradation. The waste generated from various sources like houses, restaurants, agricultural fields, orchards is mostly organic (65-75%), which is diverted to landfill in huge amounts on daily basis can be segregated at source and then converted into compost. The compost generated can then be used to ameliorate the nutrient status of soil quality as well as to enhance the plant growth and development. Furthermore the compost generated will replace the chemical fertilizers used. The plan of utilization of microbial consortium technology for degradation of waste is depicted in flow chart given in Fig 9~~14~~.

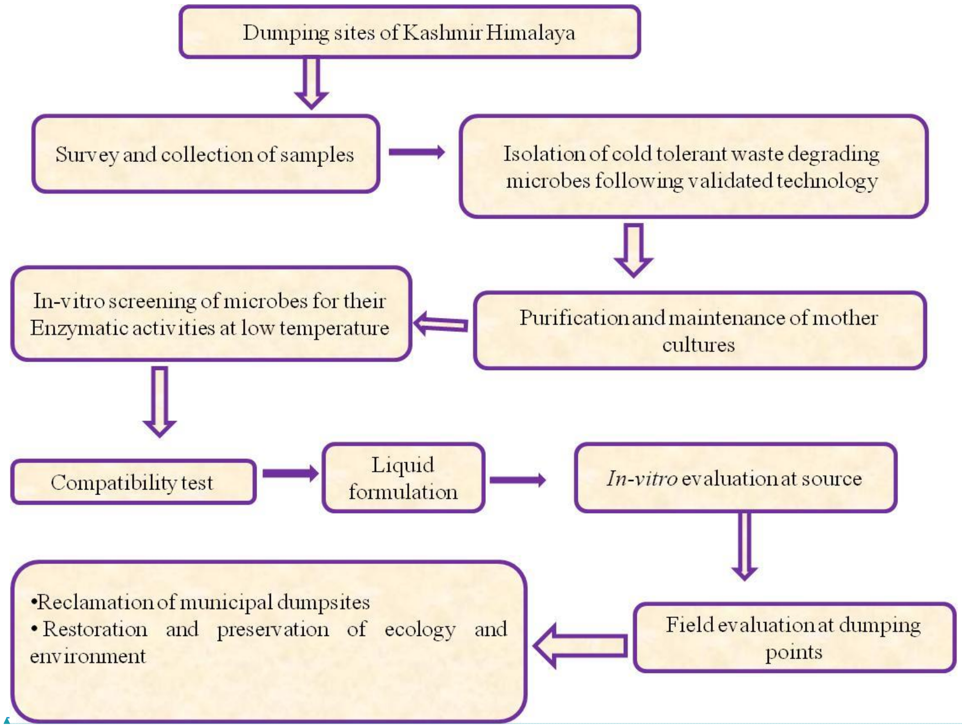

Fig 9~~14~~: Flow chat depicting management strategy for waste

Formatted: Font: (Default) Times New Roman, 12 pt, Bold, Font color: Text 1

Formatted: Left

Conclusion:

The soil plays a crucial role in controlling the flow of pollutants into the food chain. Population growth is causing waste generation to grow rapidly as well. Landfills have been a popular choice for residue dumping for the past 20 years. Our investigation has shown that, regardless of the time of year or the distance from the waste site, landfills seriously degrade the soil quality of nearby communities. pH was more acidic during the summer season and at center of the dumping site due to an increase in the production of organic acids, while as it was less acidic during the winter season and away from the dump site. The acidic pH results in a decrease in the availability of essential elements viz, N.P and K, however, it results in an increase in the availability of toxic heavy metals viz, Pb, Cd, Ni, Cr, As. The heavy metals were reported to be maximum during the summer season at all sites and minimum during the winter season. Among sites the heavy metals were maximum and decreased with increase in distance from the landfill. Our findings led to the conclusion that there is a need for global attention on improving or restoring soil health. Assessment of soil health indicators is expected to enhance our understanding of the factors underlying processes that contribute to sustainable agriculture. The leachate must be treated before disposal and that landfills must have adequate lining. Before disposing of rubbish, segregate non-biodegradable items like polythene and plastic bags since they take roughly 500 years to degrade. Biodegradable garbage must be handled using ecologically friendly techniques like anaerobic digestion and composting. The garbage that needs to be landfilled should only be non-biodegradable, inert, or unsuitable for recycling. MSW must not be combined with biomedical waste. Media should be used to inform people about trash management. The organic waste ought to be isolated at the point of generation and turned into compost using microbial processes.

Acknowledgement:

The authors acknowledge the support of SKUAST Kashmir and Division of Environmental sciences in carrying out the research.

Funding information: The authors would like also to extend their sincere appreciation to the Researchers Supporting Project number (RSPD2024R889), King Saud University, Riyadh, Saudi Arabia.

Author contribution: Shayesta Islam: methodology, writing; statistical analysis; Haleema Bano: methodology; Malik Ahsafsif Aziz: review and editing; Fahad Naseer Alotaibi: Review and Editing.

Declarations:

Consent for publication: Consent from all authors has been taken.

Conflict of interest: There is no conflict of interest between authors.

References:

1. IPBES (2018): The IPBES assessment report on land degradation and restoration. Montanarella, L., Scholes, R., and Brainich, A. (eds.). Secretariat of the Intergovernmental Science-Policy Platform on Biodiversity and Ecosystem Services, Bonn, Germany. 744.doi:https://doi.org/10.5281/zenodo.3237392"

1. IPBES. 2018. Summary for Policy makers of the Assessment Report on Land Degradation and Restoration of the Intergovernmental Science Policy Platform on Biodiversity and Ecosystem Services.

2. Stoessel F, Sonderegger T, Bayer P, Hellweg S. Assessing the Environmental Impacts of Soil Compaction in Life Cycle Assessment. The Science of the Total Environment. 2018 Feb Feb; 630: 913–921. doi: https://doi.org/10.1016/j.scitotenv.2018.02.222"

Formatted: No underline, Font color: Auto, Pattern: Clear

Formatted: Space After: 10 pt, Line spacing: Multiple 1.15 li, Adjust space between Latin and Asian text, Adjust space between Asian text and numbers

Formatted: Font: (Default) +Body (Calibri), 11 pt, Font color: Blue, Pattern: Clear

Formatted: Font: (Default) +Body (Calibri), 11 pt, Not Bold, Font color: Auto

Formatted: Font: (Default) +Body (Calibri), 11 pt, Font color: Auto

3. Brevik EC, Sauer TJ. The past, present, and future of soils and human health studies. Soil. 2015 Jan 06 Jan 06; 1: 35–46. doi: https://doi.org/10.5194/soil-1-35-2015"

4. Nielsen UN, Wall DH, Six J. Soil biodiversity and the environment. Annual Review of Environment and Resources. 2015 Nov 11Nov 11; 40: 63–90. doi:http://dx.doi.org/10.1146/annurev-environ-102014-021257"

5. Horton RE. The role of infiltration in the hydrologic cycle. Eos, Transactions of the American Geophysical Union. 1993 Jan 01 Jun 01; 14: 446–460. doi:http://dx.doi.org/10.1029/TR014i001p00446"

6. Ahmad R, Gao J, Li W, Yuanyuan Z, Gao Z, Khan A, et al. Response of soil nutrients, enzyme activities, and fungal communities to biochar availability in the rhizosphere of mountainous apple trees. Plant Soil. 2023 April 25 April 25. 489:277–293. doi:https://doi.org/10.1007/s11104-023-06016-4"

7. Hattori H, Chino M. Growth, cadmium, and zinc contents of wheat grown on various soils enriched with cadmium and zinc. Developments in Plant and Soil Sciences. 2001 Jan; 92:462–463.

8. Stein AJ. Global impacts of human mineral nutrition. Plant Soil. 2010 Oct; 335: 133–154.

9. Sahu N, Vasu D, Sahu A, Lal N, Singh SK. Strength of Microbes in Nutrient Cycling: A Key to Soil Health. In: Meena V, Mishra P, Bisht J, Pattanayak A, editors. Agriculturally Important Microbes for Sustainable Agriculture; Springer: Singapore; 2017. Pp. 69–86.

~~10. Ahmad R, Gao J, Gao Z, Khan A, Ali I, Fahad S. Influence of Biochar on Soil Nutrients and Associated Rhizobacterial Communities of Mountainous Apple Trees in Northern Loess Plateau China. Microorganisms. 2022 oct 20. 10: 2078.~~

711. Kaza S, Yao LC, Bhada-Tata P, Van Woerden F. What a Waste 2.0: A Global Snapshot of Solid Waste Management to 2050. Urban Development. Washington, DC: World Bank. 2018 sept 20. Available from: https://hdl.handle.net/10986/30317.

812. Bhat R.A., Dervash M.A., Mehmood M.A., Hakeem, K.R. Municipal Solid Waste Generation and its Management, a Growing Threat to Fragile Ecosystem in Kashmir Himalaya. American Journal of Environmental sciences. 20178.; 13: 388-397. doi:https://doi.org/10.3844/ajessp.2017.388.397"

9. Wani MA, Ahmad S. Challenges, issues of solid waste management in himalayas: A case study of srinagar city. African Journal of Basic and Applied Sciences. 2013; **5**(1): 25-29. doi:https://api.semanticscholar.org/CorpusID:110732616"

10. Nazir MI, Sundan S, Fayaz S, Dar MA, Raju JL. The effect of dumping site leachate on ground water quality: A case study of Srinagarcity, India. International Journal of Civil, Structural, Environmental and Infrastructure Engineering Research and Development. 2014; **4**(1): 1-8. https://api.semanticscholar.org/CorpusID:201821214"

113. Naveen B P, Mahapatra DM, Sitharam T G, SivapullaiahPV, Ramachandra TV. Physico-chemical and biological characterization of urban municipal landfill leachate.

Formatted: Indent: Left: 0", First line: 0"

Formatted: Font: (Default) Times New Roman, 12 pt

Formatted: Left, Indent: Left: 0", First line: 0", Line spacing: Multiple 1.15 li, Pattern: Clear

Formatted: Font: Not Italic

Formatted: Font: (Default) Times New Roman, 12 pt

Formatted: Font: (Default) Times New Roman, 12 pt, Font color: Custom Color(RGB(46,65,79)), Pattern: Clear (White)

Formatted: Font: (Default) Times New Roman, 12 pt

Formatted: Font: Not Italic

Formatted: Font: (Default) Times New Roman, 12 pt

Formatted: Font: Font color: Custom Color(RGB(46,65,79)), Pattern: Clear (White)

Formatted: Indent: Left: 0", First line: 0"

Environmental Pollution. 2017 Jan ~~Jan~~; 220: 1–12. doi:http://dx.doi.org/10.1016/j.envpol.2016.09.002"

12~~4~~4. Arunbabu V, Indu K S, Ramasamy EV. Leachate pollution index as an efective tool in determining the phytotoxicity of municipal solid waste leachate. **Waste Management . 2017 Jul 21~~Jul 21~~;** 68: 329–336. doi:10.1016/j.wasman.2017.07.012"

13~~5~~. Bolan N, Kunhikrishnan A, Thangarajan R, Kumpiene J, Park J, Makino T, et al. Remediation of heavy metal(loid)s contaminated soils – To mobilize or to immobilize. Journal of Hazardous Materials. ~~,~~ 2014 Feb 15; ~~Feb 15.~~ 226: 141-166. ~~,~~ doi:10.1016/j.jhazmat.2013.12.018"

14~~6~~. Sweety P. Impacts of Soil Pollution on Environment. Natural Volatiles and Essential Oils. 2021~~.~~; 8(5): 3314-3320.

15~~7~~. Ngatia L, Johnny M, Grace III, Moriasi D, Taylor R. Nitrogen and Phosphorus Eutrophication in Marine Ecosystems. In: Fouzia HB, editor. Monitoring of Marine Pollution. London, England: Intech open; 2019.pp. 1-17. doi:10.5772/intechopen.81869"

16~~8~~. Jahany M, Rezapour S. Assessment of the quality indices of soils irrigated with treated wastewater in a calcareous semi-arid environment. Ecological Indicators. 2020 Feb~~Feb~~; 109:105800. doi:https://doi.org/10.1016/j.ecolind.2019.105800"

17~~9~~. Rezapour S, Samadi A, Kalavrouziotis IK, Ghaemian N. Impact of the uncontrolled leakage of leachate from a municipal solid waste landfill on soil in a cultivated-calcareous environment. Waste Management. 2018 Dec ~~Dec~~; 82: 51–61. doi:10.1016/j.wasman.2018.10.013"

Formatted: Justified, Space Before: 0 pt, After: 10 pt, Line spacing: Double, No bullets or numbering, Pattern: Clear

Formatted: Font: (Default) +Body (Calibri), 11 pt, No underline, Font color: Auto, Pattern: Clear

Formatted: Font: (Default) +Body (Calibri), 11 pt, Font color: Auto

18~~20~~. Zaman MU, Pandit BA, Kumar R, Khan S. Challenges, Issues and Opportunities towards Management of Solid wastes in Indian Cities: A Case Study of Srinagar City. International Journal for Research in Applied Science & Engineering Technology . 2022 Dec~~Dec~~; 10(12). doi:10.22214/ijraset.2022.48196"

19~~21~~. Khan N A. Perspectives of Transport and Disposal of Municipal Solid Waste in Srinagar City. International Journal of Engineering Research and General Science. 2014 Jul~~Jun-Jul~~; **2**(4): 238-251. www.ijergs.org"

20~~2~~. Prihar SS, Sandhu BS. A rapid method for soil moisture determination. Soil Science. 1968; **105**(3): 142-144.

21~~3~~. Jackson ML. Soil chemical analysis. New Delhi: Prentice hall of India Private limited; 1973.

22~~4~~. Subbaih BV, Asija GL. A rapid procedure for the estimation of available nitrogen in the soil. Current Science Journal. 1956; 25: 259-266.

23~~5~~. Olsen SR, Cole C V, Watanabe FS, Dean LA. Estimation of available phosphorous in soils by extraction with sodium bicarbonate. Circular of the United States Department of Agriculture. Washington. D. C. 939. 1954.

24~~6~~. Chesin L, Yein CH. Turbid metric determination of available sulphur. Soil Science Society of America Proceedings. 1951; 15: 149-151.

25~~7~~. Lindsay WL, Norvell WA. Development of a DTPA-soil test for Zn, Fe, Mn and Cu. *Soil* Science Society of America Journal. 1978; **42**(3): 421-428.

268. Gomez KA, Gomez AA. Statistical Procedures for Agricultural Research. 2nd ed. New York: John Wiley and Sons; 1984.

279. Pereira DG, Afonso A, Medeiros FM. Over view of Friedman's test and post-hoc analysis. Communications in Statistics-Simulation and Computation. 2015 NovNov; 44(10): 2636–2653. doi:https://doi.org/10.1080/03610918.2014.931971"

2830. Deshmukh KK, Aher S P. Assessment of soil fertility around municipal solid waste disposal site near Sangamner City, Maharashtra, India. Current World Environment Journal. **2017 May 20May 20; 12**(2): 401-410. **doi:** http://dx.doi.org/10.12944/CWE.12.2.24"

3129. Brady NC, Weil RR. The nature and properties of soils. 13th edition. New Jersey: Pearson Education; 2002.

30. Havlin JL, Beaton JD, Tisdale SL, Nelson WL. *Soil Fertility and Fertilizers: An Introduction to Nutrient Management.* 8th ed. Pearson Education; 2013.

312. Ahmed I, Prasad Rao T V D, Hussain M. Quality assessment of soil at municipal solid waste dumpsite and possibilities of reclamation of land. International Journal of Innovative Science, Engineering and Technology. 2014 Sept; **1** (7): 274-294.

323. Sonneveld C, Voogt W. Plant Nutrition of Greenhouse Crops, Springer, ISBN 9048125316, New York, U.S.A. 2009.

334. Signore A, Serio F, Santamaria P. A targeted management of the nutrient solution in a soilless tomato crop according to plant needs. Frontiers in Plant Science. 2016 March 30; March 30. 7: 391. doi: https://doi.org/10.3389/fpls.2016.00391"

Formatted: Pattern: Clear (Background 1)

Formatted: Font: (Default) +Body (Calibri), 11 pt, No underline, Font color: Auto, Pattern: Clear

Formatted: Font: (Default) +Body (Calibri), 11 pt, Font color: Auto

Formatted: Indent: Left: 0", First line: 0"

Formatted: Font: (Default) Times New Roman, 12 pt, No underline, Font color: Auto, Pattern: Clear

Field Code Changed

Formatted: Font: (Default) Times New Roman, 12 pt, Pattern: Clear

Formatted: Font color: Auto, Pattern: Clear

345. Samarakoon UC, Weerasinghe PA, Weerakkody AP. Effect of Electrical Conductivity [EC] of the Nutrient Solution on Nutrient Uptake, Growth and Yield of Leaf Lettuce (Lactuca sativa L.) in Stationary Culture. Tropical Agricultural Research. 2006 Jan 18; Jan 18. 18: 13–21. doi:https://dl.nsf.gov.lk/handle/1/12138"

356. Praveena GS, Rao PVV. Impact of leachate on soil properties in the dumpsite: A case study of greater Visakhapatnam. International Journal of Engineering Research and General Science. 2016 Jan-Feb Jan-Feb; **4**(1): 2091-2730. doi:https://api.semanticscholar.org/CorpusID:106395088"

367. Krishna_MK, Chaitra BR Kumari J. Effect of municipal solid waste leachate on the quality of soil. International Journal of Engineering Science Invention. 2016 Jun; **5**(6): 69-72.

378. Zhang S, Lovdahl L, Grip H, Jansson P, Tong Y. Modelling the effects of mulching and fallow cropping on water balance in the Chinese Loess Plateau. Soil and Tillage Research. 2007 April; April. **93 (2)**: 283-298. doi:10.1016/j.still.2006.05.002"

389. Jokela JPY, Rintala JA. Anaerobic solubilisation of nitrogen from municipal solid waste (MSW). Reviews in Environmental Science and Biotechnology**. 2003 MarchMarch; 2**: 67-77. doi:https://doi.org/10.1023/B:RESB.0000022830.62176.36"

3940. Soheil RI, Hossien MH, Gholamreza S, Hassan E, Jamei M, Etesami H. Effects of composted municipal waste and its leachate on some soil chemical properties and corn plant responses. 2012 JanJan; **2**(6): 801-814. doi:http://www.ecisi.com"

Formatted: Font: Not Italic

Formatted: Font: Not Italic

Formatted: Space After: 10 pt, Line spacing: Multiple 1.15 li

Formatted: Font: 12 pt

Formatted: Font: (Default) Times New Roman, 12 pt

Formatted: Normal, Pattern: Clear

40~~1~~. Ahmad R, Muhammad D, Mussarat M, Fahad S, Ullah S, Ahmad T, et al. Effect of Different Levels of Nitrogen on Yield of Colocasia (Colocasia esculenta) at District Malakand Dargai. Open Journal of Soil Science. 2018 Feb 27; ~~Feb 27.~~ 8: 87-98. **doi:**10.4236/ojss.2018.82007"

41~~2~~. Sheikh S, Ishak CF. Effect of nitrogen fertilization on antioxidant activity of Mas cotek (Ficus deltoidea Jack). Journal of Medicinal Plants Studies. 2016 Jun 30; 4(4): 208–214.

42~~3~~. Sokka L, Antikainen R, Kauppi P. Flows of nitrogen and phosphorus in municipal waste: a substance flow analysis in Finland. Progress in Industrial Ecology. **2004 Jan 16~~Jan 16~~; 1**: 165 - 186. doi:10.1504/PIE.2004.004677"

43~~4~~. Fuentes B, Bolan N, Naidu R, Mora MDL. Phosphorus in organic waste soil system. Journal of Soil Science and Plant Nutrition. 2006; **6** (2): 64-83. doi:10.4067/S0718-27912006000200006"

44~~5~~. Trankner M, Tavakol E, Jakli B. Functioning of potassium and magnesium in photosynthesis, photosynthate translocation and photoprotection. Physiologia Plantarum. 2018 April 18 ~~April 18~~; 163: 414–431. doi:10.1111/ppl.12747"

45~~6~~. Gransee A, Fuhrs H. Magnesium mobility in soils as a challenge for soil and plant analysis, magnesium fertilization and root uptake under adverse growth conditions. Plant and Soil. 2012 Dec 29~~Dec 29~~; **368~~5~~**: 5-21. doi:10.1007/s11104-012-1567-y"

46~~7~~. Shimada N. Excess Injury of Calcium and Magnesium in the Crops. Japan Agricultural Research Quartely. 1973 Jul 01; . 7(3): 173-177.

4~~8~~7. Yadav BK. micronutrient status of soils under legume crops in arid region of Western Rajasthan, India. Academic Journal of Plant Sciences. 2011; **4** (3): 94-97.

48~~9~~. Havlin HL, Beaton JD, Tisdale SL, Nelson WL. Soil fertility and fertilizers-an introduction to nutrient management. 7th ed. New Delhi: PHI Learning Private Limited; 2010.

49~~50~~. Rout GR, Das P. Effect of Metal Toxicity on Plant Growth and Metabolism: I. Zinc. Agronomie. 2003 Jan~~Jan~~; 23 (1): 3–11. doi:10.1007/978-90-481-2666-8_53"

50~~1~~. Lucena JJ, Apaolaza LH. Iron nutrition in plants: an overview. Plant and Soil. 2017 Sept ~~Sept~~; 418 (1-2). doi:10.1007/s11104-017-3316-8"

51~~2~~. Landon JR. Booker tropical soil manual: A hand book of soil survey and agricultural land. Burnt Mill. UK: Booker agriculture international limited; 1991.

5~~3~~2. Onyango DA, Entila F, Dida MM, Ismail AM, Drame KN. Mechanistic understanding of iron toxicity tolerance in contrasting rice varieties from Africa: 1. Morpho-physiological and biochemical responses. Functional Plant Biology. 2019 Oct 29~~8 Oct 29~~; 46(1):93-105. doi:10.1071/FP18129"

53~~4~~. Meharg, A. 2011. Marschner's mineral nutrition of higher plants. Edited by P. Marschner. Amsterdam, Netherlands: Elsevier/ Academic Press. (2011), pp. 684, ~~US $124.95~~. ISBN 978-0-12-3849052. Experimental agriculture. 2012; 48(2):305. doi:10.1017/S001447971100130X"

54~~5~~. Das DK. Micronutrients: their behaviour in soils and plants. Kalyani publishers; 2000

55~~6~~. Adrees M, Ali S, Rizwan M, Ibrahim M, Abbas F, Farid M, et al. The Effect of Excess

Copper on Growth and Physiology of Important Food Crops: A Review. Environmental Science and pollution Research. 2015 Jun 26~~Jun 26~~; 22 (11): 8148–8162. doi:10.1007/s11356-015-4496-5”

56~~7~~. Mir AR, Pichtel J, Hayat S. Copper: Uptake, Toxicity and Tolerance in Plants and Management of Cu-Contaminated Soil. Biometals. 2021 Aug ~~Aug~~; 34 (4): 737–759. doi:10.1007/s10534-021-00306-z”

57~~8~~. Stokes, P. M., Campbell, P. G. C., Schroeder, W. H., Trick, C., France, R. L, et al. 1988. Manganese in the Canadian environment. Ottawa, Ontario: National Research Council of Canada, Associate Committee on Scientific Criteria for Environmental Quality.

58~~9~~. Neina D. The role of soil pH in plant nutrition and soil remediation. Applied and Environmental Soil Science. 2019 Nov 03 ~~Nov 03~~. pp. 9. doi: **https://doi.org/10.1155/2019/5794869**”

59~~60~~. Radfard M, Yunesian M, Nabizadeh R, Biglar H, Nazmara S, et al. Drinking water quality and arsenic health risk assessment in Sistan and Baluchestan, Southeastern Province, Iran. Human and Ecological Risk Assessment. 2018 May ~~May~~; 25(4): 1-17. doi:10.1080/10807039.2018.1458210”

60~~1~~. Kusin FM, Azani NNM, HasanS NMS, Sulong NA. Distribution of heavy metals and metalloid in surface sediments of heavily-mined area for bauxite ore in Pengerang, Malaysia and associated risk assessment. CATENA. 2018 Jun; 165: 454–464. doi:10.1016/J.CATENA.2018.02.029”

61~~2~~. World Health Organization. Manganese in Drinking-Water: Background Document for Development of WHO Guidelines for Drink ing-Water Quality 2004.Available from:

Formatted: Pattern: Clear

Formatted: Font: (Default) +Body (Calibri), 11 pt, Font color: Auto

Formatted: Indent: Left: 0", First line: 0"

Formatted: Indent: Left: 0", First line: 0", Space Before: 6 pt, After: 6 pt

Formatted: Font: Not Bold

Formatted: Font: Not Bold

Formatted: Font: (Default) +Body (Calibri), 11 pt, No underline, Font color: Auto

Formatted: Font: (Default) +Body (Calibri), 11 pt, Font color: Auto

https://iris.who.int/handle/10665/75376.

623. Kabata Pendias AK. Trace element in soils and plants. Boca Raton, FL, USA: CRC Press; 2000. doi:https://doi.org/10.1201/b10158"

634. Sharma RK, Agrawal M, Marshall F. Heavy metal contamination in vegetables grown in wastewater irrigated areas of Varanasi, India. Bulletin of Environmental Contamination and Toxicology. 2006 SeptSept; 77 (2):312-318. doi:10.1007/s00128-006-1065-0"

645. Das K K, Das SN, Dhundasi SA. Nickel, its adverse health effects & oxidative stress. Indian Journal of Medical Research. 2008 Oct Oct; 128: 412-425.

665. Ogundiran OO, Afolabi TAA. Assessment of the physicochemical parameters and heavy metal toxicity of leachates from municipal solid waste open dumpsite. International Journal of Environmental Science and Technology. 2008 March March; 5 (2): 243-250. doi:10.1007/BF03326018"

667. Smith LA, Means JL, Chenetal A. Remedial Options for Metals-Contaminated Sites. , Boca Raton, Fla, USA: Lewis Publishers; 1995.

678. Scragg A. Environmental Biotechnology. 2nd ed. Oxford, UK: Oxford University Press; , 2006.

68. Haider FU, Liqun C, Jeffrey A, Coulter, Cheema SA, Wu J, Zhang R, Wenjun M, Farooq M. Cadmium toxicity in plants: Impacts and remediation strategies. Ecotoxicology and Environmental Safety. 2021; 211: doi: https://doi.org/10.1016/j.ecoenv.2020.111887"

69. Ugwu EI, Agunwamba JCA. Review on the Applicability of Activated Carbon Derived from Plant Biomass in Adsorption of Chromium, Copper, and Zinc from Industrial Wastewater. *Environ. Monit. Assess.* 2020;192:240. doi:10.1007/s10661-020-8162-0"

Formatted: Indent: First line: 0"

70. Patra DK, Pradhan C, Patra HK. Chromium Bioaccumulation, Oxidative Stress Metabolism and Oil Content in Lemon Grass *Cymbopogon Flexuosus* (Nees Ex Steud.) W. Watson Grown in Chromium Rich over Burden Soil of Sukinda Chromite Mine, India. *Chemosphere*. 2019;218: 1082–1088. doi:10.1016/j.chemosphere.2018.11.211"

71. Rahman S, Qin A, Zain M, Mushtaq Z, Mehmood F, Riaz L, Naveed S, Ansari MJ, Saeed M, Ahmad I, Shehzad M. Pb uptake, accumulation, and translocation in plants: Plant physiological, biochemical, and molecular response: A review. Heliyon. 2024; 10(6). doi:https://doi.org/10.1016/j.heliyon.2024.e27724"

72. Hassan MU, Chattha MU, Khan I, Chattha MB, Aamer M, Nawaz M, Ali A, Khan MAU, Khan TA. Nickel toxicity in plants: reasons, toxic effects, tolerance mechanisms, and remediation possibilities-a review. Environ Sci Pollut Res Int. 2019; 26(13):12673-12688. doi:10.1007/s11356-019-04892-x"

73. Abbas G, Murtaza B, Bibi I, Shahid M, Niazi NK, Khan MI, Amjad M, Hussain M; Natasha. Arsenic Uptake, Toxicity, Detoxification, and Speciation in Plants: Physiological, Biochemical, and Molecular Aspects. Int J Environ Res Public Health. 2018;15(1):59. doi:10.3390/ijerph15010059"

Rebuttal

Review Comments to the Author Please use the space provided to explain your answers to the questions above. You may also include additional comments for the author, including concerns about dual publication, research ethics, or publication ethics. (Please upload your review as an attachment if it exceeds 20,000 characters)

Reviewer #2: (No Response)

Reviewer #3:

Dear Authors,

The subject of the study is interesting and topical, with scientific and practical importance. The authors took into account the recommendations of the reviewers and made the necessary corrections. The article has been considerably improved.

The following aspects are brought to the attention of the authors.

1.Article structure

Page 13, row 101

“Study area:”

Page 14

“Methodology:”

It is recommended to check and revise the structure of chapters (chapter titles), according to the one recommended in the Submission Guidelines, PLOS ONE.

e.g.

“Manuscript Organization”

“Materials and Methods”

Response: All the chapter titles and subtitles have been revised according to the guidelines of PLOS ONE

2.Writing ionic forms

Page 27, row 348

“Mg2+” instead of “Mg2+”

"2+" as index

Page 30, row 402

“Fe2O3” instead of “Fe2O3”

Page 31, row 404

“Fe2+” instead of “Fe++”

"2+" as index

row 409

“Fe3+” instead of “Fe3+”

"3+" as index

It is recommended to use the Word Equation Editor to write the ionic forms correctly

Response: All the corrections suggested have been made and word equation editor has been used to write ionic forms.

3. Presentation of the Figures

Figure 14 is presented on page 41

Until figure 14, no other figure is presented in the content of the article.

References are made to the other figures, but they are presented at the end, starting with page 53.

Please check and revise, if necessary, according to the Submission Guidelines, PLOS ONE

Response: According to submission guidelines of PLOS ONE, figures are not to be included in the main manuscript file, figures are to be prepared as individual file. The Fig 14 has now also been removed from main manuscript. The captions of all figures have been inserted in the text of manuscript immediately after the paragraph in which it is cited.

4. References Please check the References chapter, and revise, if necessary, according to the Submission Guidelines, PLOS ONE

Response: References have been revised according to submission guidelines

Reviewer #4: PLOS ONE- Landfill leachate: an invisible threat to soil quality of temperate Himalayas

Manuscript Number: PONE-D-24-19939R1

Overall comments ¬

The manuscript is informative. I am very positive about the manuscript, but it still requires additional work and minor editing before being considered for publication.

Please ensure that your manuscript meets PLOS ONE's style requirements. The paper needs a total revision; especially the results section and the discussion section should be separate.

Response: Separated result and discussion section

Please review your reference list to ensure that it is complete and correct. Please follow the references style of plos journal formet. Provide DOI no. of the references and try to add recent references. For example 'Amoakwah E, Arthur E, Frimpong KA, Islam KR. Biochar Amendment Influences Tropical Soil Carbon and Nitrogen Lability. Journal of Soil Science and Plant Nutrition. 2021; 21: 3567– 3579. http://dx.doi. org/10.1007/s42729-021-00628-4''

Response: Revised reference section according to journal guidelines

Specific comments

1. Please check no. of words in Abstract, not exceed 300 words according to journal style. Please ensure that your manuscript meets PLOS ONE's style requirements

Response: Abstract has been reduced to 300 words only

2. Please insert the title of the figure or table just below the text. Please ensure that your manuscript meets PLOS ONE's style requirements

Response: Title of figures and tables have been inserted in the text as per journal guidelines.

3. It would be better to separate the results section and the discussion section. Please check the PLOS ONE's style.

Response: Separated results and discussion section.

4. Rewrite the discussion section.

Response: Modified the discussion section

5. Please follow the reference style of PLOS ONE's style. Provide DOI no. of the references and try to add recent references. Please review your reference list to ensure that it is complete and correct. For example ''Amoakwah E, Arthur E, Frimpong KA, Islam KR. Biochar Amendment Influences Tropical Soil Carbon and Nitrogen Lability. Journal of Soil Science and Plant Nutrition. 2021; 21: 3567– 3579. http://dx.doi. org/10.1007/s42729-021-00628-4''

Response: Revised reference section as per journal guidelines

6. Please check the front style of headline, sub-headline and text front. Please check the PLOS ONE's style.

Response: Followed PLOS one font style for each section.

7. If possible try to reduce no of figures. No. of fig 14 is too high.

Response: Reduced the number of figures to 9

8. Please check line no. 58. Remove double full stop.

Response: Done

9. Please check line no. 84. Close bracket

Response: Done

10. Please check line no. 90. Check the space

Response: Done

11. Please check line no. 108. Remove bracket

Response: Done

12. Please check line no. 121. Edit space

Response: Done

13. Please check line no. 121, 150, 121. Edit space

Response: Done

14. Please check line no. 312, 314, 352, and 354 and so on. Why are the numbers bold?

Response: Done

15. Please check Table 3, column 8. Some words are overlapped

Response: The line numbers have been overlapped with the table values, corrected

16. Please check line no. 474, 476,506. Please check the line spacing

Response: Done

17. Please provide declaration, consent for publication and conflict of interest from the manuscript and add in a separate file to PLOS ONE's online system. Only Acknowledgement and Author contributions would be attached with the article.

Response: Done as per suggestion

18. Please ensure that your manuscript meets PLOS ONE's style requirements.

Response: Modified as per journal guidelines

Reviewer #5: A brief study on "Landfill leachate: an invisible threat to soil quality of temperate Himalayas" has been conducted by the authors. The major concern is the content of toxic elements like Pb, Cd, Ni, As and Cr in the leachate. This will help the environmentalist and planner of the region for sustainable development goals for the locality. Please mention the safe limit of all the parameters studied in this work for soil just like Pb, Cd, Ni, As and Cr.

Response: Safe limits for all paramaters have been incorporated

Reviewer #6:

1. Abstract: Grammatical correction is required in lines- 15, 19, 29, 30, 33-36, 41. Punctuation correction is required in lines-30, 34.

Response: Corrected

2. Introduction: Too much narration on soil health in the introduction section (lines 54-64) while less description of the keywords - landfill and leachate. An individual paragraph on Achan landfill and leachate could be helpful for better understanding and reading. Coordination of the paragraphs in the introduction section is suggested. Grammatical and punctuation correction is required in all through the introduction section (lines- 45-47, 52,54,58, 60-68, 81, 82).

Response: Line numbers from 54-64 have been removed from the manuscript. Separate paragraph on Achan landfill and leachate have been added. Grammatical and punctuation errors have been corrected.

3. Study area: No indication of the area in the sampling site. Precise information about the sampling site is needed. Grammatical and punctuation correction is required in the study area section- 108,112,113

Response: Area under landfill have been added and corrections are made.

4. Methodology: No indication of year of sampling. Why sampling was done in those locations? Are those sites the leachate outflow sites? Authors analyzed only soils. But analysis of both soil and leachate would give a clear understanding about the impact of landfill leachate on soil quality. Grammatical and punctuation correction is required in the methodology section (lines-120-122, 140)

Response: Mentioned year of sampling and reasons for soil sampling, Grammatical and punctuation correction done

5. Result and discussion: I suggest to establish a relationship among pH, EC and element concentration.

Response: Incorporated correlation table in results section (Table 7)

While revising your submission, please upload your figure files to the Preflight Analysis and Conversion Engine (PACE) digital diagnostic tool, https://pacev2.apexcovantage.com/. PACE helps ensure that figures meet PLOS requirements. To use PACE, you must first register as a user. Registration is free. Then, login and navigate to the UPLOAD tab, where you will find detailed instructions on how to use the tool. If you encounter any issues or have any questions when using PACE, please email PLOS at figures@plos.org. Please note that Supporting Information files do not need this step.

Response: Done
