# Supplementary material for: Landfill leachate: An invisible threat to soil quality of temperate Himalayas
Source: PLoS One. 2024 Nov 19;19(11):e0314006. doi: 10.1371/journal.pone.0314006 (PMC11575778; doi:10.1371/journal.pone.0314006)
Supplement: Attachment — Submitted filename: Landfill Comments.docx [file pone.0314006.s002.docx]

**Comments**

I have read the exhaustive article on “**Landfill leachate: an invisible threat to soil quality of temperate Himalayas**”. In this article, the authors thoroughly document previous literature. However, it is necessary to cite the following research articles in the introduction and methodology sections before publishing.

1. DOI: 10.1007/s11104-023-06016-4
2. DOI: 10.3390/microorganisms10102078
3. DOI: 10.4236/ojss.2018.82007
