# Supplementary material for: Landfill leachate: An invisible threat to soil quality of temperate Himalayas
Source: PLoS One. 2024 Nov 19;19(11):e0314006. doi: 10.1371/journal.pone.0314006 (PMC11575778; doi:10.1371/journal.pone.0314006)
Supplement: Attachment — Submitted filename: Response to reviewers (Sec Rev).docx [file pone.0314006.s005.docx]

**Rebuttal**

Review Comments to the Author Please use the space provided to explain your answers to the questions above. You may also include additional comments for the author, including concerns about dual publication, research ethics, or publication ethics. (Please upload your review as an attachment if it exceeds 20,000 characters)

Reviewer #2: (No Response)

Reviewer #3:

Dear Authors,

The subject of the study is interesting and topical, with scientific and practical importance. The authors took into account the recommendations of the reviewers and made the necessary corrections. The article has been considerably improved.

The following aspects are brought to the attention of the authors.

1.Article structure

Page 13, row 101

“Study area:”

Page 14

“Methodology:”

It is recommended to check and revise the structure of chapters (chapter titles), according to the one recommended in the Submission Guidelines, PLOS ONE.

e.g.

“Manuscript Organization”

“Materials and Methods”

**Response: All the chapter titles and subtitles have been revised according to the guidelines of PLOS ONE**

2.Writing ionic forms

Page 27, row 348

“Mg2+” instead of “Mg2+”

"2+" as index

Page 30, row 402

“Fe2O3” instead of “Fe2O3”

Page 31, row 404

“Fe2+” instead of “Fe++”

"2+" as index

row 409

“Fe3+” instead of “Fe3+”

"3+" as index

It is recommended to use the Word Equation Editor to write the ionic forms correctly

**Response: All the corrections suggested have been made and word equation editor has been used to write ionic forms.**

3. Presentation of the Figures

Figure 14 is presented on page 41

Until figure 14, no other figure is presented in the content of the article.

References are made to the other figures, but they are presented at the end, starting with page 53.

Please check and revise, if necessary, according to the Submission Guidelines, PLOS ONE

**Response: According to submission guidelines of PLOS ONE, figures are not to be included in the main manuscript file, figures are to be prepared as individual file. The Fig 14 has now also been removed from main manuscript. The captions of all figures have been inserted in the text of manuscript immediately after the paragraph in which it is cited.**

4. References Please check the References chapter, and revise, if necessary, according to the Submission Guidelines, PLOS ONE

**Response: References have been revised according to submission guidelines**

Reviewer #4: PLOS ONE- Landfill leachate: an invisible threat to soil quality of temperate Himalayas

Manuscript Number: PONE-D-24-19939R1

Overall comments ¬

The manuscript is informative. I am very positive about the manuscript, but it still requires additional work and minor editing before being considered for publication.

Please ensure that your manuscript meets PLOS ONE's style requirements. The paper needs a total revision; especially the results section and the discussion section should be separate.

**Response: Separated result and discussion section**

Please review your reference list to ensure that it is complete and correct. Please follow the references style of plos journal formet. Provide DOI no. of the references and try to add recent references. For example ‘Amoakwah E, Arthur E, Frimpong KA, Islam KR. Biochar Amendment Influences Tropical Soil Carbon and Nitrogen Lability. Journal of Soil Science and Plant Nutrition. 2021; 21: 3567– 3579. http://dx.doi. org/10.1007/s42729-021-00628-4’’

**Response: Revised reference section according to journal guidelines**

Specific comments

1. Please check no. of words in Abstract, not exceed 300 words according to journal style. Please ensure that your manuscript meets PLOS ONE's style requirements

**Response: Abstract has been reduced to 300 words only**

2. Please insert the title of the figure or table just below the text. Please ensure that your manuscript meets PLOS ONE's style requirements

**Response: Title of figures and tables have been inserted in the text as per journal guidelines.**

3. It would be better to separate the results section and the discussion section. Please check the PLOS ONE's style.

**Response: Separated results and discussion section.**

4. Rewrite the discussion section.

**Response: Modified the discussion section**

5. Please follow the reference style of PLOS ONE's style. Provide DOI no. of the references and try to add recent references. Please review your reference list to ensure that it is complete and correct. For example ‘’Amoakwah E, Arthur E, Frimpong KA, Islam KR. Biochar Amendment Influences Tropical Soil Carbon and Nitrogen Lability. Journal of Soil Science and Plant Nutrition. 2021; 21: 3567– 3579. http://dx.doi. org/10.1007/s42729-021-00628-4’’

**Response: Revised reference section as per journal guidelines**

6. Please check the front style of headline, sub-headline and text front. Please check the PLOS ONE's style.

**Response: Followed PLOS one font style for each section.**

7. If possible try to reduce no of figures. No. of fig 14 is too high.

**Response: Reduced the number of figures to 9**

8. Please check line no. 58. Remove double full stop.

**Response: Done**

9. Please check line no. 84. Close bracket

**Response: Done**

10. Please check line no. 90. Check the space

**Response: Done**

11. Please check line no. 108. Remove bracket

**Response: Done**

12. Please check line no. 121. Edit space

**Response: Done**

13. Please check line no. 121, 150, 121. Edit space

**Response: Done**

14. Please check line no. 312, 314, 352, and 354 and so on. Why are the numbers bold?

**Response: Done**

15. Please check Table 3, column 8. Some words are overlapped

**Response: The line numbers have been overlapped with the table values, corrected**

16. Please check line no. 474, 476,506. Please check the line spacing

**Response: Done**

17. Please provide declaration, consent for publication and conflict of interest from the manuscript and add in a separate file to PLOS ONE's online system. Only Acknowledgement and Author contributions would be attached with the article.

**Response: Done as per suggestion**

18. Please ensure that your manuscript meets PLOS ONE's style requirements.

**Response: Modified as per journal guidelines**

Reviewer #5: A brief study on "Landfill leachate: an invisible threat to soil quality of temperate Himalayas" has been conducted by the authors. The major concern is the content of toxic elements like Pb, Cd, Ni, As and Cr in the leachate. This will help the environmentalist and planner of the region for sustainable development goals for the locality. Please mention the safe limit of all the parameters studied in this work for soil just like Pb, Cd, Ni, As and Cr.

**Response: Safe limits for all paramaters have been incorporated**

Reviewer #6:

1. Abstract: Grammatical correction is required in lines- 15, 19, 29, 30, 33-36, 41. Punctuation correction is required in lines-30, 34.

**Response: Corrected**

2. Introduction: Too much narration on soil health in the introduction section (lines 54-64) while less description of the keywords - landfill and leachate. An individual paragraph on Achan landfill and leachate could be helpful for better understanding and reading. Coordination of the paragraphs in the introduction section is suggested. Grammatical and punctuation correction is required in all through the introduction section (lines- 45-47, 52,54,58, 60-68, 81, 82).

**Response: Line numbers from 54-64 have been removed from the manuscript. Separate paragraph on Achan landfill and leachate have been added. Grammatical and punctuation errors have been corrected.**

3. Study area: No indication of the area in the sampling site. Precise information about the sampling site is needed. Grammatical and punctuation correction is required in the study area section- 108,112,113

**Response: Area under landfill have been added and corrections are made.**

4. Methodology: No indication of year of sampling. Why sampling was done in those locations? Are those sites the leachate outflow sites? Authors analyzed only soils. But analysis of both soil and leachate would give a clear understanding about the impact of landfill leachate on soil quality. Grammatical and punctuation correction is required in the methodology section (lines- 120-122, 140)

**Response: Mentioned year of sampling and reasons for soil sampling, Grammatical and punctuation correction done**

5. Result and discussion: I suggest to establish a relationship among pH, EC and element concentration.

**Response: Incorporated correlation table in results section (Table 7)**

While revising your submission, please upload your figure files to the Preflight Analysis and Conversion Engine (PACE) digital diagnostic tool, https://pacev2.apexcovantage.com/. PACE helps ensure that figures meet PLOS requirements. To use PACE, you must first register as a user. Registration is free. Then, login and navigate to the UPLOAD tab, where you will find detailed instructions on how to use the tool. If you encounter any issues or have any questions when using PACE, please email PLOS at figures@plos.org. Please note that Supporting Information files do not need this step.

**Response: Done**
