# Supplementary material for: Landfill leachate: An invisible threat to soil quality of temperate Himalayas
Source: PLoS One. 2024 Nov 19;19(11):e0314006. doi: 10.1371/journal.pone.0314006 (PMC11575778; doi:10.1371/journal.pone.0314006)
Supplement: Attachment — Submitted filename: Response to reviewers (3rd Rev).docx [file pone.0314006.s007.docx]

**Rebuttal**

6. Review Comments to the Author

Please use the space provided to explain your answers to the questions above. You may also include additional comments for the author, including concerns about dual publication, research ethics, or publication ethics. (Please upload your review as an attachment if it exceeds 20,000 characters)

Reviewer #3: Dear Authors,

The subject of the study is interesting and topical, with scientific and practical importance.
The authors took into account the recommendations of the reviewers and made the necessary corrections.
The article has been considerably improved.

The following minor aspects are brought to the attention of the authors.


1.
Space between the bibliographic source number and the previous word
e.g.
"Gomez [16]."

Reply: Corrected

2.
Central positioning of all values in the table columns.

e.g.
Table 1

Reply: Done

3.
Space between the value and the unit of measure
e.g.
"0.98 mg/kg"

Reply: Done

Some suggestions were made in the article.

Reply: Incorporated all suggestions

Reviewer #6: The authors have revised the manuscript as per my comments and suggestions. Now the manuscript has become sound scientifically.
